# Identification and characterization of eccDNA-driven genes in humans

**DOI:** 10.1371/journal.pone.0324438

**Published:** 2025-06-06

**Authors:** Yuxi Gu, Yidan Song, Jun Liu

**Affiliations:** State Key Laboratory of Oral Diseases and National Clinical Research Center for Oral Diseases, Department of Orthodontics, West China Hospital of Stomatology, Sichuan University, Chengdu, Sichuan, China; Shandong Agricultural University, CHINA

## Abstract

Extrachromosomal circular DNA (eccDNA) amplification promotes oncogene expression and cancer development. However, the global transcriptional landscape mediated by eccDNA has not yet been extensively profiled. Here we report a comprehensive analysis spanning cancer, non-cancerous disease and health by developing a new approach to catalog eccDNA-driven genes (EDGs). EDG expression is significantly higher than the average level. Our study identifies 27 common EDGs (CEDGs) existing in most cancer types. Integrated analysis of the CEDGs on gene expression, pathway and network, genetic alteration, epigenetic state, single-cell state, immune infiltration, microbiome and clinically-related features reveals their crucial roles in tumorigenesis and clinical significance. A 17-gene CEDG signature and nomogram was constructed to predict pan-cancer patients’ outcomes. By a novel eccDriver algorithm, 432 candidate eccDNA-driven drivers were identified. We show the candidate drivers regulate five major biological processes including immune system process, developmental process, metabolic process, cell cycle and division, and regulation of transport. 275 of the 432 candidate drivers are clinically actionable with approved drugs. We also demonstrate that eccDNA generation is associated with DNA methylation. Our study reveals general EDG function in humans and provides the most comprehensive discovery of eccDNA-driven driver genes in cancer and non-cancerous diseases to date for future research and application.

## Introduction

Extrachromosomal circular DNA (eccDNA) is nonplasmid circular DNA structure independent of chromosomes and ranges from ~100 bases to several megabases [1–5]. eccDNA has been widely reported in eukaryotes and prevalent in cancer development [5,6]. eccDNA lacks typical high-order compaction and promotes accessibility of chromatin [1]. Oncogene amplification based on eccDNA has been found common in cancers, achieving high copy oncogene number and diversity while elevating oncogene transcription [1,7,8]. Aberrant enhancer activity plays a crucial role in oncogene regulation for tumor formation, maintenance, and development [9–11]. Genomic rearrangements reposit enhancers near proto-oncogenes to activate them [12,13]. eccDNA provides the opportunity for oncogenic regulatory rewiring [1]. Neighboring enhancers co-amplify with oncogenes and change the topological connections [2]. A cluster of ~10–100 eccDNAs drive intermolecular enhancer-promoter interactions, amplifying oncogene expression [14]. eccDNA is also common in human healthy samples and development of non-cancerous diseases [15–17].

However, our knowledge on the transcriptional landscape mediated by eccDNA is far from complete. Over the past decade, numerous impressive studies have motivated the discovery and characterization of cancer driver genes, while a thorough profile from eccDNA view is lacking [18–20]. The same is true in non-cancerous diseases. Existing methods for identification of eccDNA-related genes specialize in particular techniques and simply counting genes overlapped with eccDNAs, ignoring if these genes could be transcribed [16,17,21,22]. Meanwhile, genes targeted by enhancers on eccDNAs are often overlooked [16,17,21]. Several databases include enhancer information on eccDNAs but by the “overlap” strategy, mixing with truncated elements without acting locus [22]. As fractions without core regions like genes without promoter sequence and enhancers without transcription factor binding site (TFBS) or other core sequences may fail to implement their original function, these methods are not appropriate for analyzing eccDNA-mediated transcription change [23–25]. Besides, most studies for characterization of eccDNA-related genes are limited to GO and KEGG analysis [16,17,26]. Therefore, a method for more thorough and accurate exploration of eccDNA-mediated transcription is necessary.

Recent methods for identification of cancer driver genes can be mainly divided into three categories: frequency-based, function-based, and network-based methods [27–29]. Network-based methods have been reported outperformed other methods [30]. For the graph theory concepts in network, different node centrality measures have been developed for identification of functionally-critical network. For degree centrality, driver genes usually have a high degree, while driver identification algorithms are often biased for selecting high-degree nodes, ignoring low degree but important nodes [31]. The high computational complexity of betweenness and closeness centrality makes it difficult to manage large-scale networks. The semi-local centrality combated the centrality-bias by constructing a semi-local network, which also decreases the computational complexity [32]. Global centrality like betweenness and closeness centrality require full knowledge of the network topology, which is challenged by time complexity and scale poorly for large networks. In contrast, local centrality like degree centrality relies solely on direct neighborhood information, offering computational efficiency [31]. However, the limited scope of local centrality usually causes centrality bias for ignoring broader structural influences. Semi-local centrality, as a balance, incorporates multi-hop neighborhood information to capture a tunable semi-local network without requiring global traversal, thereby balancing accuracy and time complexity [32]. This intermediate scope makes semi-local approaches practical for large-scale networks where purely global or local methods are inadequate. However, the original semi-local centrality consults the information of the four-hop neighbors and equals the importance of the nodes of different layers. The accuracy and computational complexity still need to be improved.

DNA methylation predominantly occurs in promoter regions through adding a methyl group to the cytosine residues in CpG dinucleotides [33,34]. Aberrant DNA methylation patterns have been extensively studied in cancer and other diseases [34,35]. DNA methylation has been linked to gene expression and genomic instability and eccDNA has been reported to be enriched at CpG islands [36–39]. However, our knowledge of the association of eccDNA and DNA methylation is still limited.

Here, we set out to (1) develop an effective approach for EDG identification and prioritization, (2) characterize their global landscape, (3) find out their biological functions and underlying mechanisms in cancer, (4) explore their potential clinical implications.

## Materials and methods

### Ethics statement

No additional ethics approval was needed. Data was downloaded from published studies or databases. Each study is referenced and details on ethics approval are available in each manuscript.

### Data collection

eccDNA data was collected form eccatlas database and other research studies [7,15–17,22,40,41]. We restricted our analysis to datasets which contains enough numbers of eccDNAs (>100) for effective and accurate analysis. Other criterions are as follows: ecDNA and eccDNA for eccDNA type; WGS, ATAC-seq, circle-seq, nanocircle, SMOOTH-seq and other for library type. eccDNAs identified in BMSCs and its differentiation into OBs, ACs and CCs of our own datasets were available in GSE261856. Enhancers were from GeneHancer [42]. Transcript information was from Esembl Genes in table browser [43]. RNA expression data of HeLa cells was collected from GSE183535 [44].

The tumor associated data used comprised mRNA Seq data, clinical data, single nucleotide variation (SNV) data, copy number variation (CNV) data, methylation data were collected from The Cancer Genome Atlas (TCGA) and university of california santa cruz (UCSC) Xena (http://xena.ucsc.edu/) [45]. ImmuCellAI was used to estimate the immune cell abundance [46]. The protein expression data was collected from the TCPA database [47]. The drug-related data was collected from the the Genomics of Drug Sensitivity in Cancer (GDSC) and The Cancer Therapeutics Response Portal (CTRP) database [48,49]. Microbiome data was collected from The Cancer Microbiome Atlas (TCMA) [50]. The information of CpG islands were from UCSC Table Browser [43]. Me-QTL data was from Pancan-meQTL [51].

### EDG discover approach and curation

For eccDNA-regulated gene (ERG) identification, bedtools were applied to identify full enhancer elements on eccDNAs. The “full” strategy used in our approach requires the entire enhancer to be located on the eccDNA, while the “partial” strategy of existing methods only requires partial overlap between the enhancer and the eccDNA [52,53]. For each identified enhancer, their gene targets were ranked by the score for gene-enhancer interaction (S_GE_) calculated based on multiple methods [42]. The top1 gene with the highest S_GE_ for each enhancer was identified as ERG and included in the following analysis. For eccDNA-encoded gene (EEG) identification, bedtools were applied to identify full transcripts plus 35 bp upstream from the transcription start site on eccDNAs. EEGs and ERGs were collectively refered as EDGs. Original EDGs contains multiple types: protein-coding, pseudogene, lncRNA, microRNA, piRNA, and so on. We used protein-coding genes as general EDGs for analysis. The other EDGs were referred as non-coding EDGs.

### Gene ontology, KEGG and disease/tissue association analysis

The Database for Annotation, Visualization, and Integrated Discovery (DAVID) was used for functional enrichment analysis for EDGs [54]. Enriched terms with a P value < 0.05 were presented. Gene-disease association data was collected from DisGeNET, Genetic Association Database (GAD) and Uniprot [55–57]. Gene-tissue association analysis was based on cancer genome anatomy project (CGAP), GNF human expression atlas 2, Human Protein Atlas (HPA) and NCBI UniGene [58,59]. GO biological process analysis and gene set clustering of Top100 eccDrivers was conducted using VissE method [60]. Adjusted Rank Index (ARI) was used for gene-set similarity measure with the overlap threshold of 0.35 and P-value threshold of 0.05.

### Subtype and stage analysis

9 cancer types (HNSC, LUSC, COAD, STAD, LUAD, GBM, BRCA, KIRC, BLCA) with enough subtype information were used for analysis. The method used is the same as that of Liu et al [61]: Wilcoxon test (subtype number = 2) and ANOVA test (subtype number > 2). Subtypes indicate molecular subtypes, if available, else indicate clustering subtypes.

For stage analysis, pathologic stage data of tumor samples from 21 cancer types (ACC, BLCA, BRCA, CHOL, COAD, ESCA, HNSC, KICH, KIRC, KIRP, LIHC, LUAD, LUSC, MESO, PAAD, READ, SKCM, STAD, TGCT, THCA and UVM). mRNA expression and pathologic stage data were merged by sample barcode. The stage subgroup must have at least 5 samples. The GSVA score among groups were compared through the Wilcoxon test (stage number = 2) and ANOVA test (number of stage groups > 2). Trend analysis was performed by Mann-Kendall Trend Test.

### Survival analysis

For survival analysis, clinical data across 33 cancer types was used. mRNA expression data and clinical survival data was merged by sample barcode. Uncensored data and samples with competing risk of death was left out. We divided tumor samples into high and low expression groups based on median mRNA value. The R package survival was used. For each gene in each cancer, Cox Proportional-Hazards model and logrank test of Kaplan–Meier survival was performed.

### Copy number variation analysis

Raw data (n = 11,495) of CNVs were downloaded from TCGA. The mRNA expression and CNV raw data were merged via the TCGA barcode. The association between paired mRNA expression and paired CNV percent samples was tested based on Spearman correlation. The p-value was adjusted by the FDR. The gene set CNV represents the integrated CNV status of gene set for each sample. A sample is classified into Amp. or Dele. group, only when at least one gene in the input gene set is consistently amplified or deleted in this sample. If all genes in inputted gene set have no CNV in a sample, this sample is classified into the WT group. When genes have inconsistent CNV status, for example, gene A was amplified in sample 1, gene B was deleted in sample 1, then sample 1 will be classified in the Excluded group, which will not be considered in this analysis.

### Single nucleotide variation analysis

SNV data (n = 10,234) were collected across 33 cancer types from the TCGA database. Non-deleterious mutations including the Silent, Intron, IGR, 3’ UTR, 5’ UTR, 3’ Flank and 5’ Flank were filtered out and analyzed individually. The gene set SNV represents the integrated SNV status of gene set for each sample. A sample is classified into the Mutant group only when at least one gene in the input gene set is mutated in the sample. If all genes in inputted gene set have no SNV in a sample, this sample is classified into the WT group. Only samples that have deleterious mutants are included in the Mutant group.

### Methylation analysis

Methylation data were collected from TCGA, and only 14 cancer types (THCA, KIRP, BLCA, LIHC, HNSC, BRCA, LUAD, PRAD, ESCA, KICH, LUSC, KIRC, STAD, COAD) having more than 10 paired of tumor and adjacent non-tumor samples were included in the analysis. Before differential methylation analysis, correlation analysis to filter the methylation sites most negatively correlated with gene expression into this analysis was condcuted. A Student’s T test was performed to define the methylation difference between tumor and normal samples, and the p value was adjusted by the FDR: The association between paired mRNA expression and methylation was tested based on spearman correlation. P-values were adjusted by the FDR.

### Pathway activity analysis

We used the reverse phase protein array (RPPA) data from TCPA database for analysis. Ten famous cancer-related pathways were included [61]. The pathway score and pathway activity score (PAS) were estimated according to the method described by Rehan et al and Ye et al [62,63]. When PAS (Gene A group High)> PAS (Gene A group Low), we consider gene A has an activating effect on a pathway, otherwise it has an inhibitory effect on the pathway.

### Drug sensitivity analysis

The IC50 of 265 small molecules in 860 cell lines from GDSC and 481 small molecules in 1001 cell lines from Genomics of Therapeutics Response Portal (CTRP) with its corresponding mRNA gene expression was obtained. Pearson correlation analysis was performed to get the correlation between gene mRNA expression and drug IC50. P-value was adjusted by FDR. The top 30 ranked drugs by the integrated level of correlation coefficient and FDR were displayed.

### Construction of prognostic CEDG-related signature

We used the LASSO algorithm for the CEDGs significantly correlated with prognosis. In the process of model training, the overfitting was minimized. The regularization of LASSO regression was set as a one-time SE. The Cox proportional hazard regression model was next used. Based on the Cox proportional hazard regression model of CEDGs, each patient got a CEDG score as follows: CEDG score = 0.2768*C11orf24 + 0.0661*MYEOV + 0.0843*ALDH3B1 + 0.1076*UNC93B1 + 0.1479*MRGPRD + 0.2884*PPFIA1 + 0.0353* FGF19 + 0.0235* GAL + 0.0861*ANKRD13D + 0.0272*FGF3 + 0.0658*MRPL21 – 0.1364*MTL5–0.1131* CHKA – 0.0844*TCIRG1 – 0.1363*CCND1 – 0.1111*IGHMBP2 – 0.1099*SAPS3. The median CEDG score as a cut- off value divided tumor samples into the high- risk and low- risk groups.

### Local network construction and EDG ranking using eccDriver

eccDriver is a novel five-step algorithm to identify eccDNA-driven driver genes, which combined the degree centrality based on PPI network to assess EDG importance and weighted semi-local centrality based on DEG-based local network to assess the impact of EDGs on transcriptional changes.

Step1. EDG identification as above described.

Step2. For each EDG, an EDGPPI subnet constructed of EDG and its interacting neighbors was extracted from the reference PPI network (STRING). EDGs not covered in the PPI network were removed. The degree centrality of node *v* in PPI network is denoted by *K (v)*.

Step3. DEGs between tumor and normal tissues were identified using limma with criteria of |Log2FC| > 1 and q-value < 0.01. With a maximum depth of 2, an EDG and DEGs are used to construct a local network. PPI network was used as the reference and the local network include source node (EDG), target nodes (DEGs) and intermediate nodes (nodes between EDG and two-hop DEGs).

Step4. The EDG’s influence in the local network was calculated utilizing the weighted semi-local centrality. The weighted semi-local centrality *C*_*WL*_*(v)* of node *v* is defined as:


$$Q\;(v)=\sum_{u\in \Gamma \;(v)}N\;(u),$$ (1)

$$\alpha =\frac{\sum_{v\in Ge}N\;(v)}{2\sum_{v\in Ge}Q\;(v)},$$ (2)

CWL (v)=N (v)+α Q (v), (3)

where Γ *(v)* is the set of the nearest neighbors of node *v. N*(*v*) and *N*(*u*) represents the number of the nearest DEG neighbors of node *v* and *u*, respectively. For each source node *v*, which denotes the EDGs, *u* is its nearest neighbor (*u* could be a DEG, or not a DEG). We introduced α to distinguish the importance of the nearest and the next nearest neighbors, where *Ge* is the set of the EDGs. The higher the *C*_*WL*_*(v)*, the more the EDG’s impact on DEGs in the local network.

Step5. Based on the degree centrality and weighted semi-local centrality score, we next calculated EDG impact score (EGIS) for each EDG:


EGIS(v= (K (v)+1)·(CWL (v)+1) (4)

And then we rank EDGs by EGIS. EGIS assesses EDG’s general importance in PPI network and impact on DEG-based local network, and the Top 100 EDGs were considered as candidate eccDNA-driven drivers.

### Benchmark comparison with existing methods

We assessed the performance of the EDG identification method, eccDriver algorithm and two existing methods used in Circlebase and eccDNADB in cervical cancer [52,53]. The 748 cancer genes in the Cancer Gene Census (CGC; T1 and 2) were collected as the benchmark driver gene datasets [64]. The two criteria derived using true positive (TP), true negative (TN), false positive (FP) and false negative (FN) including (i) the accuracy=(TP + TN)/(TP + TN + FP + FN), (ii) the precision = TP/(TP + FP) and (iii) the recall = TP/(TP + FN) were used to evaluate the methods.

### Expression validation experiment in cancer cell lines

Normal human oral keratinocytes (HOK), human head and neck squamous cell carcinoma 9 (SCC9), squamous cell carcinoma 25 (SCC25), centre antoine lacassagne-27 (CAL27) were used (ATCC; Manassas, VA, USA). The cell lines were stored in state key laboratory of oral diseases (West China Hospital of Stomatology, Sichuan University). Cellular RNA was isolated with TRIzol Reagent (Invitrogen Corporation, CA, USA). cDNA was obtained with ReverTra Ace® qPCR RT Master Mix (Toyobo). The real-time quantitative polymerase chain reaction (RT-qPCR) was performed using SYBR® green realtime PCR Master Mix (Toyobo). The primer sequences used for qPCR in this study are listed in S1 Table.

### Statistical analysis

Statistical analysis was performed using the R software v4.4. Spearman’s Rho method was used to estimate the correlation between two variables. The Wilcoxon rank sum test was used to compare the mean difference between groups. The R package GSVA was used to generate a GSVA score for integrating the expression level of a gene set for further analysis. The log-rank test and Cox proportional hazards regression was used to for survival models. Kaplan–Meier survival curves were used to estimate prognostic significance. The training and test cohorts were standardized by zero-mean normalization. The time-dependent receiver operating characteristic was applied to test the prognosis performance by AUC. Unless specifically indicated otherwise, p value<0.05 was considered statistically significant.

## Results

### An efficient eccDNA-driven gene identification method

**Fig 1:**
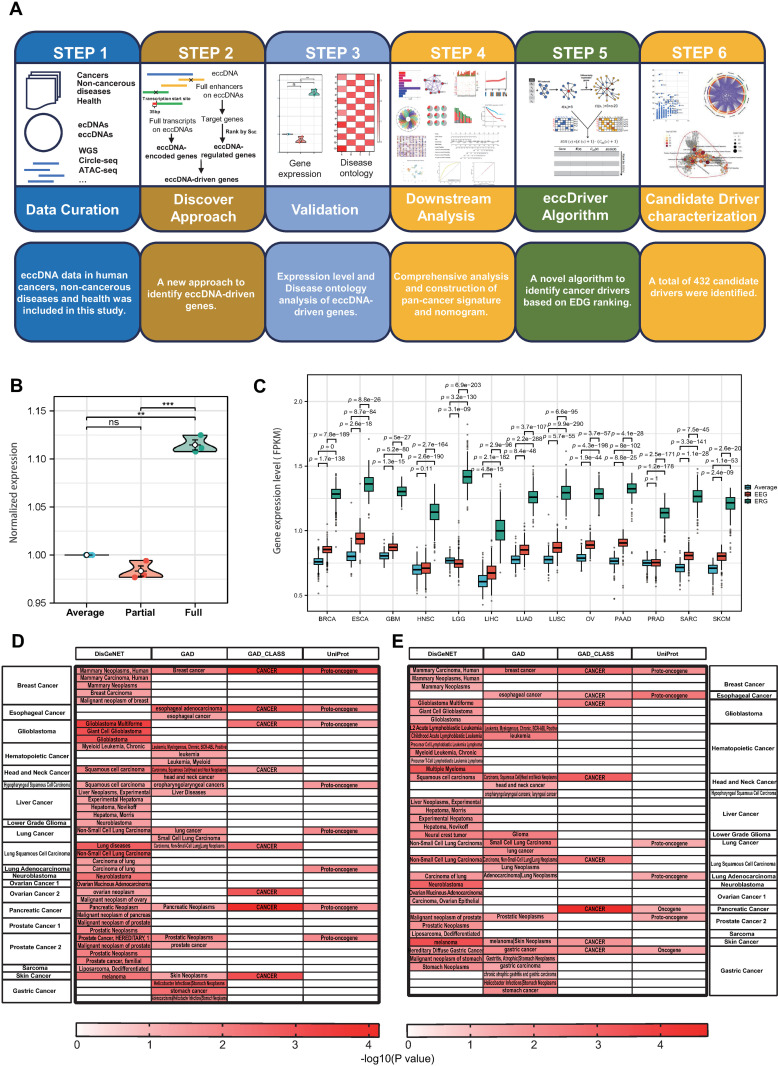
eccDNA-driven gene (EDG) discovery strategy and power. (A) A 6-step method to identify and characterization of EDGs. (B) Comparison of gene expression of ERGs identified by “full” and “partial” strategy. The “full” strategy used in our approach requires the entire enhancer to be located on the eccDNA, while the “partial” strategy of existing methods only requires partial overlap between the enhancer and the eccDNA. (C) Comparison of gene expression of eccDNA-encoded genes (EEGs), eccDNA-regulated genes (ERGs) with the average level. Statistics were calculated with Kruskal-Wallis test and Dunn’s test. (D,E) Disease ontology (DO) analysis to test the enrichment power of ERGs (D) and EEGs (E) to related cancer terms, all P ＜ 0.05.

We take a six-step strategy for EDG catalog and characterization (Fig 1A). eccDNAs were collected from multiple studies [7,15–17,22,40,41]. Totally, eccDNAs of different libraries from 18 types of cancer, 3 types of non-cancerous diseases and 8 types of healthy samples were included in this study. The eccDNA information involved in this study is provided in S2 Table. We leveraged two characteristic properties of eccDNA to enable our computational analysis: (1) eccDNA amplification confers potential for overexpression for genes on the circle; (2) eccDNA lead to the incorporation of new regulatory elements and changes to the topological connections to regulate gene expression. Enhancer data and scores for enhancer-gene interaction (S_GE_) were collected from GeneHancer [42]. Transcript information was collected from Ensembl Genes in table browsers [43]. Existing methods identify both entire and fractions of genes or enhancers on eccDNAs [22,52]. However, these methods are not appropriate for identifying genes mediated by eccDNAs for introducing systematic error for including genes and enhancers without core sequences like promoters or TFBS. Considering our information on these indispensable sequences is not complete, we employed eccDNAs with full transcripts and enhancers on them to minimize uncertainty. Based on puffin model for the transcription initiation activity of human promoter sequences at basepair level, full transcripts plus 35 bp upstream from the transcription start site (TSS) on eccDNAs were identified as eccDNA-encoded genes (EEGs) [25]. For each entire enhancer on eccDNAs, only targets with highest S_GE_ were identified as eccDNA-regulated genes (ERGs) based on the model of enhancer release and retargeting [65]. Collectively, EEGs and ERGs were referred as eccDNA-driven genes (EDGs). Notably, the connotation of EEG, ERG and EDG is weighted in the potential of a gene to be overexpressed as a result of the intrinsic features of eccDNA, but the actual gene expression is affected by many other factors such as its epigenetic states. To test this potential, we investigated the expression level of EDGs. The “full” strategy used in our approach requires the entire enhancer to be located on the eccDNA, while the “partial” strategy of existing methods only requires partial overlap between the enhancer and the eccDNA [52,53]. The “full” strategy requires longer eccDNAs, and those short eccDNAs which don’t meet the inclusion criteria of the full approach were filtered out, which contribute to the differences observed between the “full” and “partial” strategy. The eccDNAs applied to the full strategy to identify EDGs are longer in length compared to the background of all eccDNAs (S1A Fig). We compared the expression of ERGs identified by either our “full” or existing “partial” strategy in HeLa cells (Fig 1B). The results showed ERG expression was above average in both groups, whereas ERGs in “full” group presented higher expression than those in “partial” group, demonstrating the superiority of our approach over existing methods. We further incorporated recent HeLa cell datasets containing eccDNA data for the comparison [41]. Similarly, these new data showed the expression levels of the ERGs identified by “full” strategy was higher than those by “partial” strategy (S1B Fig). Next, we explored the expression of EEGs and ERGs in different TCGA tumors (Fig 1C). Both EEGs and ERGs exhibit higher expression than the average level, showing the validity of our approach and assumptions. To investigate the dominant functional mode of eccDNA in tumors, we also compared the expression levels of EEGs and ERGs. We found that ERGs are significantly more highly expressed than EEGs (Fig 1C). This suggests that eccDNA may function more strongly as an enhancer than as a transcriptional template in tumors, providing new insights into the biological roles of eccDNA. The overexpression of EEGs is modest compared to the average level, in line with our theory and former studies [66]. To further test the power of the method, we performed disease ontology (DO) analysis for EDGs in DisGeNET, GAD and Uniprot [55–57]. Identified EDGs were enriched to correlated diseases, confirming the accuracy and power of this method to identify key disease-related genes (Fig 1D and E).

### Functional enrichment of EDGs in cancer

To explore the relevant biological functions and pathways of EDGs in cancer, we performed functional enrichment analysis for different cancer types. Gene ontology (GO) analysis revealed that ERGs in different cancer types shared common terms like “regulation of transcription from RNA polymerase II promoter”, “regulation of transcription, DNA−templated”, “potassium ion transmembrane transport”, “peptide antigen assembly with MHC class II protein complex”, “intracellular signal transduction” and “adaptive immune response” (S1C Fig). Kyoto Encyclopedia of Genes and Genomes (KEGG) analysis revealed that ERGs in different cancers shared common terms like “Human papillomavirus infection”, “PI3K−Akt signaling pathway”, “Pathways in cancer” and “Proteoglycans in cancer” (S1D Fig). GO enrichment analysis for EEGs in cancer revealed common terms like “protein localization to CENP−A containing chromatin”, “regulation of transcription, DNA−templated” and “telomere organization” (S2A Fig). KEGG enrichment analysis for EEGs in cancer revealed common terms like “Herpes simplex virus 1 infection”, “Alcoholism” and “Viral carcinogenesis” (S2B Fig). These findings revealed general functions of EDGs that and further support the validity of the discovery method. Of note, the results demonstrated the biological processes and pathways involved in not only cancer development but also basic cell life activity. To further investigate the roles of EDGs in cancer development specifically, we intersect EDGs with upregulated genes and obtained upregulated EDGs in lung adenocarcinoma (LUAD) (S3 Table). Protein-protein interaction (PPI) network based on upregulated EDGs was constructed by STRING (S3A Fig) [67]. GO enrichment analysis revealed that upregulated EDGs were enriched in terms like “ephrin receptor signaling pathway” and “cell division” (S3B Fig). KEGG enrichment analysis revealed terms relevant to cancers like “MicroRNAs in cancer”, “Pathways in cancer”, “Small cell lung cancer” and “PI3K−Akt signaling pathway” (S3C Fig). The top 10 hub genes were screened out with MCC as the criterion using Cytoscape (S3D Fig) [68]. Survival analysis showed that high expression of all the 10 hub genes was related to poor prognosis in LUAD patients (S3E–G Fig), demonstrating their importance in LUAD progression and clinical potential.

### Expression and survival analysis of common EDGs in cancer

Notably, we found most cancers share 306 enhancer regions and 175 transcripts on eccDNAs (S1E Fig), indicating their key roles in cancer (S4 Table). All of these sequences come from q13 region of chromosome 11, one of the most prevalent amplified regions in cancer. Totally, 27 common EDGs (CEDGs) were obtained from these regions, including famous oncogene CCND1 which was found amplified on eccDNA [2]. Differential expression analysis demonstrated the aberrant expression of CEDGs in cancer (S1F Fig). Gene Set Enrichment Analysis (GSVA) was conducted using GSCA to estimate the integrated expression level of CEDGs [61]. GSVA scores are significantly higher in tumor compared to normal samples for bladder urothelial carcinoma (BLCA), breast invasive carcinoma (BRCA), colon adenocarcinoma (COAD), esophageal carcinoma (ESCA), head and neck squamous cell carcinoma (HNSC), kidney chromophobe (KICH), kidney renal clear cell carcinoma (KIRC), kidney renal papillary cell carcinoma (KIRP), LUAD, stomach adenocarcinoma (STAD) and thyroid carcinoma (THCA) (S1G Fig). We next estimated the expression difference of CEDGs between cancer subtypes. 9 cancer types (BLCA, BRCA, COAD, glioblastoma multiforme [GBM], HNSC, KIRC, LUAD, lung squamous cell carcinoma [LUSC] and STAD) with subtype information were included in the analysis. We found that most of the CEDGs were significantly differential expressed among subtypes in KIRC, LUAD, STAD, BRCA and GBM (S4A Fig). For example, MRGPRF expression is higher in HNSC-negative compared to HNSC-positive (S4B Fig). In the four subtypes of LUSC, LUSC-secretory group exhibits the highest MRGPRF expression while the lowest MRGPRF expression in LUSC-primitive group (S4C Fig). GSVA scores showed significant differences in BRCA, GBM, HNSC, KIRC, LUAD, LUSC and STAD (S4D Fig). GSVA scores in HNSC-negative are higher than that of HNSC-positive and there are highest GSVA scores in BRCA-LumB among the 5 BRCA subtypes. We also estimated the change of CEDG expression and GSVA scores among pathological stages (from stage I to IV) in cancer. Significant correlation was observed in different cancers (S4E Fig). Most genes presented a rising tendency in different cancer types (S4F Fig), like MRGPRF in BLCA (S4G Fig). However, a few genes demonstrated a downward trend, like KMT5B in THCA (S4H Fig). GSVA scores of the genes exhibit significant variability across different stages in most cancers (S4I and J Fig). Specifically, notable cancers include BLCA, BRCA, LUSC, SKCM, THCA, and UVM. A rising tendency is exhibited in BLCA, BRCA, COAD, LUSC, STAD, THCA and UVM. However, the scores show a decreasing trend in ACC. Additionally, the results highlight a consistent downward trend in SKCM, alongside a similar pattern in TGCT. These results reflect the stage dependency and functional heterogeneity of the CEDGs in different tumors. Next, the survival (OS, PFS, DSS, and DFI) difference between high and low gene expression groups of CEDGs was explored in TCGA cancers. As a result, we found high expression of most CEDGs was correlated to poor OS, PFS, DSS and DFI in different cancers (S4K Fig). The Kaplan-Meier (KM) survival curve of ANO1 in KIRP demonstrated a correlation of high ANO1 expression and poor OS (P = 0.00039), PFS (P = 0.0021), DSS (P = 3.6e-06), and DFI (P = 0.0033) (S4L–O Fig). Additionally, we identified 18 common non-coding EDGs in cancer. Differential expression analysis demonstrated that several non-coding EDGs were down-regulated in cancer (S5A Fig). Survival analysis demonstrated that high expression of RP11 − 802E16.3 was correlated to poor OS of KIRC patients (S5B and C Fig). These results collectively indicate the key roles of CEDGs in cancer progression and their potential clinical value in pan-cancer prognosis prediction.

### Copy number variation of CEDGs

**Fig 2:**
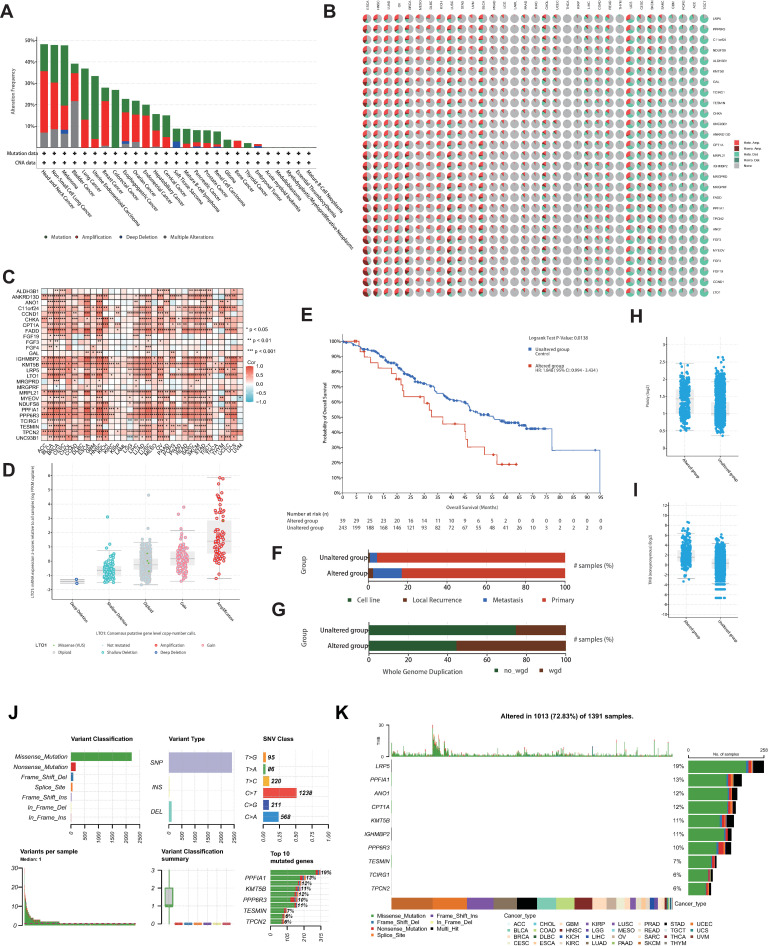
Genetic alteration feature of the dysregulated CEDGs in cancer. **(A)** The alteration frequency of CEDGs in cancer using cbioportal. **(B)** Pie chart showing copy number variation (CNV) distribution in TCGA tumors. **(C)** Heatmap showing the correlation of CNV and gene expression. **(D)** LTO1 expression in different alteration classes. **(E)** KM plot showing altered group demonstrating poor prognosis compared with unaltered group. **(F)** Sample types distribution showing higher proportion of local recurrence and metastasis in altered group. **(G)** Sample types distribution showing higher proportion of WGD in altered group. Percentage of WGD indicates the proportion of tumors that have undergone WGD, the complete replication of entire genome in a single event. (H, **I)** Comparison of tumor ploidy (H) and TMB (I) in altered and unaltered group. Tumor ploidy refers to the chromosome set number in tumor cells. TMB represents the number of mutations per megabase in the tumor genome. **(J)** Overall Single nucleotide variation (SNV) landscape. **(K)** Oncoplot demonsrtating the SNV distribution of the top 10 altered CEDGs.

eccDNA contributes to genomic amplification and increased oncogene expression [1]. As eccDNA has been recognized to increase the gene copy number, we explored the landscape of copy number variation (CNV) of CEDGs [7,66]. First, we investigated the genetic alteration status of CEDGs in different cancers by cbioportal [69]. As we have hypothesized, amplification was the dominating type in most cancer types like head and neck, non-small cell lung, bladder, breast, esophagogastric, ovarian, endometrial, hepatobiliary and bone cancer (Fig 2A). The highest alteration frequency of CEDGs was shown in head and neck cancer, mostly amplification. Amplification of the 27 CEDGs presents similar pattern of distribution, indicating the fundamentally similar driver factors (S6 Fig). Based on TCGA cancers, the CNV pie chart distribution show that the main CNV types are amplification in ESCA, HNSC, LUAD, ovarian serous cystadenocarcinoma (OV), BRCA, mesothelioma (MESO), diffuse large B cell lymphoma (DLBC), KICH, LUSC, STAD, uveal melanoma (UVM), BLCA, prostate adenocarcinoma (PRAD), brain lower grade glioma (LGG), acute myeloid leukemia (LAML), pancreatic adenocarcinoma (PAAD), kidney renal clear cell carcinoma (KIRC) and cholangiocarcinoma (CHOL), while deletion is prevalent in cervical squamous cell carcinoma and endocervical adenocarcinoma (CESC), skin cutaneous melanoma (SKCM), sarcoma (SARC), GBM, pheochromocytoma and paraganglioma (PCPG), adrenocortical carcinoma (ACC) and testicular germ cell tumors (TGCT) (Fig 2B). Intriguingly, deletion of all CEDGs in TGCT was greater than 75%, consistent with former study that eccDNA formation and distribution in sperm and somatic cells is fundamentally different [70]. CNV percentage analysis demonstrated heterozygous amplification/deletion and homozygous amplification/deletion of CEDGs (S7A and B Fig). Correlation analysis indicated that almost all mRNA expression was positively correlated with CNV (Fig 2C). Compared with other groups, amplification group exhibited higher mRNA expression for LTO1 (Fig 2D) and other CEDGs (S8 Fig). Survival analysis revealed significance of CNV in patient DFI, DSS, OS and PFS (S7C Fig). These results indicated that the CNV of CEDGs contributed to their abnormal expression, which might play an important role in cancer progression. Based on genetic alteration from cbioportal, samples were divided into altered and unaltered group. The Survival analysis demonstrated that altered group exhibited poor prognosis (HR = 1.848, P = 0.0138) (Fig 2E). For sample types, altered group exhibited higher rates of local recurrence and metastasis, indicating CEDG alteration promotes cancer metastasis and leads to cancer recurrence (P ＜ 0.0001, Fig 2F). The tumor ploidy, Tumor Mutation Burden (TMB), and the percentage of whole genome duplication (WGD) in altered group are higher compared with unaltered group (All P ＜ 0.0001, Fig 2G–I). Tumor ploidy refers to the chromosome set number in tumor cells, often associated with genomic instability [71]. TMB represents the number of mutations per megabase in the tumor genome and is an important biomarker for immunotherapy response [72]. Percentage of WGD indicates the proportion of tumors that have undergone WGD, the complete replication of entire genome in a single event, which is associated with tumor progression and prognosis [73]. Increased tumor ploidy and percentage of WGD indicate more significant genomic alterations in the altered group, suggesting higher genomic instability and more malignant behavior. Elevated TMB suggests that tumors in the altered group may have a higher mutation burden and could be more responsive to immunotherapy. These findings imply that tumors in the altered group may be more aggressive but also potentially more sensitive to immunotherapy.

### Single nucleotide variation of CEDGs

We also analyzed CEDG-related single nucleotide variation (SNV) to detect the frequency and variant types in cancer. SNV frequency is 41 in uterine corpus endometrial carcinoma (UCEC) and 42 in SKCM for LRP5, and 44 for PPFIA1 in UCEC (S9A Fig). For variant classification, missense mutations were the main type (Fig 2J). SNV alteration was found in 72.83% of the samples and the top 10 mutated genes were LRP5, PPFIA1, ANO1, CPT1A, KMT5B, IGHMBP2, PPP6R3, TESMIN, TCIRG1, and TPCN2, of which the mutation percentages were 19%, 13%, 12%, 12%, 11%, 11%, 10%, 7%, 6%, and 6%, respectively (Fig 2K). Mutation spectrum of transition (Ti) and transversion (Tv) categories was shown in S9B Fig. SNV survival analysis revealed that several SNVs were significantly associated with patient prognosis (S9C Fig). We also investigated the non-deleterious mutations. SNV frequency of non-deleterious mutations is 32 for KMT5B and 31 for ANO1 in UCEC, and 31 for LRP5 in SKCM (S10 Fig and S5 Table).

### Methylation analysis of CEDGs

To get more profound knowledge of CEDG expression, we explored their epigenetic states by methylation analysis. Most of CEDGs like CCND1, ALDH3B1, MYEOV were frequently hypomethylated in cancer, while FGF3, FGF4, MRGPRF and CPT1A were hypermethylated in most cancers (S11A Fig). Methylation and mRNA expression correlation analysis indicated that the expression levels of CEDGs correlated negatively with their methylation level, and only ANO1 in liver cancer (LIHC), BLCA, ESCA, SKCM, HNSC, LUSC, UVM, PAAD, LUAD, CESC, KIRP and COAD; GAL in LUSC, SARC, LUAD and PRAD; LRP5 in TGCT and PRAD; and MRGPRD in SARC showed a positive correlation between methylation and gene expression (S11B Fig). Survival analysis demonstrated heterogeneity across cancer types. In cancers like LGG, THYM, HNSC and PAAD, the lower methylation was mainly associated with poor survival, while in UCEC, KIRP, UVM and ACC, lower methylation was mainly associated with better survival (S11C Fig). The results of methylation analysis further expanded our understanding of CEDGs expression, explaining the decreased expression of some CEDG in a certain cancer type, like ALDH3B1 in LUSC and MRGPRF in UCEC.

### Pathway activity and single-cell state analysis of CEDGs

**Fig 3:**
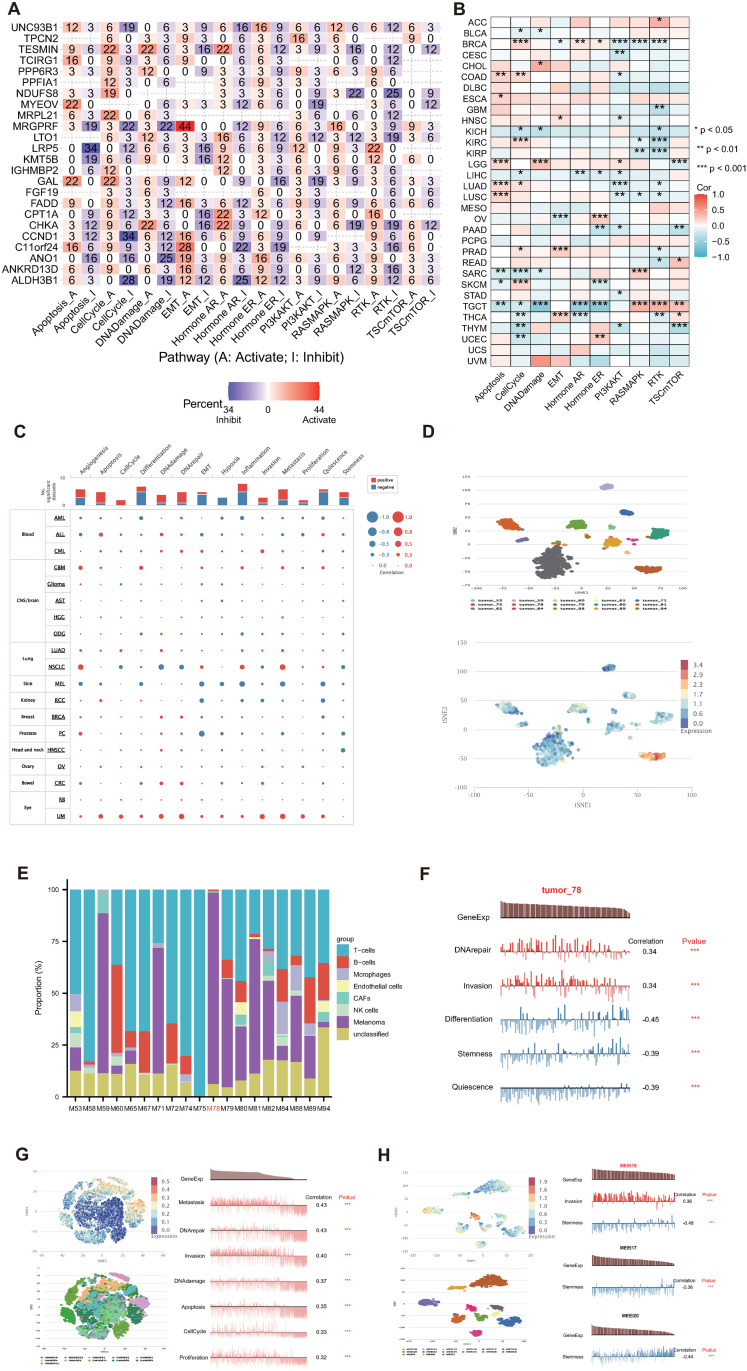
Pathway activity and single-cell level functional states of CEDGs in cancer. **(A)** The percentage of the effect of CEDGs on cancer-related pathways. Zero values indicate a one-way effect of a gene on a pathway. Missing values indicate no significant relationship between a gene and a pathway. **(B)** Correlation of GSVA core and pathway activity in TCGA tumors. **(C)** Average correlations between CEDGs and functional states in different cancers from CancerSEA. **(D)** Expression distribution of CEDGs in distinct cell groups in MEL. **(E)** Cell distribution of CEDGs in distinct cell groups in MEL. **(F)** Functional relevance of CEDGs in MEL. **(G, H)** Expression distribution of CEDGs in distinct cell groups and functional relevance in UM (G) and HNSCC **(H)**. The cell groupings are predefined with t-SNE plots of gene expression and the cell groups denoted different patients, following classification criteria established in the published literature by CancerSEA.

To further reveal the mechanisms underlying CEDGs in cancer development, we investigate their roles in cancer-related pathways. Related results indicated that the 27 CEDGs were significantly involved in famous cancer-related pathways, including apoptosis, cell cycle, DNA damage, EMT, hormone AR, hormone ER, PI3K/AKT, RTK, RAS/MAPK and TSC/mTOR pathways (Fig 3A). For example, TESMIN, PPFIA1, NDUFS8, MRPL21, IGHMBP2 and GAL were involved in cell cycle activation (22%, 12%, 19%, 22%, 12% and 22%, respectively). MRGPRF was significantly involved in the activation of EMT (44% activation vs. 0% inhibition) while LRP5 was significantly involved in the inhibition of apoptosis (34% inhibition vs. 0% activation) (All P < 0.05, Fig 3A). Example box plots showed the Cell cycle and EMT scores in high and low expression group of MRPL21 and MRGPRF in BRCA, LUAD and STAD (S12A–F Fig). GSVA scores are significantly related to the 10 pathways and showed obvious differences across different cancer types (Fig 3B). For example, GSVA scores were positively correlated with apoptosis activity in COAD, ESCA, LGG, LUAD and LUSC while negatively in SARC, SKCM and TGCT. The findings suggested that CEDGs play a key role in regulating cancer-related pathways. For a more comprehensive understanding, the relationship between CEDGs and functional states in different cancer types was explored at single-cell resolution by CancerSEA [74]. We observed that CEDGs were positively correlated with apoptosis, cell cycle, DNA damage, DNA repair, invasion and metastasis while negatively related to states like differentiation, hypoxia and quiescence (Fig 3C). In skin melanoma (MEL), Expression of CEDGs in tumor_78 is distinctively higher than the other cell groups (Fig 3D). The cell groupings are predefined with t-SNE plots of gene expression and the cell groups denoted different patients, following classification criteria established in the published literature by CancerSEA. Tumor_78 contains the highest portion of malignant cells based on inferred CNV patterns and is designated as high-cycling tumor for cell cycle (nearly 30% fraction of cycling cells compared to 13.5% on average) (Fig 3E) [75]. Tumor_59 and Tumor_71 exhibit high malignant cell proportions but are low-cycling tumors, with cycling cell fractions of 7%-9% and 1%-3%, respectively, compared to nearly 30% for Tumor_78 and the average of 13.5% [75]. The CEDG expression is positively correlated with DNA repair and invasion, negatively with differentiation, stemness and quiescence in tumor_78 but similar associations is absent in other cell groups (All P ＜ 0.001, Fig 3F). In uveal melanoma (UM), despite the complex cell composition within individual tumors, class 2 tumors such as UMM061, UMM063 and UMM064 are associated with high metastatic risk and inactivating mutations in BAP1 [76]. The CEDG expression is positively related to metastasis, invasion, DNA demage/repair, apoptosis, cell cycle and proliferation and was higher in Class 2 primary tumors like UMM61 and UMM63 compared to others (All P ＜ 0.001, Fig 3G) [76]. In HNSCC, CEDG expression is distinctively higher in MEEI18 than the others. In MEEI18, CEDG expression is positively correlated with invasion and negatively related to stemness, while only negative correlation between CEDG expression and stemness is observed in other groups like MEEI17 and MEEI20 (Fig 3H) [77]. These results demonstrated that CEDGs were involved in pathways related to cancer progression and discriminating malignant cell groups with higher level of cell cycle, invasion and metastasis.

### Immune infiltration analysis of CEDGs

Tumor-infiltrating immune cells play key roles in the tumor formation and development [78–80]. We next explored the correlation of CEDG expression and the infiltration level of different immune cells in TCGA tumors. GSVA scores of CEDGs were positively correlated with infiltration score and cell types of NK, NKT, monocyte, cytotoxic, Th2, macrophage, exhausted, CD8_T, effector_memory, gamma_delta and Th1, while negatively related to cell types of central_memory, th17, iTreg, CD4_naive, CD8_naive, Bcell and Tr1 in most cancers (S13A Fig). Correlation of immune infiltration with epression and methylation of CEDGs showed the complexity (S13B–E Fig). Intriguingly, significant correlation of the immune infiltration level and CNV of all CEDGs. CEDG CNV were positively correlated with monocyte, neutrophil, NKT and Th17, while negatively related to cell types of central_memory, exhausted, Bcell, CD8_T, cytotoxic, gamma_delta and Tfh in HNSC (S13F Fig). In THYM, CEDG CNV were positively correlated with infiltration score and cell types of macrophage, monocyte, neutrophil, NK, NKT, nTreg and Th2, while negatively related to cell types of CD4_naive, CD8_naive, CD4_T, CD8_T and Tfh (S13G Fig). Gene set CNV and SNV were significantly correlated with different immune cell infiltration (S14A and B Fig). CEDG expression is significantly associated with TMB (S13H Fig). For example, MYEOV expression is positively correlated with TMB in most cancers (S13I Fig). These results indicated the immune related mechanisms of CEDGs promoting cancer development and the potential of CEDGs for cancer immunotherapy.

### Microbiome correlation of CEDGs

Accumulating evidence suggests that the microbiota has roles in cancer development and patient prognosis [50,81]. Herein, we explored the relationship of CEDGs and the tissue-resident microbiota abundance for all gastrointestinal cancers in TCGA (HNSC, ESCA, STAD, COAD and rectum adenocarcinoma [READ]) based on The Cancer Microbiome Atlas (TCMA) [50]. We found the expression of CEDGs was significantly related to cancer microbiota abundance (S15A Fig). Two distinctive relation mode of CEDGs and microbiota was observed (S15B Fig). Expression of most CEDGs like TESMIN and LRP5 was positively with genus like bacteroides, capnocytophaga, parabacteroides and alistipes, while negatively with genus like prevotella and capnocytophaga. However, several CEDGs like KMT5B and ANO1 were quite the opposite.

### Establishment of CEDG-related signature and nomogram for pan-cancer prognosis

**Fig 4:**
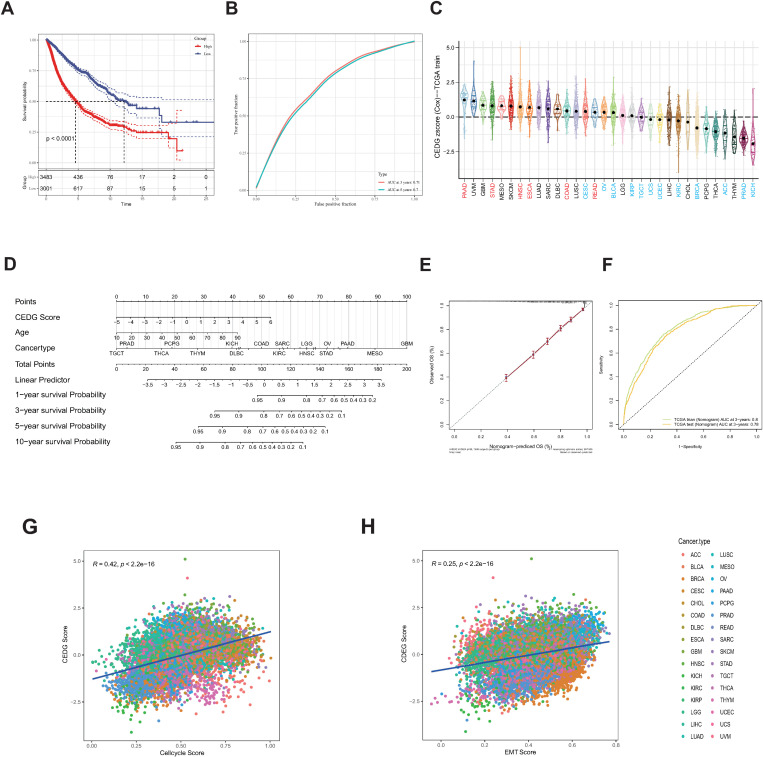
A pan-cancer signature and nomogram based on CEDGs. **(A, B)** Prognostic performance of the 17-gene CEDG score. **(C)** CEDG score in different TCGA cancers. Red names represent tumors from the gastrointestinal tract, and blue names represent tumors of organs related to the urinary and reproductive system. **(D)** Nomogram derived from the CEDG signature. **(E)** The 3-year calibration curve. **(F)** The AUC of nomogram prediction for 3 years. **(G, H)** Correlation analysis showing significant relationship of CEDG score with cell cycle (G) and EMT (H) features of cancers.

Next, we constructed a CEDG signature for the pan-cancer datasets using the Least Absolute Shrinkage and Selection Operator (LASSO) Cox model. TCGA samples were randomly divided into a 70% training set and a 30% testing set randomly. First, we performed univariate cox regression analysis for the 27 CEDGs in the TCGA-training cohort (S16A Fig). Based on the criterion of p ＜ 0.01, 21 CEDGs significantly related to patient prognosis were obtained. After LASSO regression analysis of the 21 CEDGs in the training cohort (S16B Fig), we got 19 CEDGs with non-zero coefficients. Finally, multivariate cox regression analysis and stepwise regression was applied to the 19 CEDGs to construct the prognostic model and a 17-gene CEDG score was obtained. The distribution of the CEDG scores is showed in S16C Fig. Based on the median CEDG score, we divided the patients into high-risk and low-risk groups. Survival analysis showed that the higher CEDG score was associated with the worse OS in TCGA-training cohort (Fig 4A). The area under the curve (AUC) of the CEDG signature in prognosis prediction is 0.71 at 3 years and 0.7 at 5 years (Fig 4B). Next, we validated the prognostic power of the CEDG score in the TCGA-testing set (S16D and E Fig). Survival analysis in the test set demonstrated that high CEDG score was correlated with poor OS, in line with the train cohort. The CEDG scores in tumors from the gastro-intestinal tract had greater CEDG scores, such as PAAD and STAD, while the tumors of organs related to the urinary and reproductive system, for example, BRCA, ACC, PRAD and KICH, usually had lower ones (Fig 4C). For a readable and quantitative measurement for the CEDG signature in clinical application, a comprehensive nomogram combing the CEDG score and clinical factors (age and cancer type) was established (Fig 4D). The calibration curve of the 3-year OS for the nomogram was nearly coincided with the standard curve, illustrating that prediction by the nomogram was close to the reality (Fig 4E). The AUC of 3 prediction by nomogram was 0.8 (3-year), 0.8 (5-year) in the training cohort and 0.78 (3-year), 0.79 (5-year) in the test cohort (Figs 4F and S16F). This model has fewer parameters and better performance compared with former pan-cancer models constructed by similar or different methodology [19,82]. To investigate the connection between the CEDG score and malignant features of EMT and cell cycle, we quantified the EMT and cell cycle of tumor based on published gene sets [83,84]. CEDG score was found positively correlated with cell cycle score (R = 0.42, p < 2.2e-16) and EMT score (R = 0.25, p < 2.2e-16) in the TCGA pan-cancer set (Fig 4G and H) and in most of the cancer types (S16G and H Fig). In other words, the tumor with high CEDG score was generally accompanied by more aggressive tumor cells, consistent with the results in pathway and single-state analysis.

### Drug sensitivity analysis of CEDGs

To explore the roles of CEDGs in chemotherapy and targeted therapy, drug sensitivity analysis of CEDGs was performed in different cancer cell lines based on the GDSC and CTRP [48,49]. Results showed that the expression of MRGPRD, ANKRD and IGHMBP2 negatively correlated with IC50 of multiple drugs like panobinostat, etoposide, FK866, methotrexate and I − BET − 762, demonstrating drug sensitivity. However, more CEDGs like NDUFS8, PPFIA1, LRP5, CCND1, C11orf24, ALDH3B1, FADD, MYEOV and ANO1 resulted in drug resistance toward different kinds of drugs (S17 Fig). These results indicated that the CEDGs might mediate drug sensitivity and be potential targets for cancer treatment.

### eccDriver algorithm identifies candidate eccDNA-driven cancer drivers

**Fig 5:**
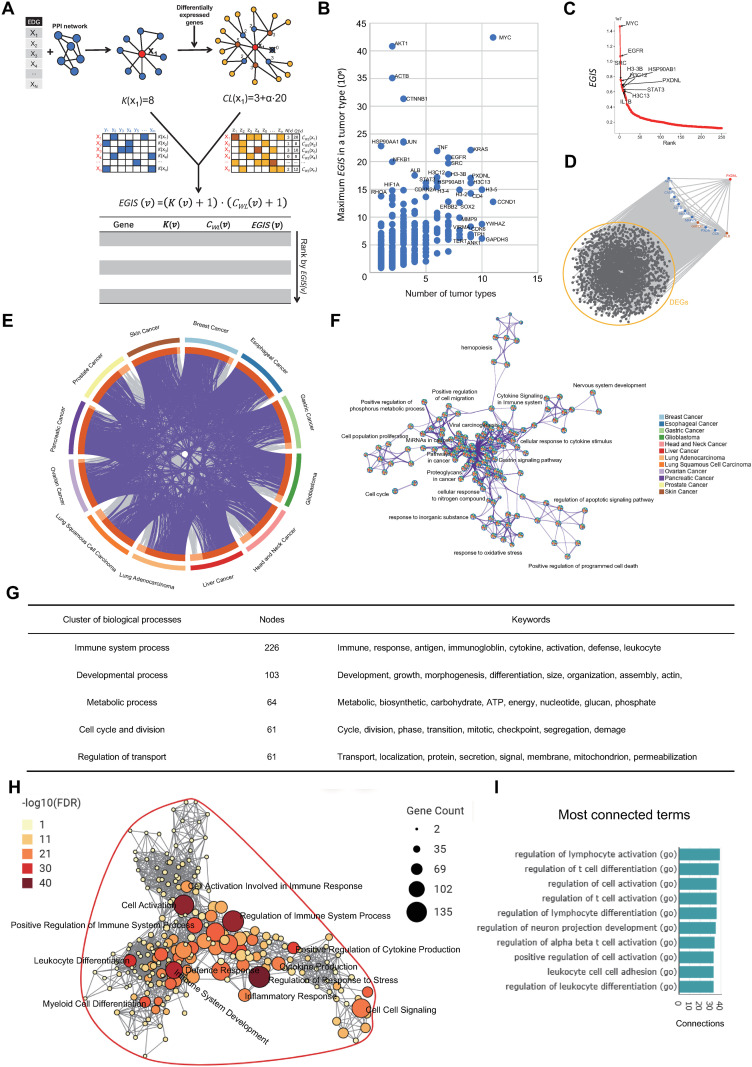
eccDriver identifies eccDNA-driven drivers in different cancer types. (A) Workflow of eccDriver. In a local network, red-source node, blue-intermediate node, yellow-target node, brown-both intermediate and target node. (B) Distribution of candidate drivers across different cancer types. (C) EDG ranking in breast cancer. Genes labeled are the top 10 drivers. (D) Local subnetwork of PXDNL in breast cancer. Only ten nearest neighbors with the highest interaction scores are demonstrated, in which GAPDH and ALB are differentially expressed genes (DEGs). (E) Common drivers in different cancer types at the level of gene ID (purple curves) or ontology term enrichment (grey curves). (F) Metascape network showing clusters of enriched terms from the candidate drivers. Edges denote the similarity and node colors denote the contribution of cancer types. (G) The five broad biological process categories. Nodes denote the biological process terms. (H) Network of clustered biological processes in immune system process. (I) The most connected terms were clustered into immune system process.

To identify eccDNA-driven cancer drivers, we developed eccDriver, a network-based algorithm, to rank EDGs by importance and influence. eccDriver is a novel algorithm analyzing population-based interrogations of eccDNA and transcriptomic profile for identification of eccDNA-driven drivers in cancer. We combined the degree centrality based on PPI network to assess EDG importance and weighted semi-local centrality based on DEG-based local network to assess the impact of EDGs on transcriptional changes. The original local centrality consults the information of the four-hop neighbors and equals the importance of the nodes of different layers. We made improvement from following two aspects: firstly, we introduced a weighting coefficient α for node importance, which increased the accuracy; secondly, considering the decreasing of the node importance as the distance increases, we only utilized the information of neighbors within two hops of the source node, which simplified the local network and ulteriorly decreased the computational complexity. eccDriver has a novel scheme (Fig 5A): firstly, we calculate the degree of each EDG in the reference PPI network, which represents its general importance; then, we identify a local network with the depth of two for each EDG, and assess the impact of EDG using the weighted semi-local centrality; finally, EDGs are ranked by the EDG impact score (EGIS), a score calculated according to the degree and weighted semi-local centrality, and the Top 100 EDGs are considered as candidate eccDNA-driven drivers.

We applied eccDriver algorithm in 12 cancer types, including Breast Cancer, Esophageal Cancer, Glioblastoma, Head and Neck Cancer, Liver Cancer, Lung Squamous Cell Carcinoma, Lung Adenocarcinoma, Ovarian Cancer, Pancreatic Cancer, Prostate Cancer, Skin Cancer and Gastric Cancer. The Top 100 EDGs were considered as the candidate eccDNA-driven drivers. In total, 432 candidate drivers were identified, among which 266 genes were found in more than two cancer types and 55 genes were identified in more than six cancer types (Fig 5B and S6 Table). We utilized driver genes in CGC as known drivers. Results included known cancer driver genes on eccDNA were identified, such as MYC, EGFR, CCND1 and MDM2. MYC, a well-known eccDNA-amplified driver gene which promotes cancer cell proliferation, immortalization and cross-resistance, was identified in the most cancer types (11 of 12) with the highest EGIS, followed by CCND1 (11 of 12), H3-5 (10 of 12), YWHAZ (10 of 12) and GAPDHS (10 of 12) [85,86]. ERBB2, a known cancer driver which could hijacks MYC’s enhancers by forming a MYC-ERBB2 chimeric ecDNA to elevate the expression level, was also identified [87]. Several candidate drivers are known but have not been reported regulated by eccDNA, such as SRC, STAT3 and SOX2. Meanwhile, we also identified novel cancer drivers, such as PXDNL, YWHAZ and MMP9. While many genes get high EGIS in specific cancer types, such as HSP90AA1 in pancreatic cancer, AKT1 in glioblastoma and pancreatic cancer, and ACTB in glioblastoma and liver cancer. In breast cancer, the Top 5 genes were MYC, EGFR, SRC, HSP90AB1, PXDNL, and the Top 4 genes are CGC drivers (Fig 5C). PXDNL promotes cell motility of MCF-7 cells via the Wnt/β-catenin pathway [88]. Although not be a CGC driver, PXDNL was found with the mutation prevalence of 6.1% in 6697 patients of breast cancer and related to breast cancer immune infiltration and poor prognosis [89–92]. The subnet (top 10 of PXDNL’s neighbors based on interaction scores) of the local network of novel driver PXDNL was demonstrated (Fig 5D). Next, we investigated the prognostic significance of the highest-scoring eccDNA-driven drivers identified in HNSC, LIHC, GBM, OV, PAAD and PRAD. The highest-scoring eccDNA-driven driver is ACTB in GBM and LIHC, EGFR in HNSC, and MYC in PAAD, OV and PRAD. We found high expression of these eccDNA-driven drivers correlated to poor OS in related cancers (S18 Fig). We further explored the expression levels of the four genes (EGFR, KRAS, CDKN2A and H3C13) which scored the highest among the eccDNA-driven drivers in HNSC. The results showed that the expression levels of EGFR, KRAS, CDKN2A and H3C13 were up-regulated in HNSC (S19A–D Fig). Further experimental data validated their elevated expression in different HNSC cell lines (S19E–H Fig). Additionally, we assessed the performance of our methods and two existing alternatives in cervical cancer. The results demonstrate that compared with existing methods, our EDG method demonstrates improvements in both accuracy and precision (Table 1). By applying our eccDriver algorithm to prioritize the EDGs, we achieved further enhancements in accuracy and precision. By setting a threshold at the top 100 candidates, we prioritized key EDGs and excluded EDGs of lower importance. This selection effectively minimizes noise from low-confidence EDGs, which contribute to FP. However, our methods reduced the recall. When selecting the top 200 candidate genes using eccDriver, the precision drops from 15% to 11.5%, while the recall rises from 2.01% to 3.07%

**Table 1:** The comparison of our methods with existing methods.

Methods	Accuracy	Precision	Recall
eccDNADB	80.49%	3.40%	53.88%
CircleBase	78.68%	3.43%	59.76%
Our EDG	97.68%	3.61%	3.61%
Our eccDriver-100	98.65%	15.00%	2.01%
Our eccDriver-200	98.51%	11.50%	3.07%

### eccDNA-driven drivers regulate key biological processes

We found different cancer types shared a large portion of the candidate drivers and there was considerable overlap in biological pathways (Fig 5E), including positive regulation of cell migration, cell cycle, cell population proliferation, cytokine signaling in immune system (Fig 5F). Our parallel analyses emphasize the overlap of candidate drivers and pathways among different cancer types.

The redundancy of the enriched terms makes the results not easily interpretable biologically, we thus performed further analysis by vissE to identify specific directionality of these genes on the biological processe [60]. The gene-set similarity was computed using the Adjusted Rand Index (ARI). By inspection of the largest gene set clusters, the biological processes enriched by the candidate drivers were clustered into five broad categories. The five biological themes were assigned a name according to the constituent GO terms and keywords. The five clusters included immune system process, developmental process, metabolic process, cell cycle and division, and regulation of transport (Fig 5G). The most connected biological terms belong to immune system processes (Fig 5H and I). The largest term in immune system processes is regulation of immune system process (135 genes) (Fig 5H), and cell cycle in cell cycle and division (131 genes), and organophosphate metabolic process (54 genes) in metabolic process (S20A and B Fig).

In brief, the findings emphasize overlap genes and pathways among cancer types, and suggest the five broad biological themes (immune system process, developmental process, metabolic process, cell cycle and division, and regulation of transport) of these genes in cancer.

### Impact of novel eccDNA-driven drivers on tumorigenesis and prognosis

We next explored the impact of the novel eccDNA-driven drivers on tumorigenesis and prognosis. Of the 55 cancer-wide drivers (in more than six cancer types), 37 novel drivers according to CGC driver list were investigated. Differential expression analysis demonstrated their aberrant expression in cancer (S21A Fig). Moreover, novel cancer-wide drivers presented a rising or descending tendency of cancer stages in different cancer types (S21B Fig). Results of the pathway activity indicated that the novel cancer-wide drivers were significantly involved in famous cancer-related pathways (S21C Fig). Furthermore, we found the novel cancer-wide drivers were positively correlated with cell cycle, DNA damage, DNA repair, invasion while negatively related to states like angiogenesis, differentiation and stemness in most cancer types (S21D Fig). Next, we found high expression of most novel cancer-wide drivers was correlated to poor OS, PFS, DSS and DFI in different cancers (S21E Fig). These results collectively indicate the key role of the novel cancer-wide eccDNA-driven drivers in cell cycle, DNA damage and repair, and invasion by involvement in cancer-related pathways, impacting tumorigenesis, tumor development and patient prognosis.

### Clinical druggability of eccDNA-driven drivers

To explore the potential druggability and clinical relevance, we annotated the candidate drivers with respect to known drug-gene interactions using Drug-genes Interaction Database (DGIdb) [93]. Results showed that of 315 of the 432 drivers are druggable (275 with approved drugs and 40 with not approved drugs) (S22A Fig). Most of the gene-drug interaction types are inhibitor and agonist (directionality: inhibitory: 76.57%; activating: 23.43%) (S22B and C Fig). Although the proportion varied across cancer types, more than 60% of the candidate drivers are druggable (more than 50% with approved drugs) in all of the 12 cancer types (S22D Fig).

### eccDNA generation is associated with DNA methylation

**Fig 6:**
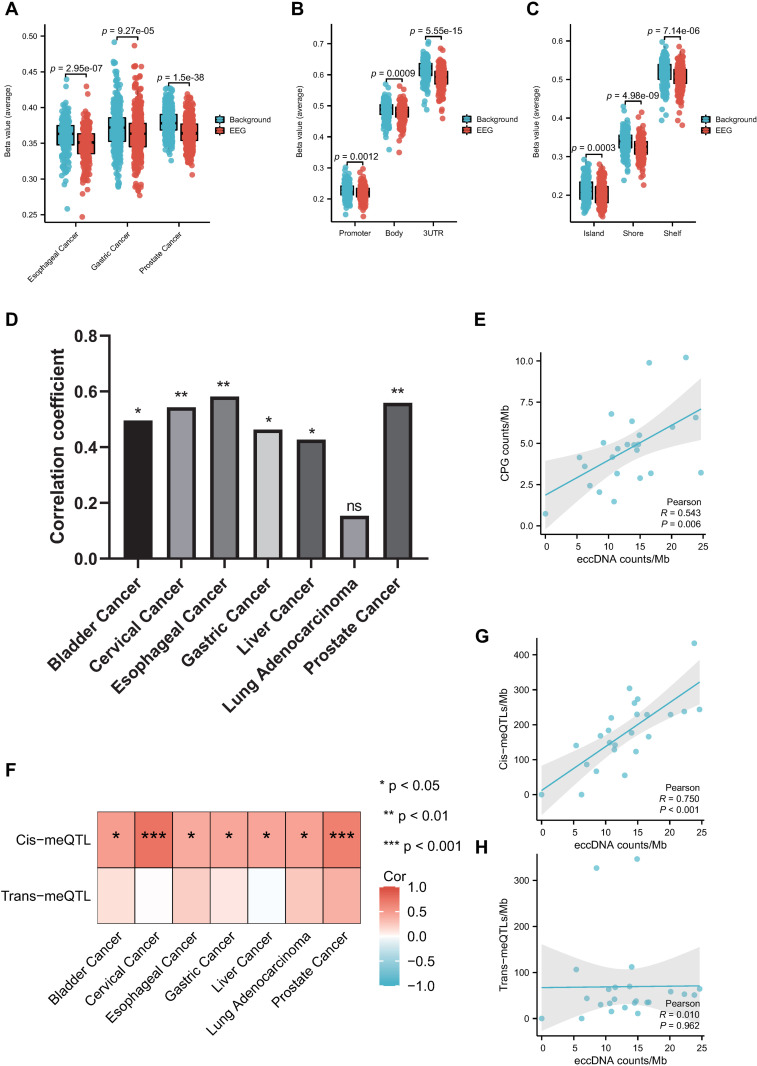
eccDNA generation is associated with methylation. (A) Comparison between DNA methylation levels of EEGs and the background in different cancers. (B, C) DNA methylation levels of EEGs and the background in terms of functional genomic distribution (B) and relation to CpG islands (C). Statistics were calculated with the Mann-Whitney U test (A, B, C). (D, E) Significant positive correlation between eccDNA/Mb and CpG island/Mb in different cancer types (D) like cervical cancer (E). (F-H) eccDNA/Mb is positively correlated with cis − meQTL/Mb, while not associated with trans−meQTL/Mb in different cancer types (F) like cervical cancer (G, H).

DNA methylation plays a crucial role in tumorigenesis by directing gene expression [51]. eccDNA was found enriched in CpG island [39]. We next explored the methylation level of EEGs in esophageal cancer, gastric cancer and prostate cancer. Results demonstrated that EEGs are hypo-methylated compared to the background level (Fig 6A). We further explored the methylation level of EEGs in terms of functional genomic distribution and relation to CpG islands in esophageal cancer. The promoter has the lowest methylation level while the 3’ UTR has the highest methylation level in functional genomic distribution groups (Fig 6B), and the island has the lowest methylation level while the shelf has the highest methylation level in groups of the relation to CpG islands (Fig 6C). In all groups, EEGs has a lower methylation level compared to the background (Fig 6B and C). We next examined the relation of eccDNAs and CpG islands. Correlation analysis further revealed a positive correlation between eccDNAs per Mb and CpG islands per Mb (Fig 6D and E). To gain insight into the bias of methylation on eccDNA production, we further investigate the relation of eccDNA and methylation quantitative trait loci (meQTL). The results showed that eccDNA/Mb was positively correlated with cis-meQTLs, while no correlation was found between eccDNA/Mb and trans-meQTLs, suggesting different mechanisms of cis-meQTLs and trans-meQTLs on eccDNA generation (Fig 6F–H).

### EDGs in non-cancerous diseases and health

eccDNA-driven oncogene overexpression has been proven prevalent in cancers. However, the identities and functions of EDGs in non-cancerous diseases and health of humans are still to be mined. Using our approach, we identified EDGs in diseases, including fetal growth restriction (FGR), pulmonary arterial hypertension (PAH), nuclear cataract with high myopia, and healthy samples including skeletal muscle, placenta, plasma, sperm, bone marrow mesenchymal stem cells (BMSCs) and thier differentiation into osteoblasts (OBs), adipocytes (ACs) and chondrocytes (CCs) [15–17,22,40]. DO enrichment analysis revealed that EDGs were enriched in related tissues (S23A and B Fig) or diseases (S23E and F Fig). GO enrichment analysis revealed that ERGs in different normal samples shared common terms like “regulation of transcription from RNA polymerase II promoter”, “regulation of transcription, DNA−templated”, “signal transduction”. KEGG enrichment analysis revealed shared terms like “Herpes simplex virus 1 infection”, “Rap1 signaling pathway” and “Motor proteins” (S23C and D Fig). The results of GO and KEGG analysis of EEGs in normal samples were shown in S24 Fig. Functional enrichment of EDGs in cancer and health revealed several common terms related to transcription, signal transduction and immune response, indicating some general functions of EDGs. Pathway and network enrichment analysis of ERGs in FGR using metascape showed these genes were enriched in pathways like “Herpes simplex virus 1 infection”, “copy number variation sydrome”, and “apoptosis” (S23G and H Fig) [94]. PPI and MCODE components showed the connections for these genes in FGR (S23I Fig). These findings demonstrated the applicability of the approach and general functions of EDGs in non-cancerous diseases and health.

## Discussion

EccDNA has been proven to drive oncogene overexpression via altering gene copy number or as functional regulatory elements changing chromosome interaction topology [1,2,7]. Based on the characteristic properties of eccDNAs, our study developed an approach for EDG discovery and reported a comprehensive analysis of them, which contribute to a more accurate map of the human genome. Our method was designed by integrating the latest understanding of eccDNA functions and regulatory mechanisms, such as its roles as enhancers or transcriptional templates [1,2]. Based on the genetic content, eccDNA can be categorized into: full gene eccDNA, exon eccDNA, intron eccDNA, repeat eccDNA, repeat-intergenic eccDNA, intergenic eccDNA, transposable element eccDNA, and promoter/enhancer eccDNA [4]. Full gene eccDNA is functional eccDNA and eccDNA contains other functional genomic elements may have content-related functions. Former study has also reported that 2% eccDNAs contained entire genes and 4% contained promoter regions and an initial exon in human germline, which could have phenotypic effects [70]. Furthermore, eccDNA could contain repetitive and unique elements to make a function. We also validated the reliability of our method by comparing with existing alternatives in HeLa by our full strategy and existing “partial” strategy. The comparison results demonstrate that our “full” method outperforms existing “partial” methods. The comparison of EEG and ERG expression demonstrated the dominant functional mode of eccDNA in tumors. We found ERGs are significantly more highly expressed than EEGs, suggesting that eccDNA may function more strongly as an enhancer regulating ERGs than as a transcriptional template for EEGs. This comparison helps reveal the functional preference of eccDNA in cancer, providing insights into the biological roles of eccDNAs and guidance for future research. Moreover, we supplemented a benchmark comparison of our methods with existing models. The benchmark results demonstrate that our method outperforms existing methods at precision and accuracy. Our method prioritizes high precision for the reliability of predictions over recall due to the domain-specific requirement of minimizing false positives. In clinical or translational applications like guiding targeted therapies, erroneous predictions (false positives) could lead to costly or harmful interventions. In the cancer driver prediction task, a precision-focused design ensures that predicted drivers are more likely to be biologically actionable, even if some true drivers are missed. Our methods exhibit a low recall and are not appropriate for recall-dependent cases. The root cause of low recall of our methods is strict thresholding. Our methods were designed to favor precision, and the higher threshold by “full” strategy and gene selection suppresses borderline predictions and reduces recall. Selecting more candidates would increase the recall while reduce the precision of the eccDriver method, and the tradeoff between precision and recall would be dependent on the specific scenarios. However, for scenarios in need of high recall, including all genes is better than a focused view of eccDNA considering that not all cancer driver genes are related to eccDNA. The analysis is limited for discarding potential active fractions of genes or enhancers. This may lead to the omission of certain active genes or enhancers, thereby influencing the comprehensiveness and accuracy of the results. Additionally, the dynamic activities of genes and enhancers may also have significant implications for the identification, functions and mechanisms of EDGs. Integrating more comprehensive functional genomics techniques (like ATAC-seq and ChIP-seq) and our growing understanding of genomic sequence functions will enhance the accuracy of the identification and characterization of EDGs. Nevertheless, considering our information about all necessary sequences is not complete, it represents the best effort so far for human EDG catalog and characterization.

For decades, substantial studies have explored cancer driver genes from different views [10,18,95]. Although the role of eccDNA in cancer development is attracting growing interest, a comprehensive profiling of driver genes from eccDNA view has not been done. Our study characterized EDGs in cancer. The expression of EDGs is significantly higher than the average by the mechanisms of high copy gene amplification and enhancer rewiring. Enrichment analysis revealed known and unknown general functions of EDGs in cancer, related to transcription regulation, signal transcription, immune response, cancer-related pathways, and so on, advancing our knowledge of eccDNA and EDG functions.

Hundreds of common regions were found in most cancers and 27 CEDGs were derived from these regions. Some CEDGs are well-known cancer driver genes. For example, CCND1 is well-known for its role in cell cycle G1/S transition and contributes to tumor development [83,96]. The CEDGs were abnormally expressed in most cancers. Besides, expression of most CEDGs is different in cancer subtypes and stages. Following results showed that the aberrant expression of CEDGs is attributed to their CNVs, as a result of eccDNA amplification, and methylation states. Most of the CEDGs present fairly high level of copy amplification, which affects their expression and patients’ prognosis across different cancer types. Hypomethylation of CEDGs also contribute to their aberrant expression. Based on the CEDGs, the altered group exhibited higher tumor ploidy, TMB and percentage of WGD compared to the unaltered group. The increased tumor ploidy and percentage of WGD percentage in the altered group suggest higher genomic instability. Genomic instability is a key driver of tumorigenesis and progression, often associated with chromosomal structural variations, copy number alterations, and WGD events [97]. These changes may lead to the amplification of oncogene, thereby promoting tumor aggressiveness and therapy resistance [73,97]. Additionally, WGD events play a crucial role in tumor evolution, potentially enhancing the adaptive evolution of tumor cells by increasing genomic plasticity [98]. The elevated TMB in the altered group indicates a higher mutation burden, which may enhance the responsiveness to immune checkpoint inhibitors [72]. Clinical studies have shown that patients with high-TMB tumors often exhibit better survival benefits following immunotherapy [99]. Therefore, tumors in the altered group may be more sensitive to immunotherapy, providing a rationale for personalized treatment strategies. Future studies are needed to further validate the clinical utility of these genomic features and explore their underlying mechanisms in tumor biology.

Another important result is that the CEDGs are significantly associated with cancer-related pathways like cell cycle and EMT. At single-cell level, CEDGs vigorously distinguish malignant cell type and positively related to functions like cell cycle, metastasis and invasion, revealing underlying mechanisms by which CEDGs contribute tumor formation and progression. Following analysis related to immune, microbiome and drug sensitivity further suggests the potential of CEDGs in cancer treatment. Mechanistically, CCND1 could regulate cell migration by modulating p27 levels and RhoA signaling [100]. By interaction with Gli2, CCND1 could promote cancer cell proliferation [101]. TAT-FADD could regulate anti-apoptotic and proinflammatory signaling through NF-Κb [102]. ANO1 inhibits ferroptosis by activating the PI3K-Akt signaling pathway, and recruits cancer-associated fibroblasts by regulating the TGF-β signaling pathway, which affects CD8 + T cell-mediated anti-tumor immunity and leads to resistance to immunotherapy [103]. FGF19 could cooperate with MYC to promote hepatocellular carcinoma development [104]. Construction of a prediction system for the pan-cancer prognosis is a challenging problem due to heterogeneity across cancer types [19,82]. Based on the constructed 17-CEDG signature, we found the CEDG scores were higher in tumors from gastro-intestinal tract and lower in tumors related to the urinary and reproductive system, indicating different expression patterns. Our model has fewer parameters or better performance compared with existing pan-cancer models [19,82]. Using the genes related to neutrophil extracellular traps (NETs), a 19-NET signature with near 0.7 AUC and related nomogram with 0.72 AUC were constructed, compared with our signature with 0.71 AUC and nomogram with 0.8 AUC at 3 years. A model exhibited similar performance compared with our model involving an endothelial cell senescence related signature with AUC of 0.72 and nomogram with near 0.78 AUC. However, it involves 50 genes, compared with 17 genes in our model. The good performance of this model suggests the CEDG score well quantifies tumor essence.

The eccDriver algorithm identifies 432 candidate eccDNA-driven drivers. The eccDNA-related characteristic properties will aid in future research and clinical application of these drivers. We found the candidate drivers primarily regulate five biological processes including immune system process, developmental process, metabolic process, cell cycle and division, and regulation of transport. eccDNA has been previously reported related to metabolic disorders, but not mechanistically explained [105]. eccDNA has been reported to trigger immune response dependent on its circularity [106]. Our study shows sequence context including enhancers and genes on eccDNA also regulate immune processes. MYC could interact with lncRNA EPIC1 to promote cell cycle progression in cancer [107]. EGFR might promote cancer development by activating IRE1a-XBP1s signaling and related EGFR targeting enhances chemotherapy efficiency [108]. KARS activates TBK1 through RALB to promote cancer cell survival [109]. Most of the candidate drivers identified are druggable. If clinically translated, 64% of the drivers are actionable with approved drugs for effective cancer therapies. A similar semi-local centrality model for cancer gene ranking was constructed by assessing the impact of gene mutations on the changes in gene expression patterns. Compared with their model including all potential cancer genes at a wider range, our methods focused on cancer genes related to eccDNA, providing insights from the view of eccDNA [110].

The EDGs also play important roles in non-cancerous diseases and health. Dysregulation of MED1 might impair the BMP/TGF-β signaling to influence the disease progression of PAH [111]. PPARG regulates the fate determination of stem cells [112]. STAT3 cooperates with MSX1 to promote the transcription of DLX5 to regulate skeletal development [113]. Future researches to explore the molecular mechanisms through which EDGs and eccDNA-driven drivers influence cancer metastasis, prognosis and treatment are needed. Additionally, more comprehensive clinical sample data will help to further elucidate the roles of eccDNA driver genes in various diseases and health conditions.

eccDNA has been reported to contain highly-accessible chromosome and be enriched in promoters and CpG islands [1,39]. Open chromosome structure is in accordance with low DNA methylation [114]. The reduced methylation levels of EEGs may be attribute to open chromosome structure of eccDNAs, particularly those enriched in functional genomic elements including promoters and CpG islands. Additionally, eccDNA generation is regulated by DNA repair, which has been reported as a mechanism for active DNA demethylation, reducing the methylation levels of EEGs on eccDNA [6,115].

We found eccDNA/Mb was positively correlated with CpG islands/Mb and cis-meQTLs/Mb. CpG islands are usually hypomethylated [116]. Cis-meQTLs usually locate near methylation sites like CpG islands and regulate their DNA methylation [117]. Related DNA methylation may regulate eccDNA formation by changing chromatin structure and genome stability, which influence the chance of DNA double-strand breaks (DSBs) and chromothripsis to promote eccDNA generation [118–124]. Decrease DNA Methylation 1 (DDM1) is an important regulatory factor for maintaining DNA methylation [125]. DDM1 has been reported to regulate the DSBs and accumulation of eccDNA through DNA methylation [123]. Transposable elements (TE) contribute to eccDNA biogenesis [126,127]. DNA methylation may also influence the TE activity to regulate eccDNA generation [123,128,129]. eccDNA function as mobile enhancer in cancer, by which DNA methylation could change chromosomal structure to regulate cancer development. DNA methylation could also regulate cancer development by changing oncogene copy numbers mediated by eccDNAs. Hypomethylation on eccDNA may enhance its transcriptional activity, thereby promoting oncogene expression and function [124]. Additionally, DNA hypomethylation may have an impact on genome stability by triggering high eccDNA level, which promotes cancer development [123].

Our study reported here provides an effective approach and comprehensive profiling of EDGs in human cancer, non-cancerous diseases and health, aids in advancing future researches and clinical application. We recognize that some EDGs, as important as the CEDGs, and some candidate eccDNA-driven drivers in specific cancer types are overlooked from a pan-cancer perspective. Future computational analysis and experiments are needed for further investigation and validation of EDGs, which is expected to have a positive impact on human life.

## Supporting information

S1 Fig Functional enrichment analysis and common eccDNA-driven gene (CEDG) identification in cancer. (A) Comparison of the lengths of eccDNAs meeting the inclusion criteria of the “full” strategy and the background of all eccDNAs involved in this study. (B) Comparison of the expression of ERGs identified by “full” and “partial” strategy by additional HeLa cell datasets. The “full” strategy used in our approach requires the entire enhancer to be located on the eccDNA, while the “partial” strategy of existing methods only requires partial overlap between the enhancer and the eccDNA. (C, D) GO (C) and KEGG pathway (D) analysis of ERGs to explore general functions in cancers. (E) Flower plot to identify CEDGs across different cancer types. (F) Differential expression of the 27 CEDGs in tumor and normal samples based on TCGA tumors. (G) Gene set variation analysis (GSVA) score in different cancers and related normal samples based on CEDG expression.
(PDF)

S2 Fig Functional enrichment analysis of EEGs in cancer. (A, B) GO (Biological process) (A) and KEGG pathway (B) analysis of EEGs to explore common pathways in cancers.
(PDF)

S3 Fig Characterization of differential EDGs in LUAD. (A) PPI network by STRING. (B, C) GO (B) and KEGG pathway (C) enrichment analysis reveal pathways related to cancer. (D) Top 10 hub genes of differential EDGs in LUAD by cytoscape. (E-F) Survival analysis of the top 10 hub genes in LUAD. High expression of all the 10 genes are significantly correlated to poor OS (E). Example KM plots of TRIP3 (F) and SPC25 (G) were demontrated.
(TIF)

S4 Fig Subtype, stage and survival characterization of CEDGs. (A) Heatmap showing subtype difference between high and low CEDG expression. (B, C) MRGPRF expression in subtype of HNSC (negative and positive)(B), and LUSC (basal, classical, primitive and secretory) (C). (D) GSVA scores in subtypes of BLCA, BRCA, COAD, GBM, HNSC, KIRC, LUAD, LUSC and STAD. (E) Expression differences of CEDGs between pathologic stages in TCGA tumors. (F) Trend plot showing the expression tendency of CEDGs in pathologic stages of TCGA tumors. (G) Box plot showing the escalating trend of MRGPRF expression in pathologic stages of BLCA (from stage I to stage IV). (H) Box plot showing the downward trend of KMT5B expression in pathologic stages of THCA (from stage I to stage IV). (I) GSVA score in stages of TCGA tumors. (J) Tendency of GSVA score among stages in TCGA tumors. (K) Survival (DFI, DSS, OS and PFI) analysis of CEDGs in TCGA tumors. (L-O) KM plots showing high ASS expression is related to poor DFI (L), DSS (M), OS (N) and PFI (O) of patient prognosis.
(TIF)

S5 Fig Differential expression and survival analysis of non-coding CEDGs. (A) Differential expression of the 18 non-coding CEDGs in tumor and normal samples based on TCGA tumors. (B) Survival analysis of non-coding CEDGs in TCGA tumors. (C) KM plots showing high RP11 − 802E16.3 expression is related to poor OS of KIRC patient.
(PDF)

S6 Fig The distribution of CEDG alterations in tumor samples by cbioportal. (TIF)

S7 Fig CNV percentage and survival analysis of CEDGs in TCGA cancers. (A) Heterozygous CNV in each cancer. (B) Homozygous CNV in each cancer. (C) DFI, DSS, OS and PFS analysis in each cancer.
(TIF)

S8 Fig CEDG expression in different alteration groups. (PDF)

S9 Fig Single nucleotide variation (SNV) analysis of CEDGs. (A) Mutation frequency of CEDGs. (B) Titv plot showing the distribution of transitions (Ti) and transversions (Tv) of CEDGs in each sample. (C) DFI, DSS, OS and PFS analysis in each cancer.
(PDF)

S10 Fig Non-deleterious mutation frequency of CEDGs. (PDF)

S11 Fig Methylation of CEDGs. (A) Methylation difference of CEDGs between tumor and normal samples in each cancer. (B) Correlation between methylation and mRNA expression. (C) Survival difference between high and low methylation in each cancer.
(PDF)

S12 Fig Activity assessment of cell cycle and EMT pathways in two examples of CEDGs. (A-C) Activity of cell cycle pathway between high and low expression group of MRPL21 in BRCA (A), LUAD (B) and STAD (C). (D-F) Activity of EMT pathway between high and low expression group of MRGPRF in BRCA (D), LUAD (E) and STAD (F).
(TIF)

S13 Fig Immune correlation of CEDGs. (A) The association between GSVA score and activity of cancer related pathways. (B, C) Correlation between CEDG expression and immune infiltrates in HNSC (B) and THYM (C). (D, E) Correlation between CEDG methylation and immune infiltrates in HNSC (D) and THYM (E). (F, G) Correlation between CEDG CNV and immune infiltrates in HNSC (F) and THYM (G). (H) The association between CEDGs and TMB in TCGA tumors. (I) Radar plot showing correlation of MYEOV and TMB in TCGA tumos.
(PDF)

S14 Fig Immune infiltration of gene set CNV and SNV for CEDGs. (A) The difference of immune infiltration between gene set CNV groups. (B) The difference of immune infiltration between gene set SNV groups.
(PDF)

S15 Fig Microbiota analysis of CEDGs in gastrointestinal cancers. (A) Correlation of CEDG expression and microbiota abundance. (B) Cluster map of CEDG-microbiota correlation.
(PDF)

S16 Fig Characterization of the 17-CEDG signature. (A) Forest plot showing the hazard ratio (HR) of every single gene for the prognosis in TCGA-training cohort. (B) LASSO regression and pairwise correlation analysis in the TCGA pan-cancer training set. (C) The distribution of the CEDG scores. (D) KM plot showing high CEDG score was significantly correlated to poor OS of patients in TCGA-testing cohort. (E) Prognostic performance of the 17-gene CEDG signature in TCGA-testing cohort. (F) Prognostic performance of the nomogram. (G, H) Correlation of CEDG score with cell cycle. (G) and EMT (H) score in different cancer types.
(PDF)

S17 Fig Drug sensitivity analysis of CEDGs in CTRP (A) and GDSC (B). (PDF)

S18 Fig Survival analysis of the highest-scoring eccDNA-driven drivers in HNSC (A), LIHC (B), GBM (C), OV (D), PAAD (E) and PRAD (F). (PDF)

S19 Fig The expression of the four genes (EGFR, KRAS, CDKN2A and H3C13) which scored the highest among the eccDNA-driven drivers in HNSC. (A-D) The expression of EGFR (A), KRAS (B), CDKN2A (C) and H3C13 (D) in TCGA HNSC tumor and normal samples. (E-H) Validation of the expression of EGFR (E), KRAS (F), CDKN2A (G) and H3C13 (H) in normal and different HNSC cell lines.
(PDF)

S20 Fig Biological processes of eccDNA-driven drivers. (A) Metabolic process network. (B) Cell cycle and division network.
(PDF)

S21 Fig Impact of novel eccDNA-driven drivers on tumorigenesis and prognosis. (A) Differential expression of the novel cancer-wide drivers in tumor and normal samples based on TCGA tumors. (B) Trend plot showing their expression tendency in pathologic stages of TCGA tumors. (C) The percentage of the effect of the novel cancer-wide drivers on cancer-related pathways. (D) Average correlations between the novel cancer-wide drivers and functional states in different cancers from CancerSEA. (E) Survival (DFI, DSS, OS and PFI) analysis of the novel cancer-wide drivers in TCGA tumors.
(PDF)

S22 Fig Clinical actionability of the candidate cancer drivers. (A) Clinical actionability of the 432 candidate drivers. (B) Directionality distribution of the gene-drug interactions. (C) Interaction types of the gene-drug interactions. (D) Clinical actionability of the candidate drivers by cancer type.
(PDF)

S23 Fig Enrichment and network analysis of EDGs in health and non-cancerous diseases. (A, B) Enrichment analysis of ERGs (A) and EEGs (B) in healthy samples to related tissue. (C, D) GO (C) and KEGG pathway (D)analysis of ERGs to explore common pathways in health. (E, F) Disease enrichment analysis of ERGs (E) and EEGs (F) in non-cancerous diseases to related diseases. (G, H) Enriched ontology clusters across studies (G), by p value (H left) and cluster ID (H right) by ERGs in FGR. (I) Protein-protein interaction (PPI) network (left) and PPI MCODE components (right) by ERGs in FGR.
(PDF)

S24 Fig Functional enrichment analysis EEGs in normal samples. (A, B) Enriched term of GO (Biological process) (A) and KEGG pathway (B) of EEGs in normal samples.
(PDF)

S1 Table List of RT-qPCR primer sequence used in this study. (XLSX)

S2 Table List of eccDNAs involved in this study. (XLSX)

S3 Table Upregulated EDGs in LUAD. (XLSX)

S4 Table List of the common regions and CEDGs in cancer. (XLSX)

S5 Table Non-deleterious mutations of the CEDGs in cancer. (XLSX)

S6 Table The top 50 candidate drivers in different cancer types. (XLSX)

## References

[1] Wu S, Turner KM, Nguyen N, Raviram R, Erb M, Santini J, et al. Circular ecDNA promotes accessible chromatin and high oncogene expression. Nature. 2019;575(7784):699–703. doi: 10.1038/s41586-019-1763-5 31748743 PMC7094777

[2] Morton AR, Dogan-Artun N, Faber ZJ, MacLeod G, Bartels CF, Piazza MS, et al. Functional enhancers shape extrachromosomal oncogene amplifications. Cell. 2019;179(6):1330–1341.e13. doi: 10.1016/j.cell.2019.10.039 31761532 PMC7241652

[3] Maurer BJ, Lai E, Hamkalo BA, Hood L, Attardi G. Novel submicroscopic extrachromosomal elements containing amplified genes in human cells. Nature. 1987;327(6121):434–7. doi: 10.1038/327434a0 3587364

[4] Ling X, Han Y, Meng J, Zhong B, Chen J, Zhang H, et al. Small extrachromosomal circular DNA (eccDNA): major functions in evolution and cancer. Mol Cancer. 2021;20(1):113. doi: 10.1186/s12943-021-01413-8 34479546 PMC8414719

[5] Verhaak RGW, Bafna V, Mischel PS. Extrachromosomal oncogene amplification in tumour pathogenesis and evolution. Nat Rev Cancer. 2019;19(5):283–8. doi: 10.1038/s41568-019-0128-6 30872802 PMC7168519

[6] Zhao Y, Yu L, Zhang S, Su X, Zhou X. Extrachromosomal circular DNA: Current status and future prospects. Elife. 2022;11:e81412. doi: 10.7554/eLife.81412 36256570 PMC9578701

[7] Kim H, Nguyen N-P, Turner K, Wu S, Gujar AD, Luebeck J, et al. Extrachromosomal DNA is associated with oncogene amplification and poor outcome across multiple cancers. Nat Genet. 2020;52(9):891–7. doi: 10.1038/s41588-020-0678-2 32807987 PMC7484012

[8] Chamorro González R, Conrad T, Stöber MC, Xu R, Giurgiu M, Rodriguez-Fos E, et al. Parallel sequencing of extrachromosomal circular DNAs and transcriptomes in single cancer cells. Nat Genet. 2023;55(5):880–90. doi: 10.1038/s41588-023-01386-y 37142849 PMC10181933

[9] Bal E, Kumar R, Hadigol M, Holmes AB, Hilton LK, Loh JW, et al. Super-enhancer hypermutation alters oncogene expression in B cell lymphoma. Nature. 2022;607(7920):808–15. doi: 10.1038/s41586-022-04906-8 35794478 PMC9583699

[10] Chen H, Li C, Peng X, Zhou Z, Weinstein JN, Cancer Genome Atlas Research Network, et al. A Pan-Cancer Analysis of Enhancer Expression in Nearly 9000 Patient Samples. Cell. 2018;173(2):386–399.e12. doi: 10.1016/j.cell.2018.03.027 29625054 PMC5890960

[11] Zhou J, Wang S, Nie D, Lai P, Li Y, Li Y, et al. Super-enhancer landscape reveals leukemia stem cell reliance on X-box binding protein 1 as a therapeutic vulnerability. Sci Transl Med. 2021;13(612):eabh3462. doi: 10.1126/scitranslmed.abh3462 34550724

[12] Zimmerman MW, Liu Y, He S, Durbin AD, Abraham BJ, Easton J, et al. MYC Drives a Subset of High-Risk Pediatric Neuroblastomas and Is Activated through Mechanisms Including Enhancer Hijacking and Focal Enhancer Amplification. Cancer Discov. 2018;8(3):320–35. doi: 10.1158/2159-8290.CD-17-0993 29284669 PMC5856009

[13] Xu Z, Lee D-S, Chandran S, Le VT, Bump R, Yasis J, et al. Structural variants drive context-dependent oncogene activation in cancer. Nature. 2022;612(7940):564–72. doi: 10.1038/s41586-022-05504-4 36477537 PMC9810360

[14] Hung KL, Yost KE, Xie L, Shi Q, Helmsauer K, Luebeck J, et al. ecDNA hubs drive cooperative intermolecular oncogene expression. Nature. 2021;600(7890):731–6. doi: 10.1038/s41586-021-04116-8 34819668 PMC9126690

[15] Møller HD, Mohiyuddin M, Prada-Luengo I, Sailani MR, Halling JF, Plomgaard P, et al. Circular DNA elements of chromosomal origin are common in healthy human somatic tissue. Nat Commun. 2018;9(1):1069. doi: 10.1038/s41467-018-03369-8 29540679 PMC5852086

[16] Yang H, He J, Huang S, Yang H, Yi Q, Tao Y, et al. Identification and Characterization of Extrachromosomal Circular DNA in Human Placentas With Fetal Growth Restriction. Front Immunol. 2021;12:780779. doi: 10.3389/fimmu.2021.780779 34992600 PMC8724250

[17] Zhang C, Du Q, Zhou X, Qu T, Liu Y, Ma K, et al. Differential expression and analysis of extrachromosomal circular DNAs as serum biomarkers in pulmonary arterial hypertension. Respir Res. 2024;25(1):181. doi: 10.1186/s12931-024-02808-z 38664836 PMC11046951

[18] Bailey MH, Tokheim C, Porta-Pardo E, Sengupta S, Bertrand D, Weerasinghe A, et al. Comprehensive characterization of cancer driver genes and mutations. Cell. 2018;173(2):371–385.e18. doi: 10.1016/j.cell.2018.02.060 29625053 PMC6029450

[19] Zhang Y, Guo L, Dai Q, Shang B, Xiao T, Di X, et al. A signature for pan-cancer prognosis based on neutrophil extracellular traps. J Immunother Cancer. 2022;10(6):e004210. doi: 10.1136/jitc-2021-004210 35688556 PMC9189842

[20] Corces MR, Granja JM, Shams S, Louie BH, Seoane JA, Zhou W, et al. The chromatin accessibility landscape of primary human cancers. Science. 2018;362(6413):eaav1898. doi: 10.1126/science.aav1898 30361341 PMC6408149

[21] Fu S, Dai Y, Zhang P, Zheng K, Cao G, Xu L, et al. Extrachromosomal circular DNA (eccDNA) characteristics in the bile and plasma of advanced perihilar cholangiocarcinoma patients and the construction of an eccDNA-related gene prognosis model. Front Cell Dev Biol. 2024;12:1379435. doi: 10.3389/fcell.2024.1379435 38903532 PMC11187006

[22] Zhong T, Wang W, Liu H, Zeng M, Zhao X, Guo Z. eccDNA Atlas: a comprehensive resource of eccDNA catalog. Brief Bioinform. 2023;24(2):bbad037. doi: 10.1093/bib/bbad037 36757087

[23] Li F, Yuan Q, Di W, Xia X, Liu Z, Mao N, et al. ERG orchestrates chromatin interactions to drive prostate cell fate reprogramming. J Clin Invest. 2020;130(11):5924–41. doi: 10.1172/JCI137967 32701507 PMC7598085

[24] Lim F, Solvason JJ, Ryan GE, Le SH, Jindal GA, Steffen P, et al. Affinity-optimizing enhancer variants disrupt development. Nature. 2024;626(7997):151–9. doi: 10.1038/s41586-023-06922-8 38233525 PMC10830414

[25] Dudnyk K, Cai D, Shi C, Xu J, Zhou J. Sequence basis of transcription initiation in the human genome. Science. 2024;384(6694):eadj0116. doi: 10.1126/science.adj0116 38662817 PMC11223672

[26] Fang M, Fang J, Luo S, Liu K, Yu Q, Yang J, et al. eccDNA-pipe: an integrated pipeline for identification, analysis and visualization of extrachromosomal circular DNA from high-throughput sequencing data. Brief Bioinform. 2024;25(2):bbae034. doi: 10.1093/bib/bbae034 38349061 PMC10862650

[27] Zhao S, Liu J, Nanga P, Liu Y, Cicek AE, Knoblauch N, et al. Detailed modeling of positive selection improves detection of cancer driver genes. Nat Commun. 2019;10(1):3399. doi: 10.1038/s41467-019-11284-9 31363082 PMC6667447

[28] Dietlein F, Weghorn D, Taylor-Weiner A, Richters A, Reardon B, Liu D, et al. Identification of cancer driver genes based on nucleotide context. Nat Genet. 2020;52(2):208–18. doi: 10.1038/s41588-019-0572-y 32015527 PMC7031046

[29] Bashashati A, Haffari G, Ding J, Ha G, Lui K, Rosner J, et al. DriverNet: uncovering the impact of somatic driver mutations on transcriptional networks in cancer. Genome Biol. 2012;13(12):R124. doi: 10.1186/gb-2012-13-12-r124 23383675 PMC4056374

[30] Shi X, Teng H, Shi L, Bi W, Wei W, Mao F, et al. Comprehensive evaluation of computational methods for predicting cancer driver genes. Brief Bioinform. 2022;23(2):bbab548. doi: 10.1093/bib/bbab548 35037014 PMC8921613

[31] Hou JP, Ma J. DawnRank: discovering personalized driver genes in cancer. Genome Med. 2014;6(7):56. doi: 10.1186/s13073-014-0056-8 25177370 PMC4148527

[32] Chen D, Lü L, Shang M-S, Zhang Y-C, Zhou T. Identifying influential nodes in complex networks. Physica A: Statistical Mechanics and its Applications. 2012;391(4):1777–87. doi: 10.1016/j.physa.2011.09.017

[33] Hou Y, Wang F, Cheng L, Luo T, Xu J, Wang H. Expression Profiles of SIRT1 and APP Genes in Human Neuroblastoma SK-N-SH Cells Treated with Two Epigenetic Agents. Neurosci Bull. 2016;32(5):455–62. doi: 10.1007/s12264-016-0052-7 27522594 PMC5563760

[34] Mattei AL, Bailly N, Meissner A. DNA methylation: a historical perspective. Trends Genet. 2022;38(7):676–707. doi: 10.1016/j.tig.2022.03.010 35504755

[35] Smith ZD, Hetzel S, Meissner A. DNA methylation in mammalian development and disease. Nat Rev Genet. 2025;26(1):7–30. doi: 10.1038/s41576-024-00760-8 39134824

[36] Gaudet F, Hodgson JG, Eden A, Jackson-Grusby L, Dausman J, Gray JW, et al. Induction of tumors in mice by genomic hypomethylation. Science. 2003;300(5618):489–92. doi: 10.1126/science.1083558 12702876

[37] Cai L, Bai H, Duan J, Wang Z, Gao S, Wang D, et al. Epigenetic alterations are associated with tumor mutation burden in non-small cell lung cancer. J Immunother Cancer. 2019;7(1):198. doi: 10.1186/s40425-019-0660-7 31349879 PMC6660715

[38] Xu GL, Bestor TH, Bourc’his D, Hsieh CL, Tommerup N, Bugge M, et al. Chromosome instability and immunodeficiency syndrome caused by mutations in a DNA methyltransferase gene. Nature. 1999;402(6758):187–91. doi: 10.1038/46052 10647011

[39] Dillon LW, Kumar P, Shibata Y, Wang Y-H, Willcox S, Griffith JD, et al. Production of Extrachromosomal MicroDNAs Is Linked to Mismatch Repair Pathways and Transcriptional Activity. Cell Rep. 2015;11(11):1749–59. doi: 10.1016/j.celrep.2015.05.020 26051933 PMC4481157

[40] Wen K, Zhang L, Cai Y, Teng H, Liang J, Yue Y, et al. Identification and characterization of extrachromosomal circular DNA in patients with high myopia and cataract. Epigenetics. 2023;18(1):2192324. doi: 10.1080/15592294.2023.2192324 36945837 PMC10038054

[41] Gao X, Liu K, Luo S, Tang M, Liu N, Jiang C, et al. Comparative analysis of methodologies for detecting extrachromosomal circular DNA. Nat Commun. 2024;15(1):9208. doi: 10.1038/s41467-024-53496-8 39448595 PMC11502736

[42] Fishilevich S, Nudel R, Rappaport N, Hadar R, Plaschkes I, Iny Stein T, et al. GeneHancer: genome-wide integration of enhancers and target genes in GeneCards. Database (Oxford). 2017;2017:bax028. doi: 10.1093/database/bax028 28605766 PMC5467550

[43] Karolchik D, Hinrichs AS, Furey TS, Roskin KM, Sugnet CW, Haussler D, et al. The UCSC Table Browser data retrieval tool. Nucleic Acids Res. 2004;32(Database issue):D493-6. doi: 10.1093/nar/gkh103 14681465 PMC308837

[44] Gill T, Wang H, Bandaru R, Lawlor M, Lu C, Nieman LT, et al. Selective targeting of MYC mRNA by stabilized antisense oligonucleotides. Oncogene. 2021;40(47):6527–39. doi: 10.1038/s41388-021-02053-4 34650218 PMC8627489

[45] Cancer Genome Atlas Research Network, Weinstein JN, Collisson EA, Mills GB, Shaw KRM, Ozenberger BA, et al. The Cancer Genome Atlas Pan-Cancer analysis project. Nat Genet. 2013;45(10):1113–20. doi: 10.1038/ng.2764 24071849 PMC3919969

[46] Miao Y-R, Zhang Q, Lei Q, Luo M, Xie G-Y, Wang H, et al. ImmuCellAI: A Unique Method for Comprehensive T-Cell Subsets Abundance Prediction and its Application in Cancer Immunotherapy. Adv Sci (Weinh). 2020;7(7):1902880. doi: 10.1002/advs.201902880 32274301 PMC7141005

[47] Li J, Lu Y, Akbani R, Ju Z, Roebuck PL, Liu W, et al. TCPA: a resource for cancer functional proteomics data. Nat Methods. 2013;10(11):1046–7. doi: 10.1038/nmeth.2650 24037243 PMC4076789

[48] Yang W, Soares J, Greninger P, Edelman EJ, Lightfoot H, Forbes S, et al. Genomics of Drug Sensitivity in Cancer (GDSC): a resource for therapeutic biomarker discovery in cancer cells. Nucleic Acids Res. 2013;41(Database issue):D955-61. doi: 10.1093/nar/gks1111 23180760 PMC3531057

[49] Basu A, Bodycombe NE, Cheah JH, Price EV, Liu K, Schaefer GI, et al. An interactive resource to identify cancer genetic and lineage dependencies targeted by small molecules. Cell. 2013;154(5):1151–61. doi: 10.1016/j.cell.2013.08.003 23993102 PMC3954635

[50] Dohlman AB, Arguijo Mendoza D, Ding S, Gao M, Dressman H, Iliev ID, et al. The cancer microbiome atlas: a pan-cancer comparative analysis to distinguish tissue-resident microbiota from contaminants. Cell Host Microbe. 2021;29(2):281-298.e5. doi: 10.1016/j.chom.2020.12.001 33382980 PMC7878430

[51] Gong J, Wan H, Mei S, Ruan H, Zhang Z, Liu C, et al. Pancan-meQTL: a database to systematically evaluate the effects of genetic variants on methylation in human cancer. Nucleic Acids Res. 2019;47(D1):D1066–72. doi: 10.1093/nar/gky814 30203047 PMC6323988

[52] Peng L, Zhou N, Zhang C-Y, Li G-C, Yuan X-Q. eccDNAdb: a database of extrachromosomal circular DNA profiles in human cancers. Oncogene. 2022;41(19):2696–705. doi: 10.1038/s41388-022-02286-x 35388171 PMC9076536

[53] Zhao X, Shi L, Ruan S, Bi W, Chen Y, Chen L, et al. CircleBase: an integrated resource and analysis platform for human eccDNAs. Nucleic Acids Res. 2022;50(D1):D72–82. doi: 10.1093/nar/gkab1104 34792166 PMC8728191

[54] Huang DW, Sherman BT, Tan Q, Kir J, Liu D, Bryant D, et al. DAVID Bioinformatics Resources: expanded annotation database and novel algorithms to better extract biology from large gene lists. Nucleic Acids Res. 2007;35(Web Server issue):W169–75. doi: 10.1093/nar/gkm415 17576678 PMC1933169

[55] Piñero J, Ramírez-Anguita JM, Saüch-Pitarch J, Ronzano F, Centeno E, Sanz F, et al. The DisGeNET knowledge platform for disease genomics: 2019 update. Nucleic Acids Res. 2020;48(D1):D845–55. doi: 10.1093/nar/gkz1021 31680165 PMC7145631

[56] Becker KG, Barnes KC, Bright TJ, Wang SA. The genetic association database. Nat Genet. 2004;36(5):431–2. doi: 10.1038/ng0504-431 15118671

[57] UniProt Consortium. UniProt: the Universal Protein Knowledgebase in 2023. Nucleic Acids Res. 2023;51(D1):D523–31. doi: 10.1093/nar/gkac1052 36408920 PMC9825514

[58] Su AI, Wiltshire T, Batalov S, Lapp H, Ching KA, Block D, et al. A gene atlas of the mouse and human protein-encoding transcriptomes. Proc Natl Acad Sci U S A. 2004;101(16):6062–7. doi: 10.1073/pnas.0400782101 15075390 PMC395923

[59] Uhlén M, Fagerberg L, Hallström BM, Lindskog C, Oksvold P, Mardinoglu A, et al. Proteomics. Tissue-based map of the human proteome. Science. 2015;347(6220):1260419. doi: 10.1126/science.1260419 25613900

[60] Bhuva DD, Tan CW, Liu N, Whitfield HJ, Papachristos N, Lee SC, et al. vissE: a versatile tool to identify and visualise higher-order molecular phenotypes from functional enrichment analysis. BMC Bioinformatics. 2024;25(1):64. doi: 10.1186/s12859-024-05676-y 38331751 PMC10854147

[61] Liu C-J, Hu F-F, Xie G-Y, Miao Y-R, Li X-W, Zeng Y, et al. GSCA: an integrated platform for gene set cancer analysis at genomic, pharmacogenomic and immunogenomic levels. Brief Bioinform. 2023;24(1):bbac558. doi: 10.1093/bib/bbac558 36549921

[62] Akbani R, Ng PKS, Werner HMJ, Shahmoradgoli M, Zhang F, Ju Z, et al. A pan-cancer proteomic perspective on The Cancer Genome Atlas. Nat Commun. 2014;5:3887. doi: 10.1038/ncomms4887 24871328 PMC4109726

[63] Ye Y, Xiang Y, Ozguc FM, Kim Y, Liu C-J, Park PK, et al. The Genomic Landscape and Pharmacogenomic Interactions of Clock Genes in Cancer Chronotherapy. Cell Syst. 2018;6(3):314-328.e2. doi: 10.1016/j.cels.2018.01.013 29525205 PMC6056007

[64] Sondka Z, Dhir N, Carvalho-Silva D, Jupe S, Madhumita, McLaren K, et al. COSMIC: a curated database of somatic variants and clinical data for cancer. Nucleic acids research. 2024;52:D1210-D1217. doi: 10.1093/nar/gkad986 PMC10767972 38183204

[65] Oh S, Shao J, Mitra J, Xiong F, D’Antonio M, Wang R, et al. Enhancer release and retargeting activates disease-susceptibility genes. Nature. 2021;595(7869):735–40. doi: 10.1038/s41586-021-03577-1 34040254 PMC11171441

[66] Koche RP, Rodriguez-Fos E, Helmsauer K, Burkert M, MacArthur IC, Maag J, et al. Extrachromosomal circular DNA drives oncogenic genome remodeling in neuroblastoma. Nat Genet. 2020;52(1):29–34. doi: 10.1038/s41588-019-0547-z 31844324 PMC7008131

[67] Szklarczyk D, Kirsch R, Koutrouli M, Nastou K, Mehryary F, Hachilif R, et al. The STRING database in 2023: protein-protein association networks and functional enrichment analyses for any sequenced genome of interest. Nucleic Acids Res. 2023;51(D1):D638–46. doi: 10.1093/nar/gkac1000 36370105 PMC9825434

[68] Shannon P, Markiel A, Ozier O, Baliga NS, Wang JT, Ramage D, et al. Cytoscape: a software environment for integrated models of biomolecular interaction networks. Genome Res. 2003;13(11):2498–504. doi: 10.1101/gr.1239303 14597658 PMC403769

[69] Cerami E, Gao J, Dogrusoz U, Gross BE, Sumer SO, Aksoy BA, et al. The cBio cancer genomics portal: an open platform for exploring multidimensional cancer genomics data. Cancer Discov. 2012;2(5):401–4. doi: 10.1158/2159-8290.CD-12-0095 22588877 PMC3956037

[70] Henriksen RA, Jenjaroenpun P, Sjøstrøm IB, Jensen KR, Prada-Luengo I, Wongsurawat T, et al. Circular DNA in the human germline and its association with recombination. Mol Cell. 2022;82(1):209–217.e7. doi: 10.1016/j.molcel.2021.11.027 34951964 PMC10707452

[71] Li Y, Li B, Xu B, Han B, Xia H, Chen Q-M, et al. Expression of p53, p21(CIP1/WAF1) and eIF4E in the adjacent tissues of oral squamous cell carcinoma: establishing the molecular boundary and a cancer progression model. Int J Oral Sci. 2015;7(3):161–8. doi: 10.1038/ijos.2015.5 25835715 PMC4582560

[72] Zhang C, Zhang Z, Sun N, Zhang Z, Zhang G, Wang F, et al. Identification of a costimulatory molecule-based signature for predicting prognosis risk and immunotherapy response in patients with lung adenocarcinoma. Oncoimmunology. 2020;9(1):1824641. doi: 10.1080/2162402X.2020.1824641 33457102 PMC7781839

[73] Berenjeno IM, Piñeiro R, Castillo SD, Pearce W, McGranahan N, Dewhurst SM, et al. Oncogenic PIK3CA induces centrosome amplification and tolerance to genome doubling. Nat Commun. 2017;8(1):1773. doi: 10.1038/s41467-017-02002-4 29170395 PMC5701070

[74] Yuan H, Yan M, Zhang G, Liu W, Deng C, Liao G, et al. CancerSEA: a cancer single-cell state atlas. Nucleic Acids Res. 2019;47(D1):D900–8. doi: 10.1093/nar/gky939 30329142 PMC6324047

[75] Tirosh I, Izar B, Prakadan SM, Wadsworth MH 2nd, Treacy D, Trombetta JJ, et al. Dissecting the multicellular ecosystem of metastatic melanoma by single-cell RNA-seq. Science. 2016;352(6282):189–96. doi: 10.1126/science.aad0501 27124452 PMC4944528

[76] Durante MA, Rodriguez DA, Kurtenbach S, Kuznetsov JN, Sanchez MI, Decatur CL, et al. Single-cell analysis reveals new evolutionary complexity in uveal melanoma. Nat Commun. 2020;11(1):496. doi: 10.1038/s41467-019-14256-1 31980621 PMC6981133

[77] Puram SV, Tirosh I, Parikh AS, Patel AP, Yizhak K, Gillespie S, et al. Single-Cell Transcriptomic Analysis of Primary and Metastatic Tumor Ecosystems in Head and Neck Cancer. Cell. 2017;171(7):1611–1624.e24. doi: 10.1016/j.cell.2017.10.044 29198524 PMC5878932

[78] Ascierto PA, Lewis KD, Di Giacomo AM, Demidov L, Mandalà M, Bondarenko I, et al. Prognostic impact of baseline tumour immune infiltrate on disease-free survival in patients with completely resected, BRAFv600 mutation-positive melanoma receiving adjuvant vemurafenib. Ann Oncol. 2020;31(1):153–9. doi: 10.1016/j.annonc.2019.10.002 31912791

[79] Liu X, Xu J, Zhang B, Liu J, Liang C, Meng Q, et al. The reciprocal regulation between host tissue and immune cells in pancreatic ductal adenocarcinoma: new insights and therapeutic implications. Mol Cancer. 2019;18(1):184. doi: 10.1186/s12943-019-1117-9 31831007 PMC6909567

[80] Wang S-S, Liu W, Ly D, Xu H, Qu L, Zhang L. Tumor-infiltrating B cells: their role and application in anti-tumor immunity in lung cancer. Cell Mol Immunol. 2019;16(1):6–18. doi: 10.1038/s41423-018-0027-x 29628498 PMC6318290

[81] Sepich-Poore GD, Zitvogel L, Straussman R, Hasty J, Wargo JA, Knight R. The microbiome and human cancer. Science. 2021;371(6536):eabc4552. doi: 10.1126/science.abc4552 33766858 PMC8767999

[82] Wu Z, Uhl B, Gires O, Reichel CA. A transcriptomic pan-cancer signature for survival prognostication and prediction of immunotherapy response based on endothelial senescence. J Biomed Sci. 2023;30(1):21. doi: 10.1186/s12929-023-00915-5 36978029 PMC10045484

[83] Sanchez-Vega F, Mina M, Armenia J, Chatila WK, Luna A, La KC, et al. Oncogenic Signaling Pathways in The Cancer Genome Atlas. Cell. 2018;173(2):321–337.e10. doi: 10.1016/j.cell.2018.03.035 29625050 PMC6070353

[84] Yu T-J, Ma D, Liu Y-Y, Xiao Y, Gong Y, Jiang Y-Z, et al. Bulk and single-cell transcriptome profiling reveal the metabolic heterogeneity in human breast cancers. Mol Ther. 2021;29(7):2350–65. doi: 10.1016/j.ymthe.2021.03.003 33677091 PMC8261089

[85] Pradella D, Zhang M, Gao R, Yao MA, Gluchowska KM, Cendon-Florez Y, et al. Engineered extrachromosomal oncogene amplifications promote tumorigenesis. Nature. 2025;637(8047):955–64. doi: 10.1038/s41586-024-08318-8 39695225 PMC11754114

[86] Pal Choudhuri S, Girard L, Lim JYS, Wise JF, Freitas B, Yang D, et al. Acquired Cross-Resistance in Small Cell Lung Cancer due to Extrachromosomal DNA Amplification of MYC Paralogs. Cancer Discov. 2024;14(5):804–27. doi: 10.1158/2159-8290.CD-23-0656 38386926 PMC11061613

[87] Mortenson KL, Dawes C, Wilson ER, Patchen NE, Johnson HE, Gertz J, et al. 3D genomic analysis reveals novel enhancer-hijacking caused by complex structural alterations that drive oncogene overexpression. Nat Commun. 2024;15(1):6130. doi: 10.1038/s41467-024-50387-w 39033128 PMC11271278

[88] Lu M, Liu B, Li D, Gao Z, Li W, Zhou X, et al. PXDNL activates the motility of urothelial bladder carcinoma cells through the Wnt/β-catenin pathway and has a prognostic value. Life Sci. 2023;312:121270. doi: 10.1016/j.lfs.2022.121270 36493879

[89] Pongor L, Kormos M, Hatzis C, Pusztai L, Szabó A, Győrffy B. A genome-wide approach to link genotype to clinical outcome by utilizing next generation sequencing and gene chip data of 6,697 breast cancer patients. Genome Med. 2015;7:104. doi: 10.1186/s13073-015-0228-1 26474971 PMC4609150

[90] Tian W, Luo Y, Tang Y, Kong Y, Wu L, Zheng S, et al. Novel Implication of the Basement Membrane for Breast Cancer Outcome and Immune Infiltration. Int J Biol Sci. 2023;19(5):1645–63. doi: 10.7150/ijbs.81939 37056938 PMC10086744

[91] Zhao H, Yin X, Wang L, Liu K, Liu W, Bo L, et al. Identifying tumour microenvironment-related signature that correlates with prognosis and immunotherapy response in breast cancer. Sci Data. 2023;10(1):119. doi: 10.1038/s41597-023-02032-2 36869083 PMC9984471

[92] Li Y, Jiao Y, Luo Z, Li Y, Liu Y. High peroxidasin-like expression is a potential and independent prognostic biomarker in breast cancer. Medicine (Baltimore). 2019;98(44):e17703. doi: 10.1097/MD.0000000000017703 31689799 PMC6946426

[93] Cannon M, Stevenson J, Stahl K, Basu R, Coffman A, Kiwala S, et al. DGIdb 5.0: rebuilding the drug-gene interaction database for precision medicine and drug discovery platforms. Nucleic Acids Res. 2024;52(D1):D1227–35. doi: 10.1093/nar/gkad1040 37953380 PMC10767982

[94] Zhou Y, Zhou B, Pache L, Chang M, Khodabakhshi AH, Tanaseichuk O, et al. Metascape provides a biologist-oriented resource for the analysis of systems-level datasets. Nat Commun. 2019;10(1):1523. doi: 10.1038/s41467-019-09234-6 30944313 PMC6447622

[95] Martínez-Jiménez F, Muiños F, Sentís I, Deu-Pons J, Reyes-Salazar I, Arnedo-Pac C, et al. A compendium of mutational cancer driver genes. Nat Rev Cancer. 2020;20(10):555–72. doi: 10.1038/s41568-020-0290-x 32778778

[96] Wiestner A, Tehrani M, Chiorazzi M, Wright G, Gibellini F, Nakayama K, et al. Point mutations and genomic deletions in CCND1 create stable truncated cyclin D1 mRNAs that are associated with increased proliferation rate and shorter survival. Blood. 2007;109(11):4599–606. doi: 10.1182/blood-2006-08-039859 17299095 PMC1885523

[97] Liu Y, Sethi NS, Hinoue T, Schneider BG, Cherniack AD, Sanchez-Vega F, et al. Comparative Molecular Analysis of Gastrointestinal Adenocarcinomas. Cancer Cell. 2018;33(4):721–35.e8. doi: 10.1016/j.ccell.2018.03.010 29622466 PMC5966039

[98] Espejo Valle-Inclan J, De Noon S, Trevers K, Elrick H, van Belzen IAEM, Zumalave S, et al. Ongoing chromothripsis underpins osteosarcoma genome complexity and clonal evolution. Cell. 2025;188(2):352–70.e22. doi: 10.1016/j.cell.2024.12.005 39814020

[99] Wood MA, Weeder BR, David JK, Nellore A, Thompson RF. Burden of tumor mutations, neoepitopes, and other variants are weak predictors of cancer immunotherapy response and overall survival. Genome Med. 2020;12(1):33. doi: 10.1186/s13073-020-00729-2 32228719 PMC7106909

[100] Fusté NP, Fernández-Hernández R, Cemeli T, Mirantes C, Pedraza N, Rafel M, et al. Cytoplasmic cyclin D1 regulates cell invasion and metastasis through the phosphorylation of paxillin. Nat Commun. 2016;7:11581. doi: 10.1038/ncomms11581 27181366 PMC4873647

[101] Guerrero-Preston R, Michailidi C, Marchionni L, Pickering CR, Frederick MJ, Myers JN, et al. Key tumor suppressor genes inactivated by “greater promoter” methylation and somatic mutations in head and neck cancer. Epigenetics. 2014;9(7):1031–46. doi: 10.4161/epi.29025 24786473 PMC4143405

[102] Ranjan K, Waghela BN, Vaidya FU, Pathak C. Cell-Penetrable Peptide-Conjugated FADD Induces Apoptosis and Regulates Inflammatory Signaling in Cancer Cells. Int J Mol Sci. 2020;21(18):6890. doi: 10.3390/ijms21186890 32961826 PMC7555701

[103] Jiang F, Jia K, Chen Y, Ji C, Chong X, Li Z, et al. ANO1-Mediated Inhibition of Cancer Ferroptosis Confers Immunotherapeutic Resistance through Recruiting Cancer-Associated Fibroblasts. Adv Sci (Weinh). 2023;10(24):e2300881. doi: 10.1002/advs.202300881 37341301 PMC10460848

[104] Ursic-Bedoya J, Desandré G, Chavey C, Marie P, Polizzi A, Rivière B, et al. FGF19 and its analog Aldafermin cooperate with MYC to induce aggressive hepatocarcinogenesis. EMBO Mol Med. 2024;16(2):238–50. doi: 10.1038/s44321-023-00021-x 38228803 PMC10897482

[105] Celec P, Janovičová Ĺ, Gurecká R, Koborová I, Gardlík R, Šebeková K. Circulating extracellular DNA is in association with continuous metabolic syndrome score in healthy adolescents. Physiol Genomics. 2021;53(7):309–18. doi: 10.1152/physiolgenomics.00029.2021 34097532

[106] Wang Y, Wang M, Djekidel MN, Chen H, Liu D, Alt FW, et al. eccDNAs are apoptotic products with high innate immunostimulatory activity. Nature. 2021;599(7884):308–14. doi: 10.1038/s41586-021-04009-w 34671165 PMC9295135

[107] Wang Z, Yang B, Zhang M, Guo W, Wu Z, Wang Y, et al. lncRNA Epigenetic Landscape Analysis Identifies EPIC1 as an Oncogenic lncRNA that Interacts with MYC and Promotes Cell-Cycle Progression in Cancer. Cancer Cell. 2018;33(4):706–20.e9. doi: 10.1016/j.ccell.2018.03.006 29622465 PMC6143179

[108] Huo M, Zhao Y, Liu X, Gao Y, Zhang D, Chang M, et al. EGFR targeting enhances the efficiency of chemotherapy through inhibiting IRE1α-XBP1s pathway in colorectal cancer cells. J Cancer. 2020;11(15):4464–73. doi: 10.7150/jca.44234 32489465 PMC7255363

[109] Rajurkar M, Dang K, Fernandez-Barrena MG, Liu X, Fernandez-Zapico ME, Lewis BC, et al. IKBKE Is Required during KRAS-Induced Pancreatic Tumorigenesis. Cancer Res. 2017;77(2):320–9. doi: 10.1158/0008-5472.CAN-15-1684 28069799 PMC5243176

[110] Kolbe M, Torres Alavez JA, Mottram R, Bintanja R, van der Linden EC, Stendel M. Model performance and surface impacts of atmospheric river events in Antarctica. Discov Atmos. 2025;3(1):4. doi: 10.1007/s44292-025-00026-w 40130261 PMC11931731

[111] Wang C, Xing Y, Zhang J, He M, Dong J, Chen S, et al. MED1 Regulates BMP/TGF-β in Endothelium: Implication for Pulmonary Hypertension. Circ Res. 2022;131(10):828–41. doi: 10.1161/CIRCRESAHA.122.321532 36252121

[112] Jin Y, Liu Z, Cao W, Ma X, Fan Y, Yu Y, et al. Novel functional MAR elements of double minute chromosomes in human ovarian cells capable of enhancing gene expression. PLoS One. 2012;7(2):e30419. doi: 10.1371/journal.pone.0030419 22319568 PMC3272018

[113] Zhou S, Dai Q, Huang X, Jin A, Yang Y, Gong X, et al. STAT3 is critical for skeletal development and bone homeostasis by regulating osteogenesis. Nat Commun. 2021;12(1):6891. doi: 10.1038/s41467-021-27273-w 34824272 PMC8616950

[114] Rönn T, Ofori JK, Perfilyev A, Hamilton A, Pircs K, Eichelmann F, et al. Genes with epigenetic alterations in human pancreatic islets impact mitochondrial function, insulin secretion, and type 2 diabetes. Nat Commun. 2023;14(1):8040. doi: 10.1038/s41467-023-43719-9 38086799 PMC10716521

[115] Guo JU, Su Y, Zhong C, Ming G, Song H. Hydroxylation of 5-methylcytosine by TET1 promotes active DNA demethylation in the adult brain. Cell. 2011;145(3):423–34. doi: 10.1016/j.cell.2011.03.022 21496894 PMC3088758

[116] Varley KE, Gertz J, Bowling KM, Parker SL, Reddy TE, Pauli-Behn F, et al. Dynamic DNA methylation across diverse human cell lines and tissues. Genome Res. 2013;23(3):555–67. doi: 10.1101/gr.147942.112 23325432 PMC3589544

[117] Villicaña S, Castillo-Fernandez J, Hannon E, Christiansen C, Tsai P-C, Maddock J, et al. Genetic impacts on DNA methylation help elucidate regulatory genomic processes. Genome Biol. 2023;24(1):176. doi: 10.1186/s13059-023-03011-x 37525248 PMC10391992

[118] Canela A, Maman Y, Huang S-YN, Wutz G, Tang W, Zagnoli-Vieira G, et al. Topoisomerase II-Induced Chromosome Breakage and Translocation Is Determined by Chromosome Architecture and Transcriptional Activity. Mol Cell. 2019;75(2):252–66.e8. doi: 10.1016/j.molcel.2019.04.030 31202577 PMC8170508

[119] Gothe HJ, Bouwman BAM, Gusmao EG, Piccinno R, Petrosino G, Sayols S, et al. Spatial Chromosome Folding and Active Transcription Drive DNA Fragility and Formation of Oncogenic MLL Translocations. Mol Cell. 2019;75(2):267–83.e12. doi: 10.1016/j.molcel.2019.05.015 31202576

[120] Arnould C, Rocher V, Finoux A-L, Clouaire T, Li K, Zhou F, et al. Loop extrusion as a mechanism for formation of DNA damage repair foci. Nature. 2021;590(7847):660–5. doi: 10.1038/s41586-021-03193-z 33597753 PMC7116834

[121] Canela A, Maman Y, Jung S, Wong N, Callen E, Day A, et al. Genome Organization Drives Chromosome Fragility. Cell. 2017;170(3):507–21.e18. doi: 10.1016/j.cell.2017.06.034 28735753 PMC6133249

[122] Wilkinson AL, Zorzan I, Rugg-Gunn PJ. Epigenetic regulation of early human embryo development. Cell Stem Cell. 2023;30(12):1569–84. doi: 10.1016/j.stem.2023.09.010 37858333

[123] Zhang P, Mbodj A, Soundiramourtty A, Llauro C, Ghesquière A, Ingouff M, et al. Extrachromosomal circular DNA and structural variants highlight genome instability in Arabidopsis epigenetic mutants. Nat Commun. 2023;14(1):5236. doi: 10.1038/s41467-023-41023-0 37640706 PMC10462705

[124] Schübeler D. Function and information content of DNA methylation. Nature. 2015;517(7534):321–6. doi: 10.1038/nature14192 25592537

[125] Liu Y, Zhang Z, Hu H, Chen W, Zhang F, Wang Q, et al. Molecular basis of chromatin remodelling by DDM1 involved in plant DNA methylation. Nat Plants. 2024;10(3):374–80. doi: 10.1038/s41477-024-01640-z 38413824

[126] Lanciano S, Carpentier M-C, Llauro C, Jobet E, Robakowska-Hyzorek D, Lasserre E, et al. Sequencing the extrachromosomal circular mobilome reveals retrotransposon activity in plants. PLoS Genet. 2017;13(2):e1006630. doi: 10.1371/journal.pgen.1006630 28212378 PMC5338827

[127] Møller HD, Larsen CE, Parsons L, Hansen AJ, Regenberg B, Mourier T. Formation of Extrachromosomal Circular DNA from Long Terminal Repeats of Retrotransposons in Saccharomyces cerevisiae. G3 (Bethesda). 2015;6(2):453–62. doi: 10.1534/g3.115.025858 26681518 PMC4751563

[128] Zhao L, Zhou Q, He L, Deng L, Lozano-Duran R, Li G, et al. DNA methylation underpins the epigenomic landscape regulating genome transcription in Arabidopsis. Genome Biol. 2022;23(1):197. doi: 10.1186/s13059-022-02768-x 36127735 PMC9487137

[129] Kolkman JM, Conrad LJ, Farmer PR, Hardeman K, Ahern KR, Lewis PE, et al. Distribution of Activator (Ac) throughout the maize genome for use in regional mutagenesis. Genetics. 2005;169(2):981–95. doi: 10.1534/genetics.104.033738 15520264 PMC1449104

