# Supplementary figures and images for: Identification and characterization of eccDNA-driven genes in humans
Source: PLoS One. 2025 Jun 6;20(6):e0324438. doi: 10.1371/journal.pone.0324438 (PMC12143510; doi:10.1371/journal.pone.0324438)

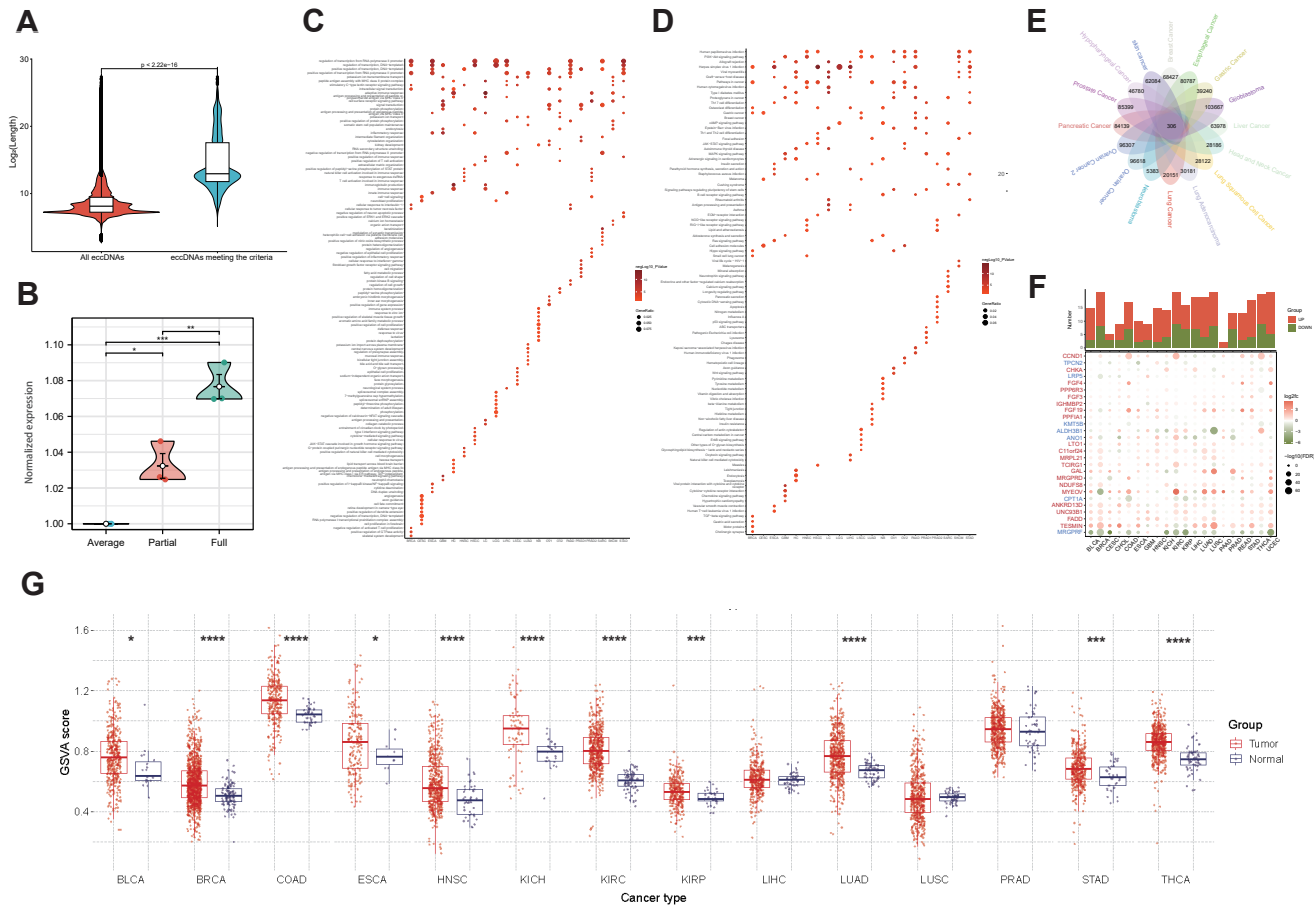

Supplement: S1 Fig — (A) Comparison of the lengths of eccDNAs meeting the inclusion criteria of the “full” strategy and the background of all eccDNAs involved in this study. (B) Comparison of the expression of ERGs identified by “full” and “partial” strategy by additional HeLa cell datasets. The “full” strategy used in our approach requires the entire enhancer to be located on the eccDNA, while the “partial” strategy of existing methods only requires partial overlap between the enhancer and the eccDNA. (C, D) GO (C) and KEGG pathway (D) analysis of ERGs to explore general functions in cancers. (E) Flower plot to identify CEDGs across different cancer types. (F) Differential expression of the 27 CEDGs in tumor and normal samples based on TCGA tumors. (G) Gene set variation analysis (GSVA) score in different cancers and related normal samples based on CEDG expression. (PDF) [file pone.0324438.s001.pdf]

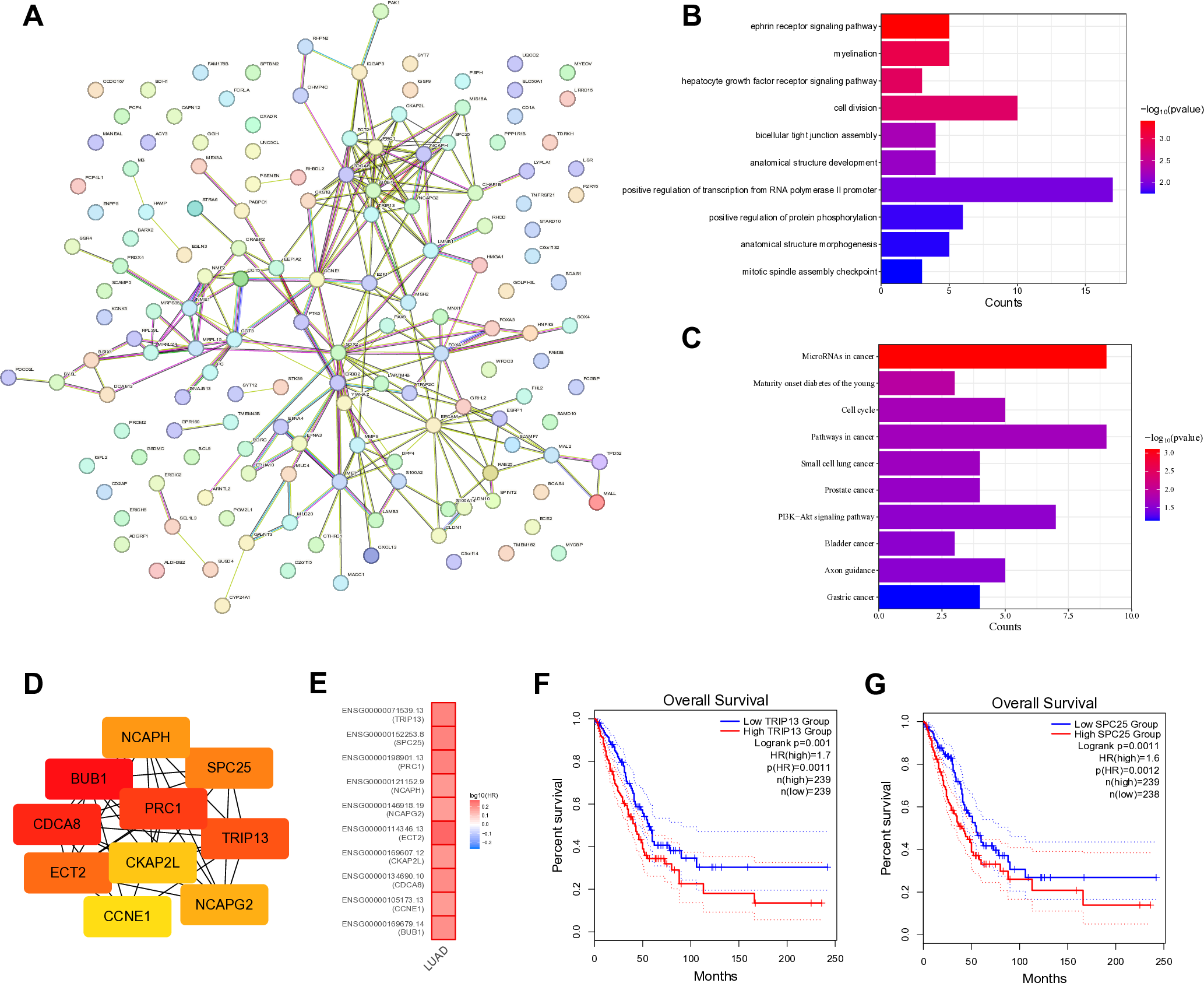

Supplement: S3 Fig — (A) PPI network by STRING. (B, C) GO (B) and KEGG pathway (C) enrichment analysis reveal pathways related to cancer. (D) Top 10 hub genes of differential EDGs in LUAD by cytoscape. (E-F) Survival analysis of the top 10 hub genes in LUAD. High expression of all the 10 genes are significantly correlated to poor OS (E). Example KM plots of TRIP3 (F) and SPC25 (G) were demontrated. (TIF) [file pone.0324438.s003.tif]

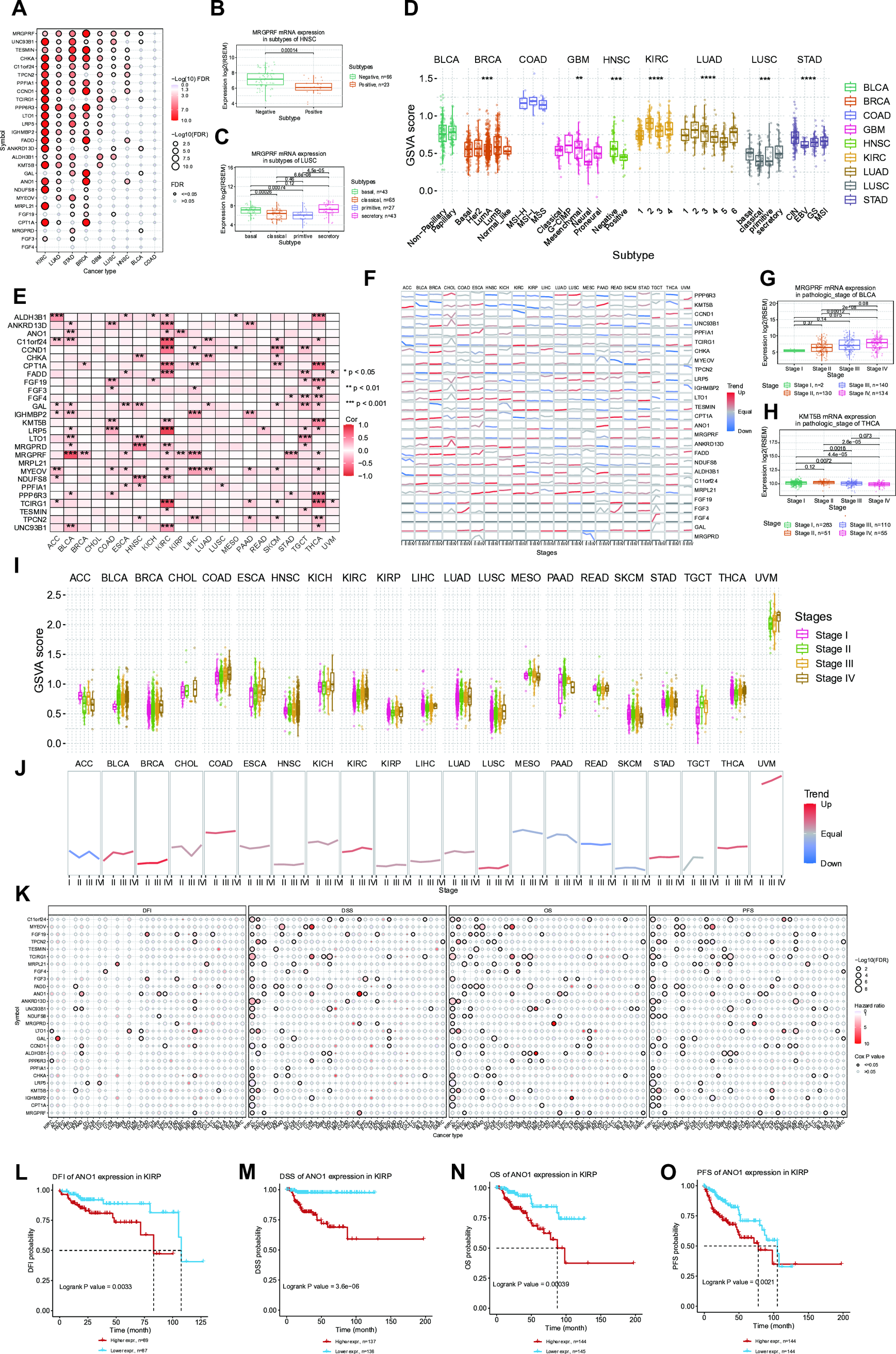

Supplement: S4 Fig — (A) Heatmap showing subtype difference between high and low CEDG expression. (B, C) MRGPRF expression in subtype of HNSC (negative and positive)(B), and LUSC (basal, classical, primitive and secretory) (C). (D) GSVA scores in subtypes of BLCA, BRCA, COAD, GBM, HNSC, KIRC, LUAD, LUSC and STAD. (E) Expression differences of CEDGs between pathologic stages in TCGA tumors. (F) Trend plot showing the expression tendency of CEDGs in pathologic stages of TCGA tumors. (G) Box plot showing the escalating trend of MRGPRF expression in pathologic stages of BLCA (from stage I to stage IV). (H) Box plot showing the downward trend of KMT5B expression in pathologic stages of THCA (from stage I to stage IV). (I) GSVA score in stages of TCGA tumors. (J) Tendency of GSVA score among stages in TCGA tumors. (K) Survival (DFI, DSS, OS and PFI) analysis of CEDGs in TCGA tumors. (L-O) KM plots showing high ASS expression is related to poor DFI (L), DSS (M), OS (N) and PFI (O) of patient prognosis. (TIF) [file pone.0324438.s004.tif]

**A**

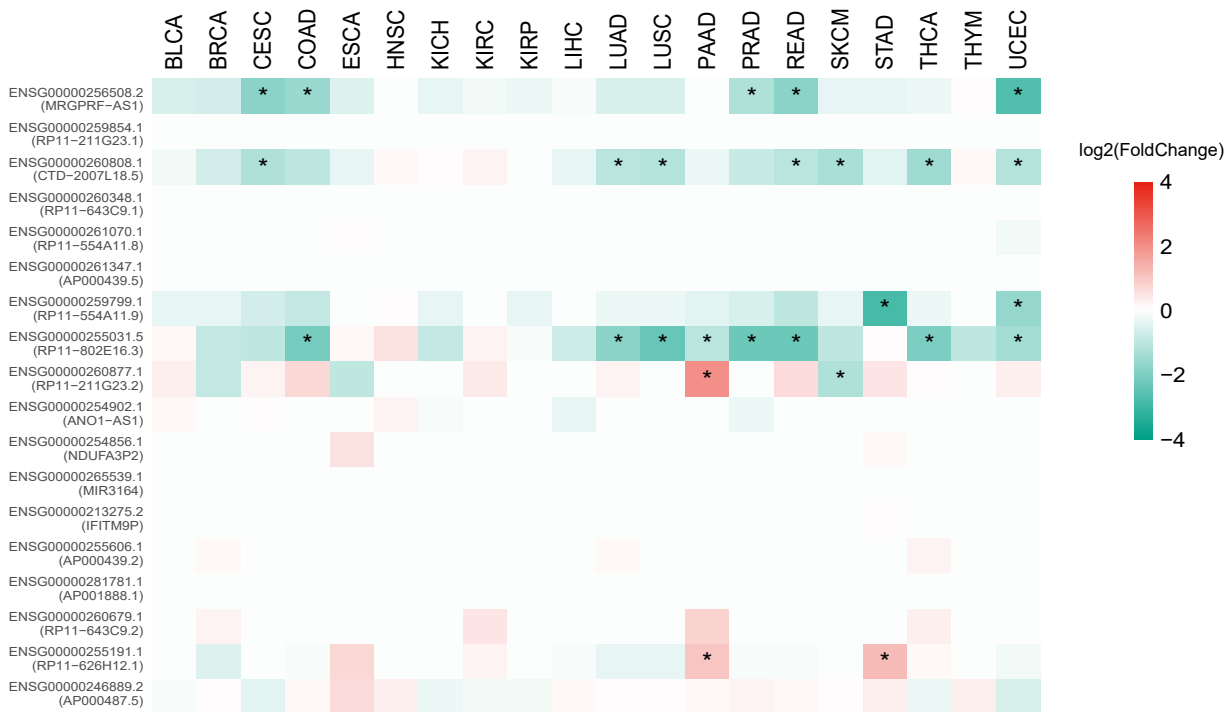

**B**

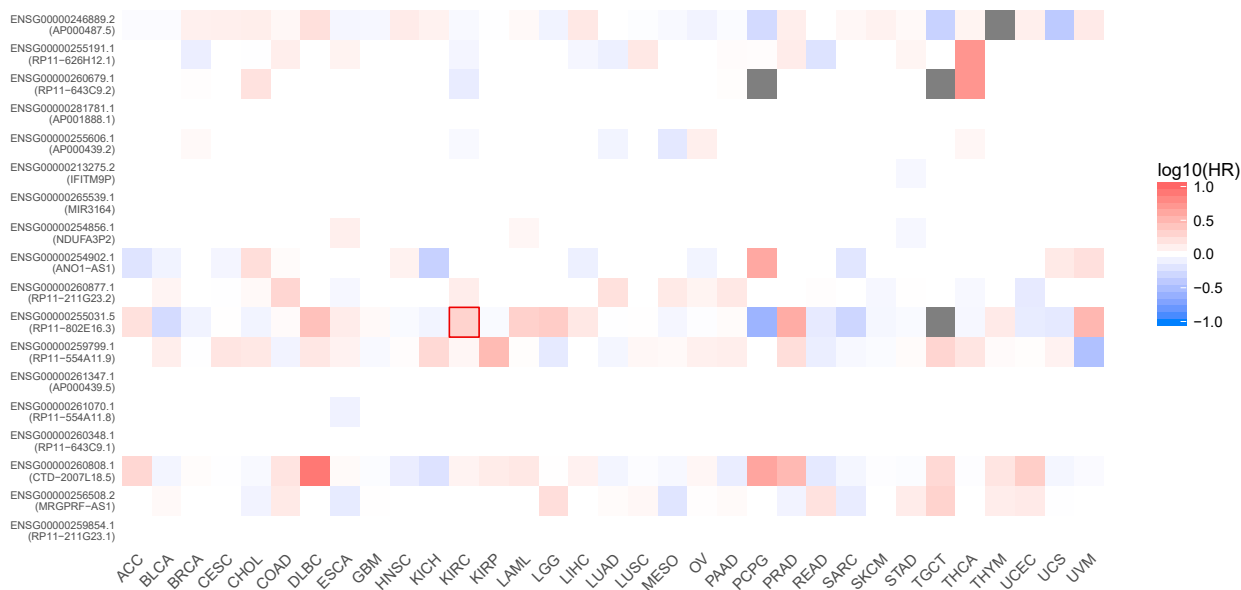

**C**

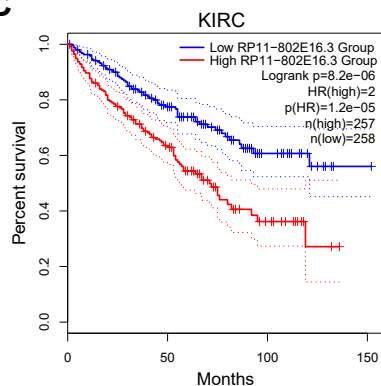

Supplement: S5 Fig — (A) Differential expression of the 18 non-coding CEDGs in tumor and normal samples based on TCGA tumors. (B) Survival analysis of non-coding CEDGs in TCGA tumors. (C) KM plots showing high RP11 − 802E16.3 expression is related to poor OS of KIRC patient. (PDF) [file pone.0324438.s005.pdf]

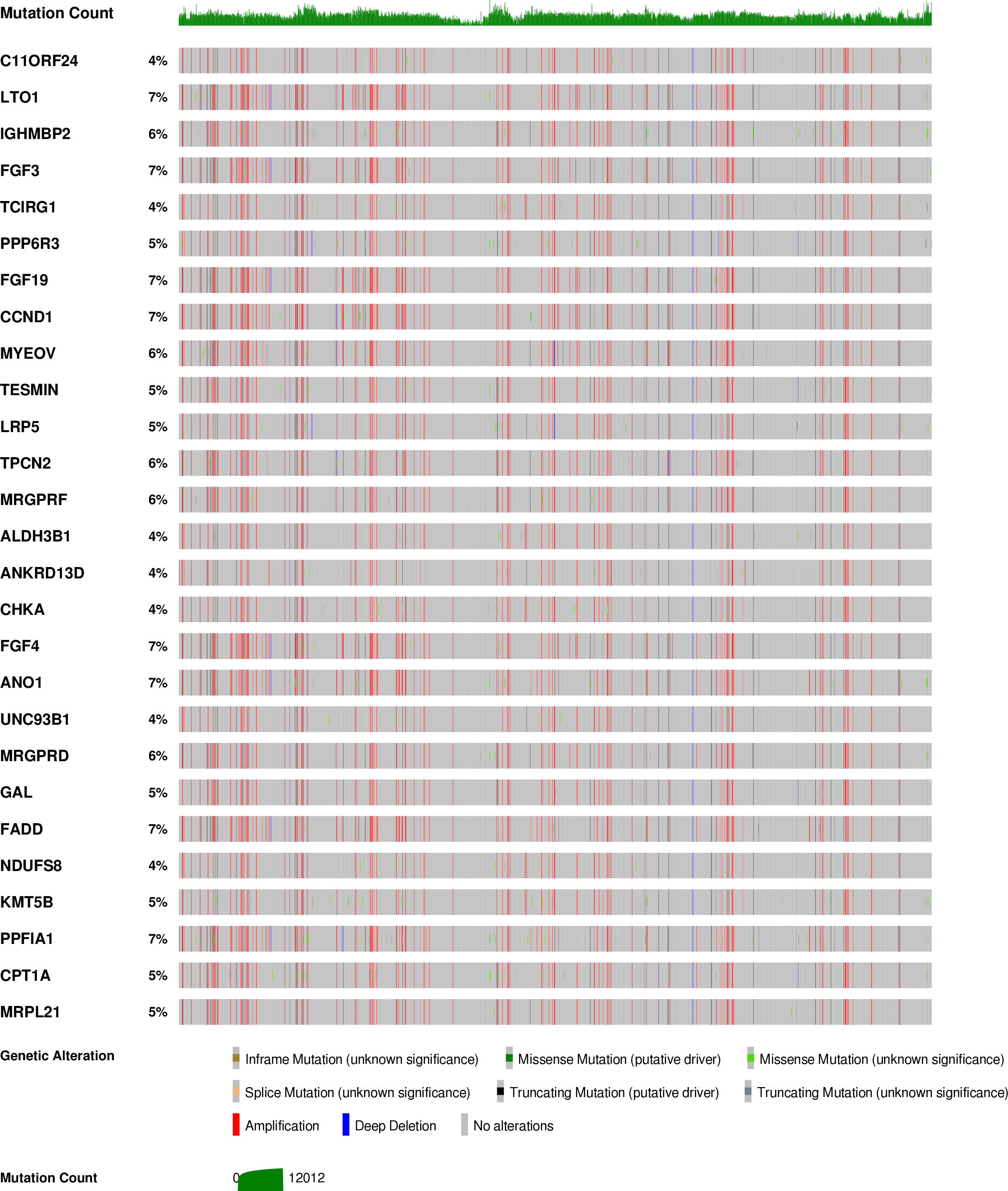

Supplement: S6 Fig — (TIF) [file pone.0324438.s006.tif]

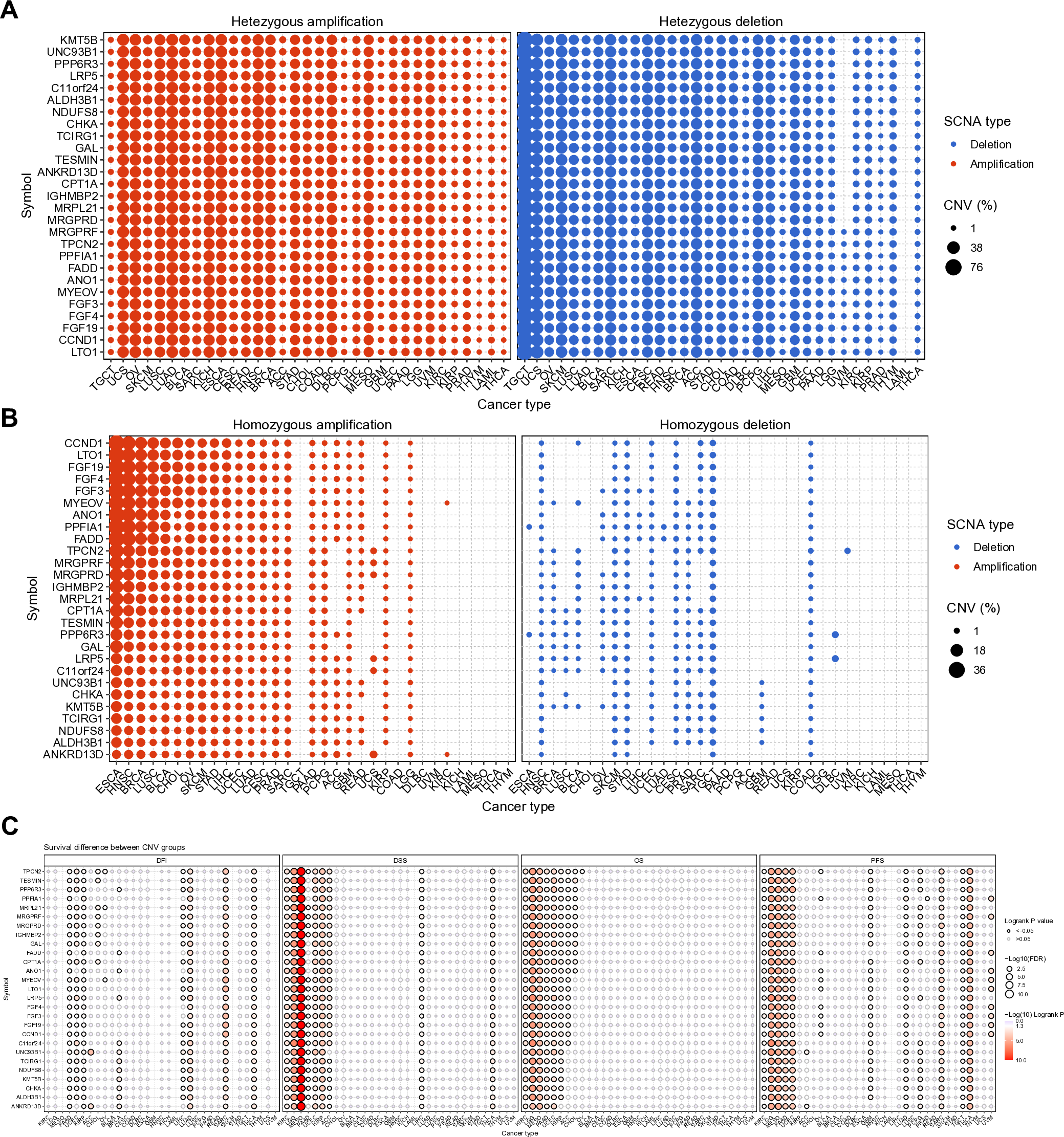

Supplement: S7 Fig — (A) Heterozygous CNV in each cancer. (B) Homozygous CNV in each cancer. (C) DFI, DSS, OS and PFS analysis in each cancer. (TIF) [file pone.0324438.s007.tif]

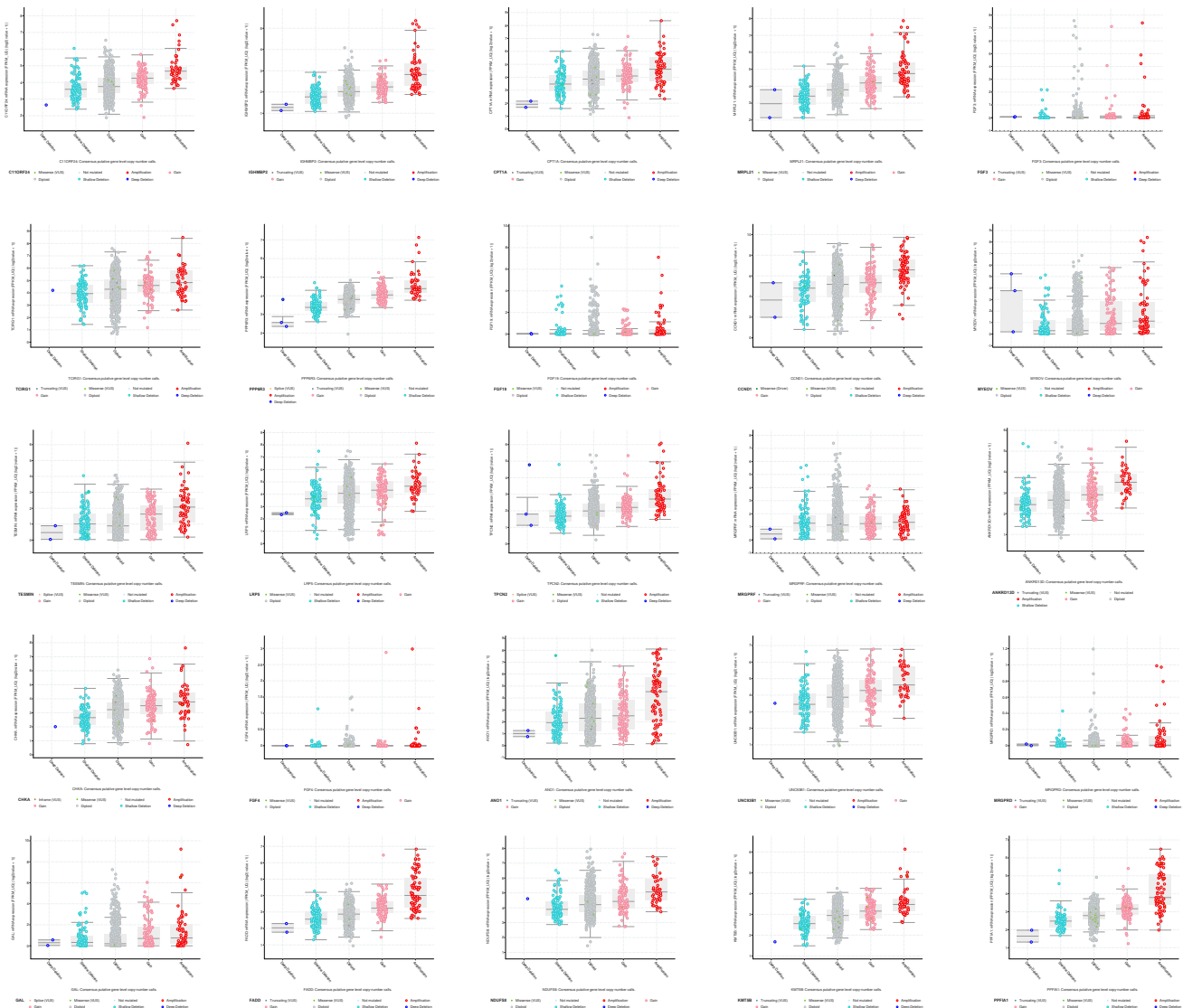

Supplement: S8 Fig — (PDF) [file pone.0324438.s008.pdf]

**A**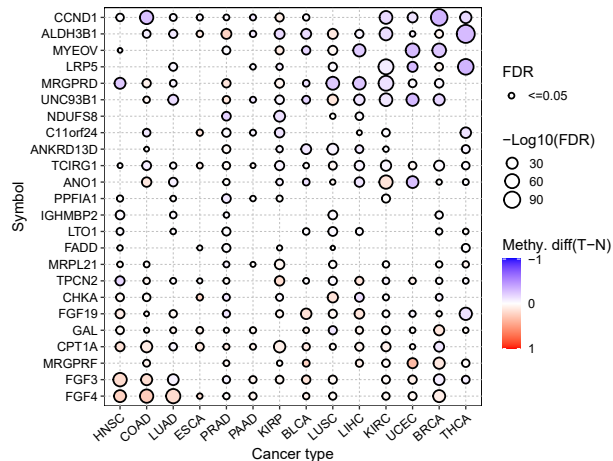**B**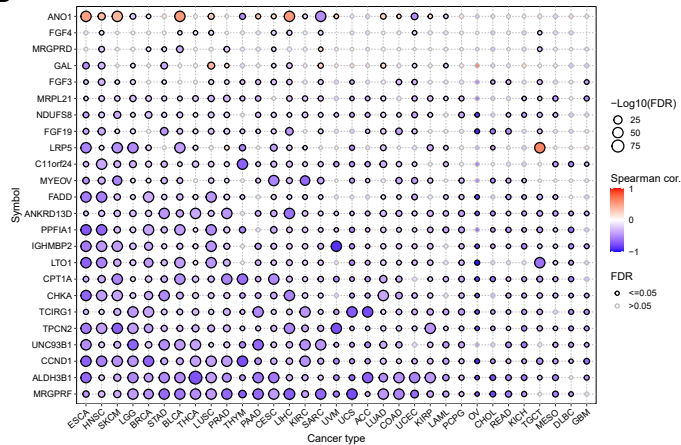**C**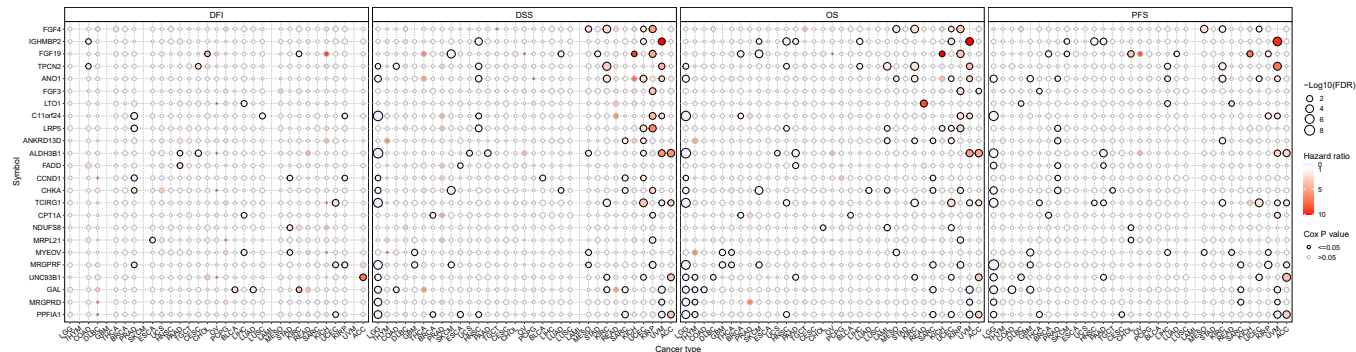

Supplement: S11 Fig — (A) Methylation difference of CEDGs between tumor and normal samples in each cancer. (B) Correlation between methylation and mRNA expression. (C) Survival difference between high and low methylation in each cancer. (PDF) [file pone.0324438.s011.pdf]

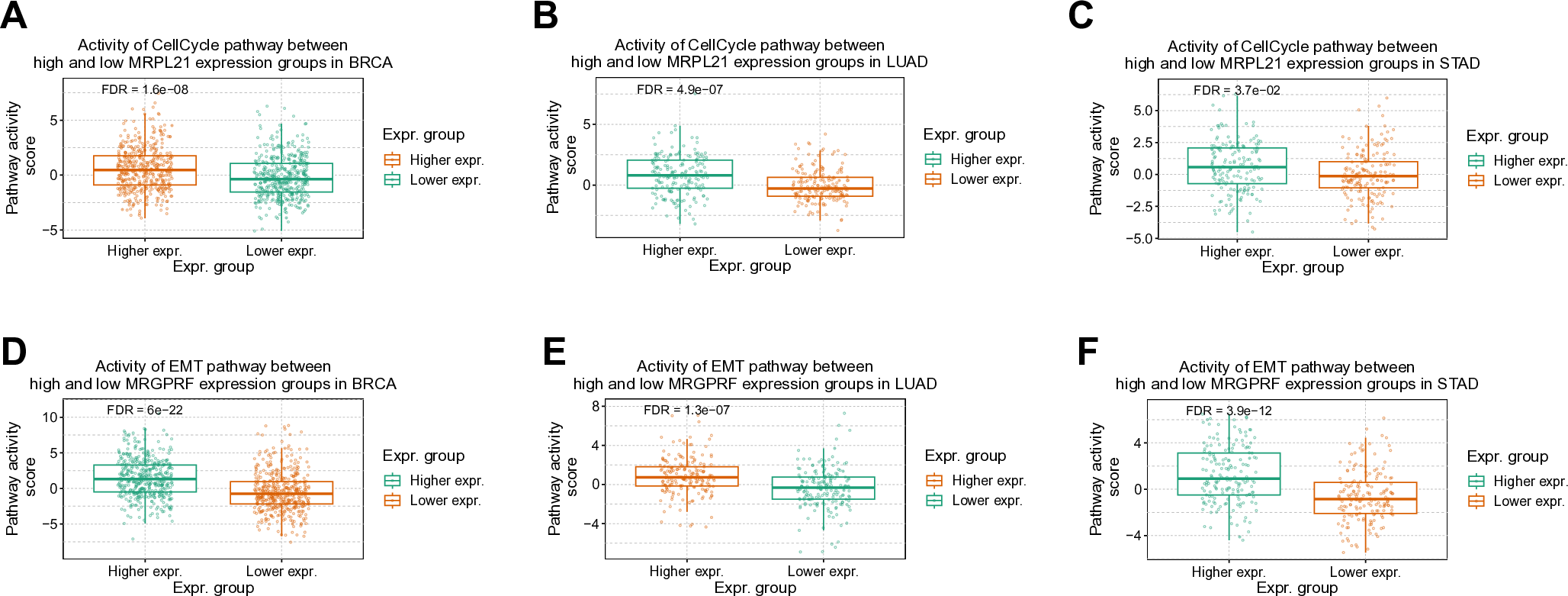

Supplement: S12 Fig — (A-C) Activity of cell cycle pathway between high and low expression group of MRPL21 in BRCA (A), LUAD (B) and STAD (C). (D-F) Activity of EMT pathway between high and low expression group of MRGPRF in BRCA (D), LUAD (E) and STAD (F). (TIF) [file pone.0324438.s012.tif]

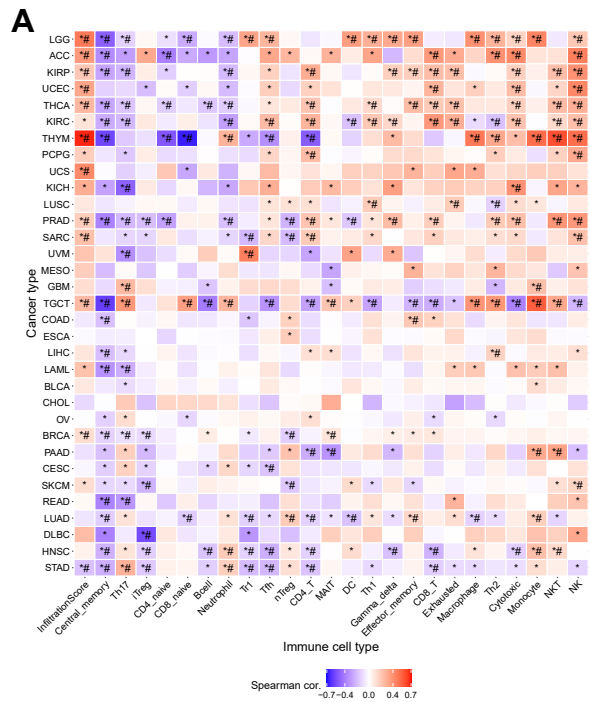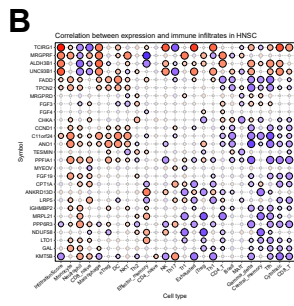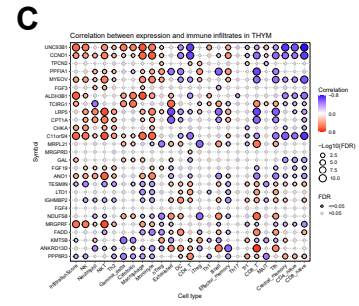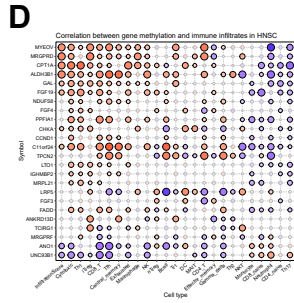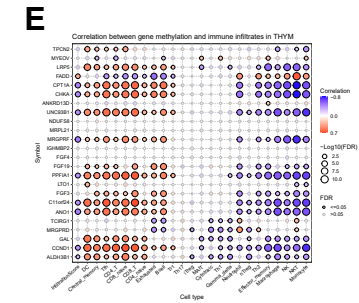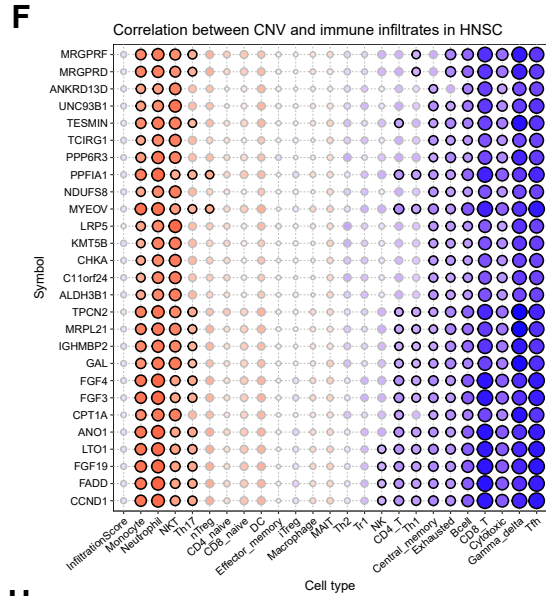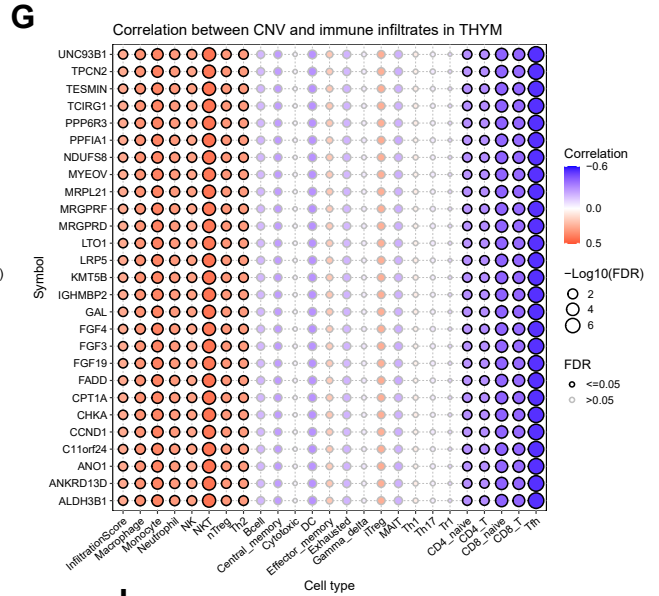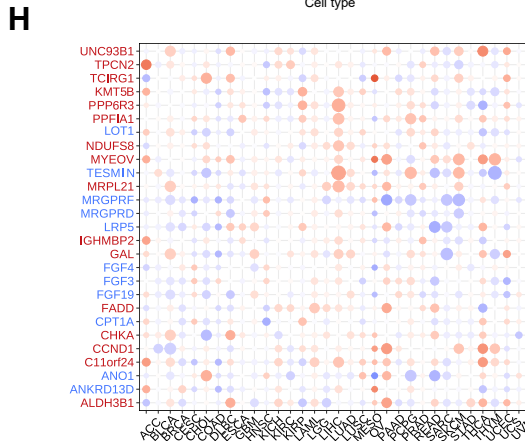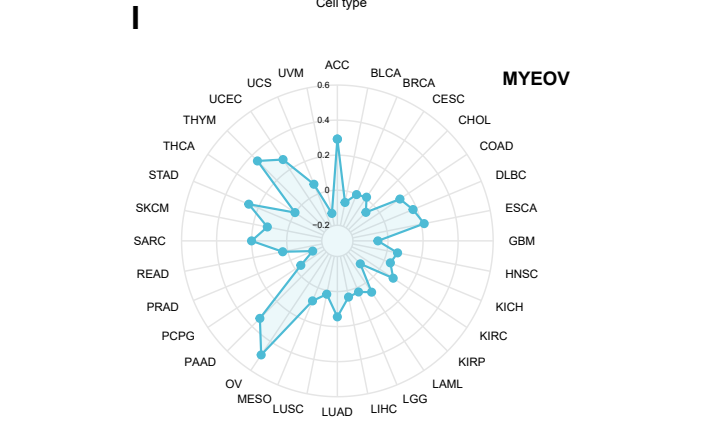

Supplement: S13 Fig — (A) The association between GSVA score and activity of cancer related pathways. (B, C) Correlation between CEDG expression and immune infiltrates in HNSC (B) and THYM (C). (D, E) Correlation between CEDG methylation and immune infiltrates in HNSC (D) and THYM (E). (F, G) Correlation between CEDG CNV and immune infiltrates in HNSC (F) and THYM (G). (H) The association between CEDGs and TMB in TCGA tumors. (I) Radar plot showing correlation of MYEOV and TMB in TCGA tumos. (PDF) [file pone.0324438.s013.pdf]

**A**

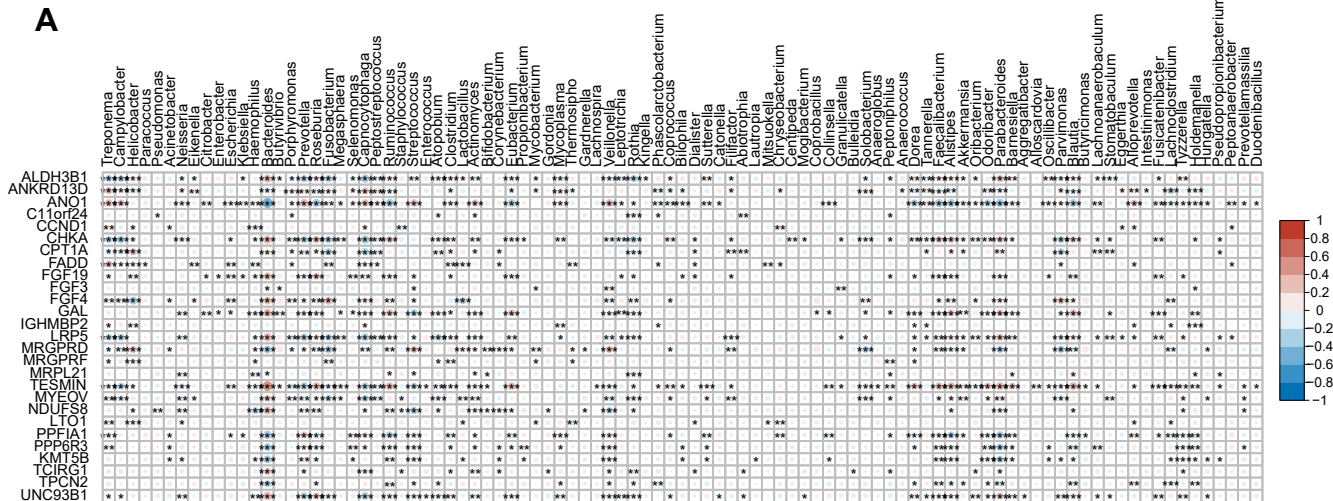

# B

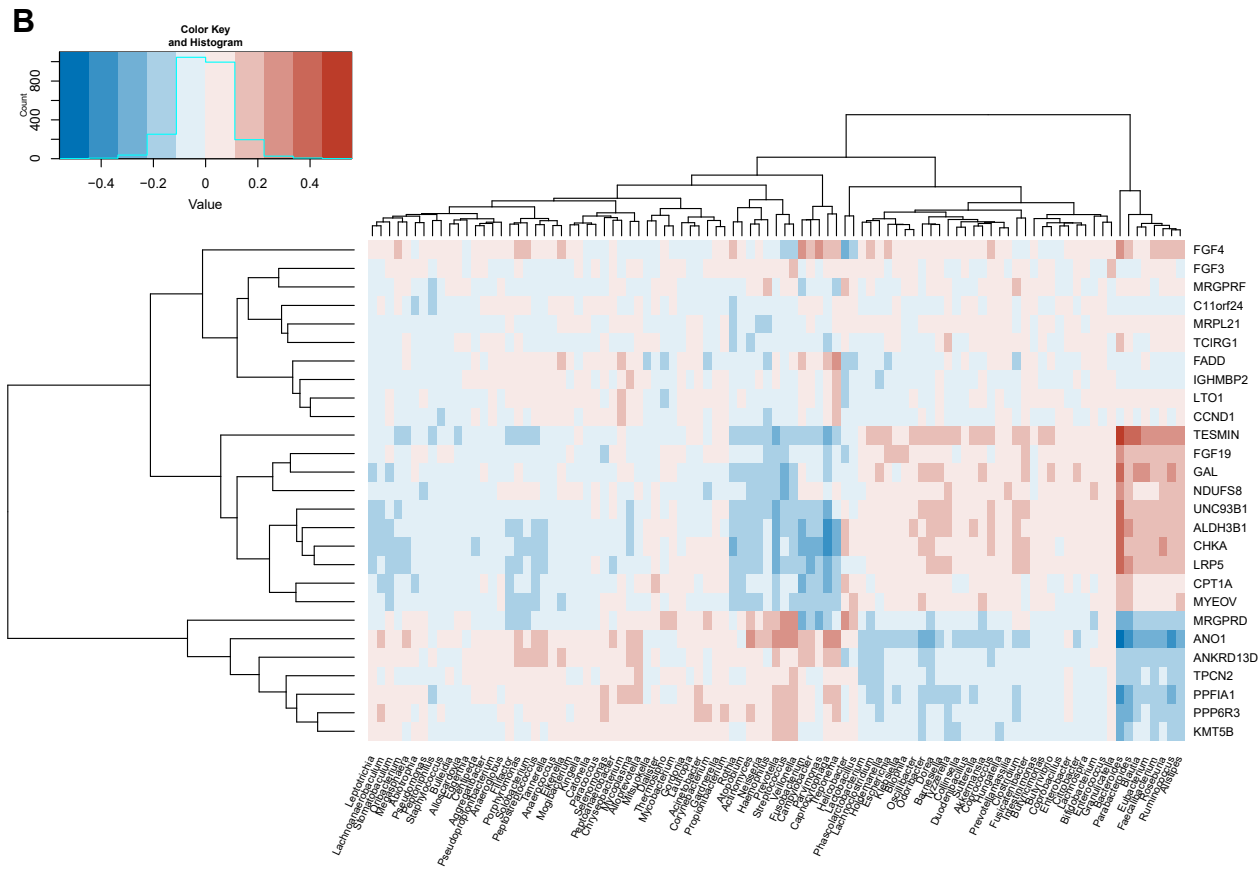

Supplement: S15 Fig — (A) Correlation of CEDG expression and microbiota abundance. (B) Cluster map of CEDG-microbiota correlation. (PDF) [file pone.0324438.s015.pdf]

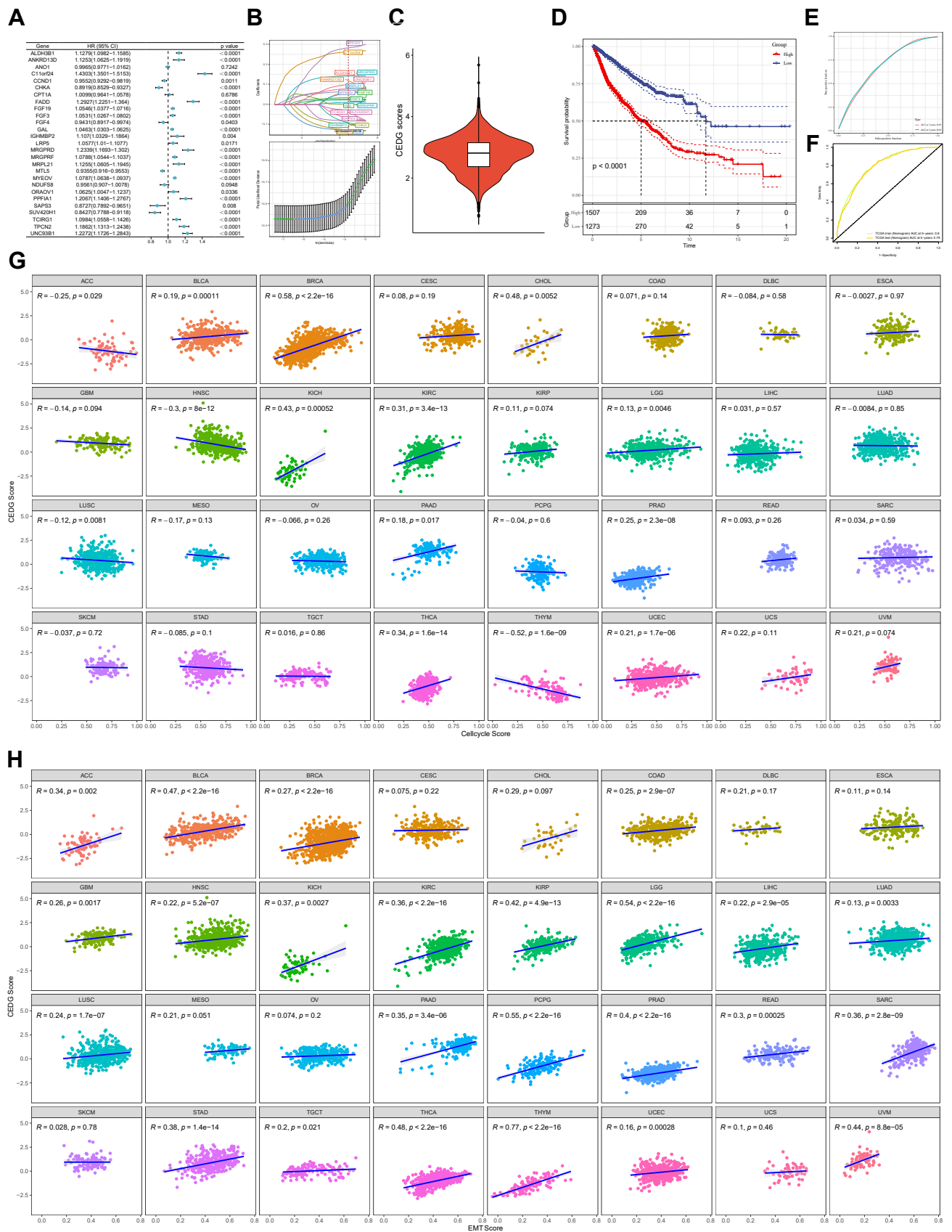

Supplement: S16 Fig — (A) Forest plot showing the hazard ratio (HR) of every single gene for the prognosis in TCGA-training cohort. (B) LASSO regression and pairwise correlation analysis in the TCGA pan-cancer training set. (C) The distribution of the CEDG scores. (D) KM plot showing high CEDG score was significantly correlated to poor OS of patients in TCGA-testing cohort. (E) Prognostic performance of the 17-gene CEDG signature in TCGA-testing cohort. (F) Prognostic performance of the nomogram. (G, H) Correlation of CEDG score with cell cycle. (G) and EMT (H) score in different cancer types. (PDF) [file pone.0324438.s016.pdf]

A

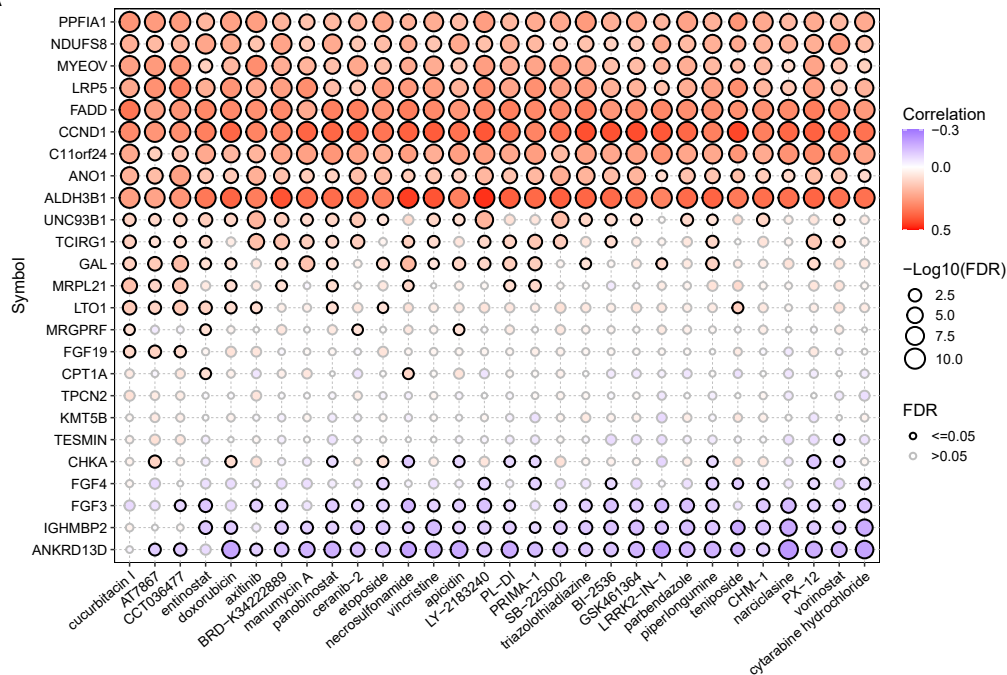

B

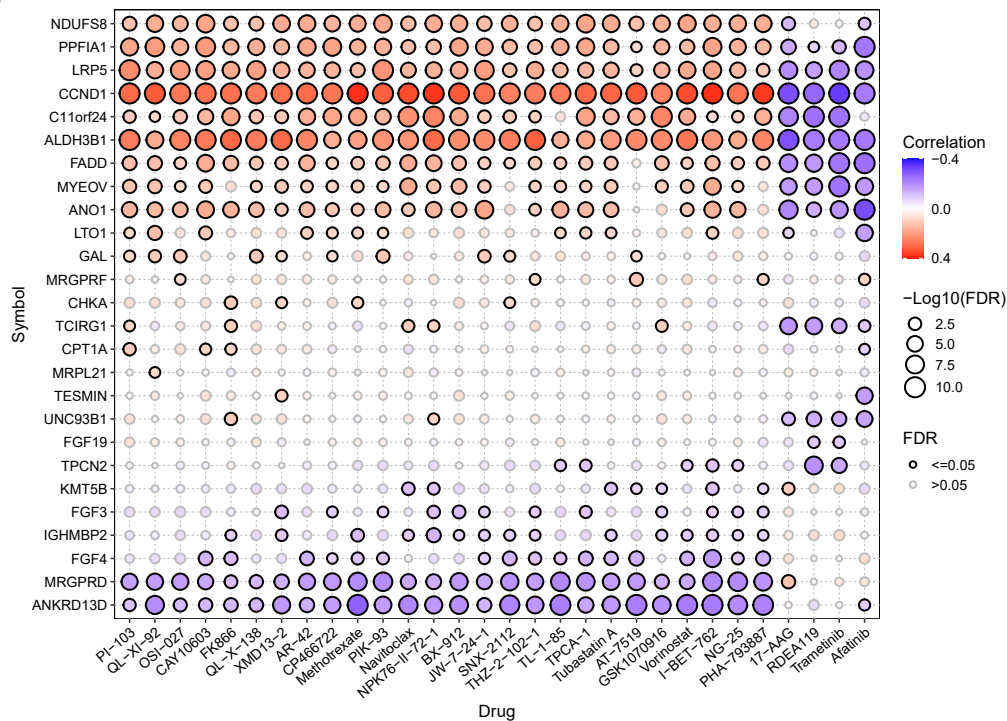

Supplement: S17 Fig — (PDF) [file pone.0324438.s017.pdf]

**A****HNSC**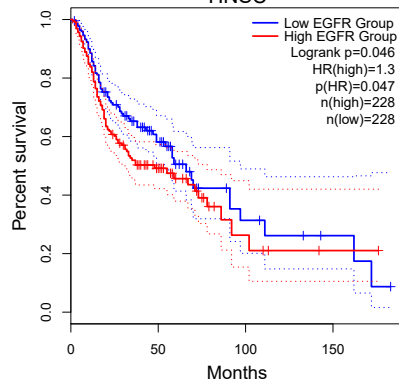**B****LIHC**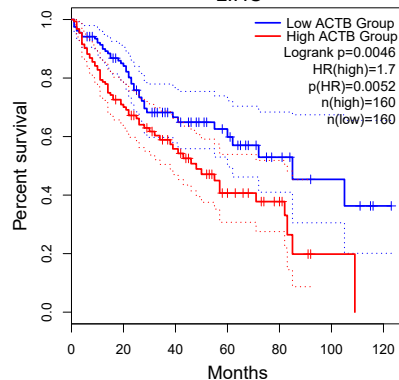**C****GBM**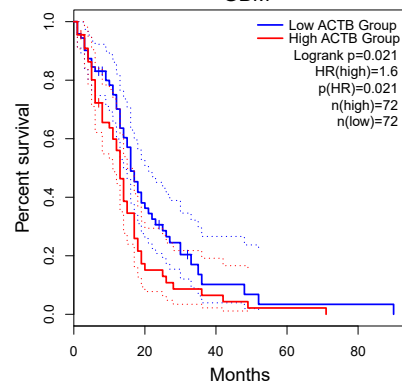**D****OV**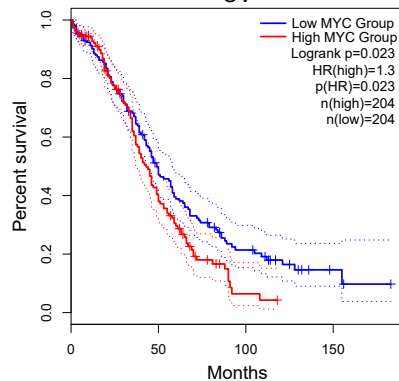**E****PAAD**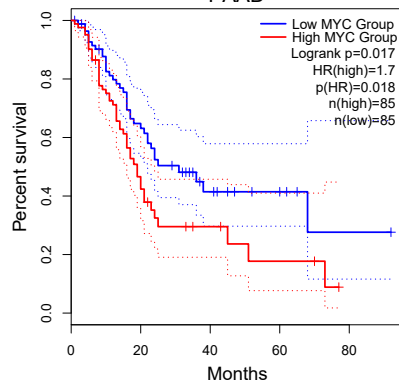**F****PRAD**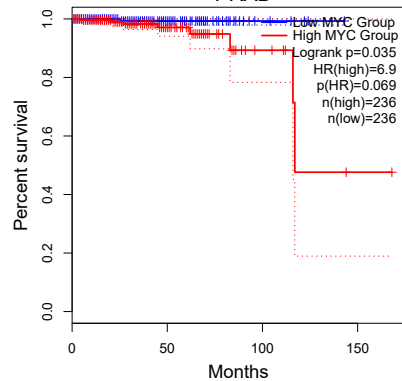

Supplement: S18 Fig — (PDF) [file pone.0324438.s018.pdf]

**A**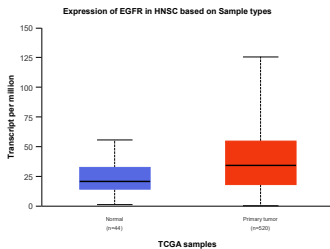**B**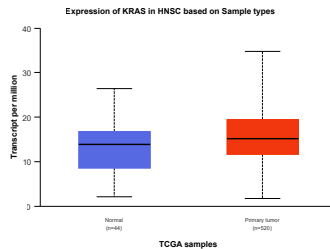**C**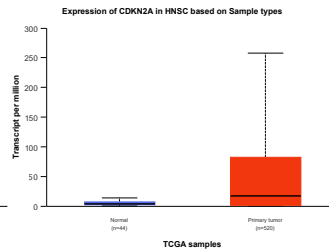**D**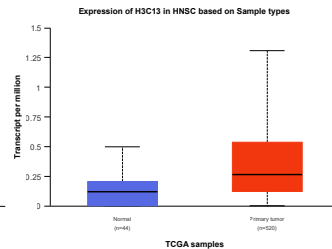**E****EGFR**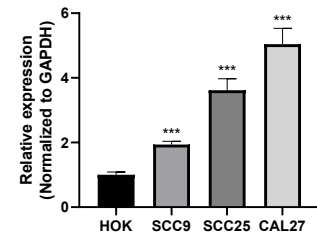**F****KRAS**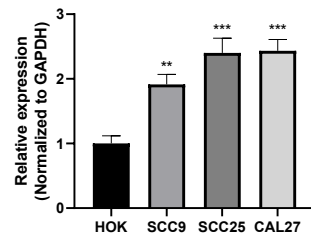**G****CDKN2A**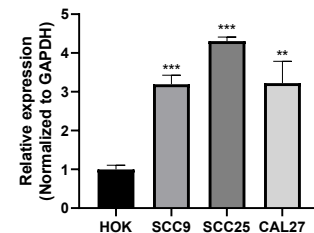**H****H3C13**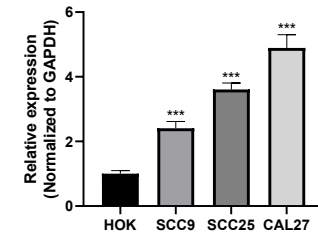

Supplement: S19 Fig — (A-D) The expression of EGFR (A), KRAS (B), CDKN2A (C) and H3C13 (D) in TCGA HNSC tumor and normal samples. (E-H) Validation of the expression of EGFR (E), KRAS (F), CDKN2A (G) and H3C13 (H) in normal and different HNSC cell lines. (PDF) [file pone.0324438.s019.pdf]

**A**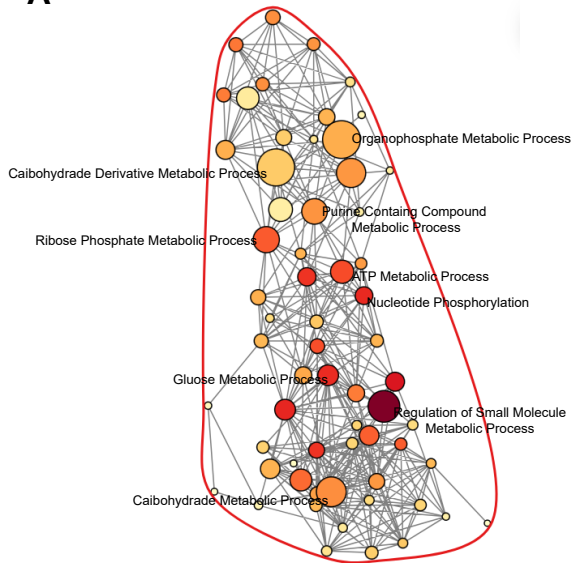 $-\log_{10}(\text{FDR})$ 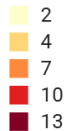

Gene Count

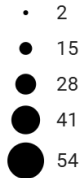**B**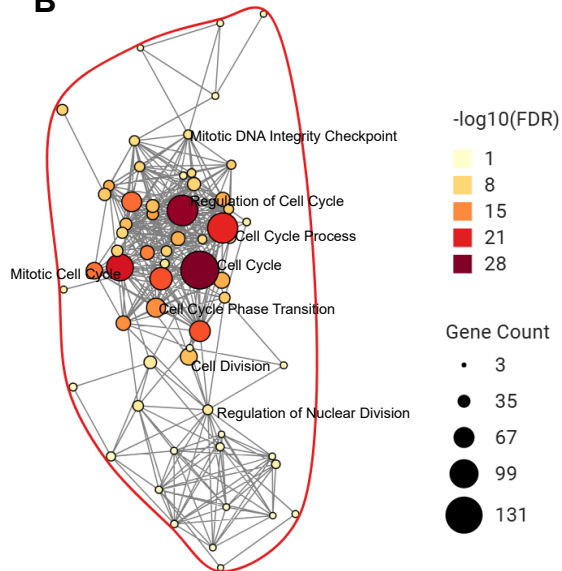 $-\log_{10}(\text{FDR})$ 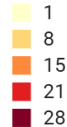

Gene Count

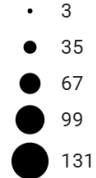

Supplement: S20 Fig — (A) Metabolic process network. (B) Cell cycle and division network. (PDF) [file pone.0324438.s020.pdf]

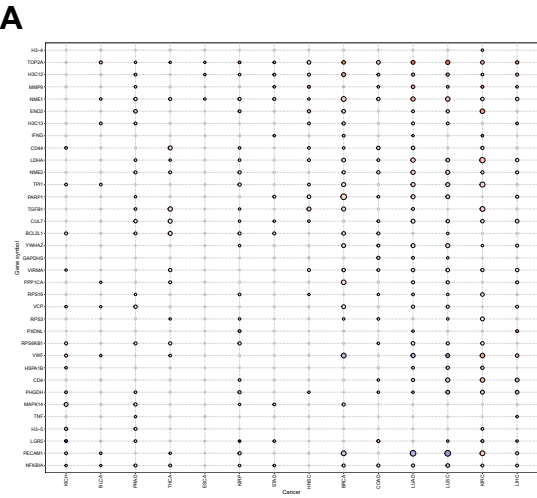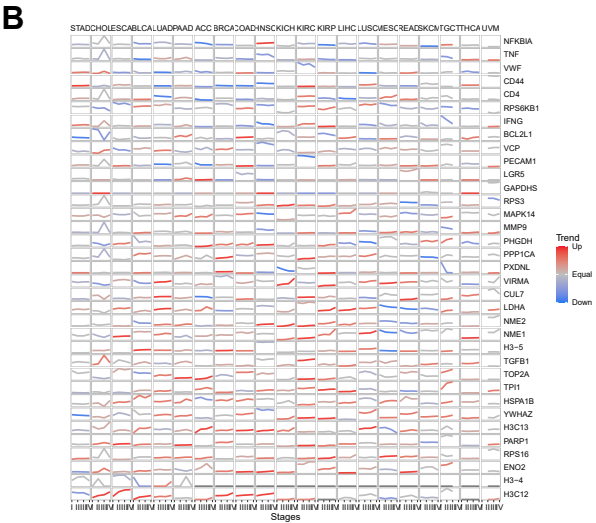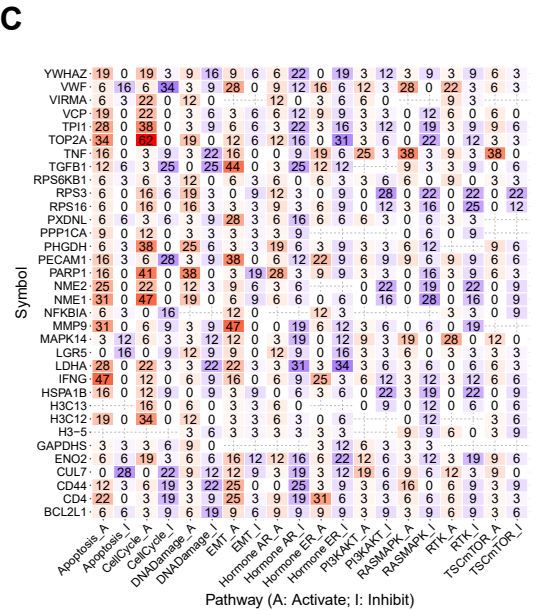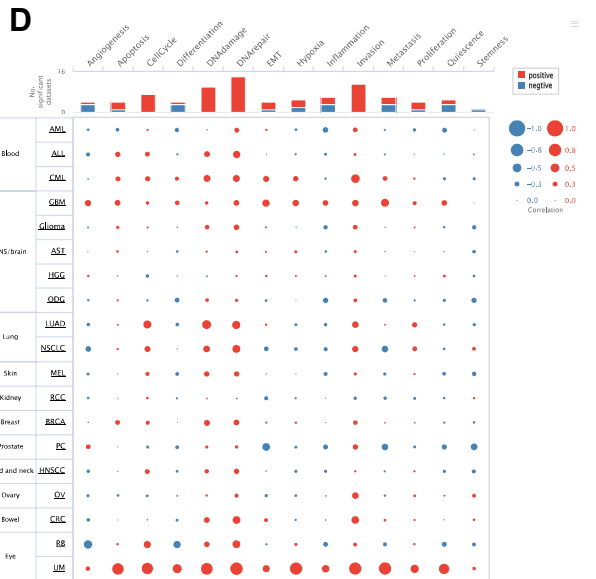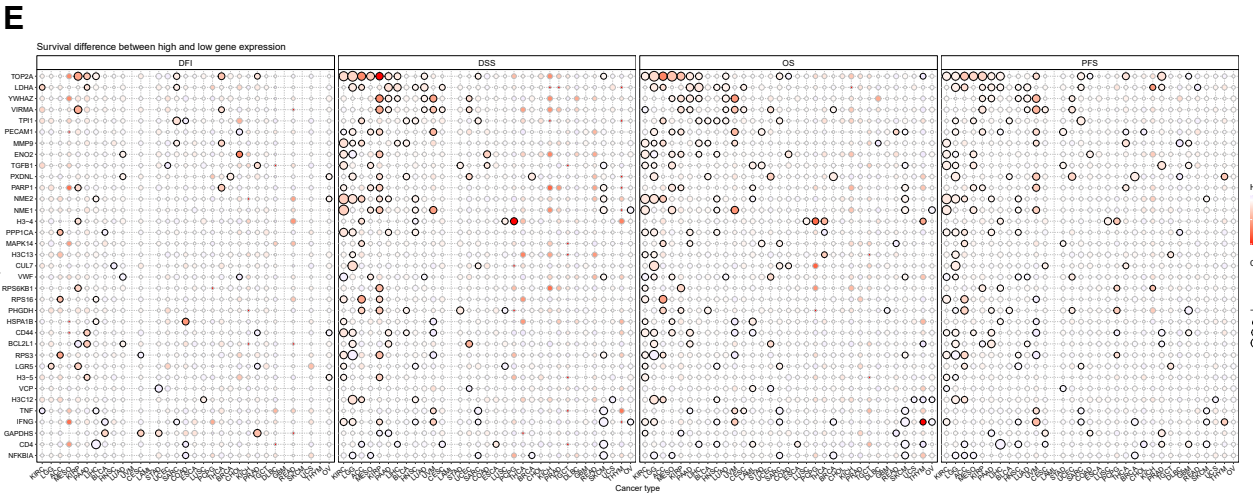

Supplement: S21 Fig — (A) Differential expression of the novel cancer-wide drivers in tumor and normal samples based on TCGA tumors. (B) Trend plot showing their expression tendency in pathologic stages of TCGA tumors. (C) The percentage of the effect of the novel cancer-wide drivers on cancer-related pathways. (D) Average correlations between the novel cancer-wide drivers and functional states in different cancers from CancerSEA. (E) Survival (DFI, DSS, OS and PFI) analysis of the novel cancer-wide drivers in TCGA tumors. (PDF) [file pone.0324438.s021.pdf]

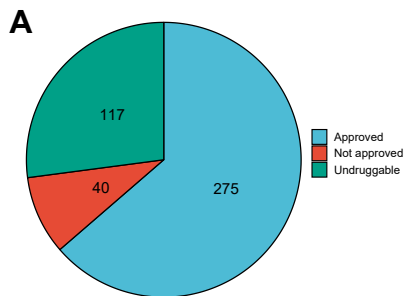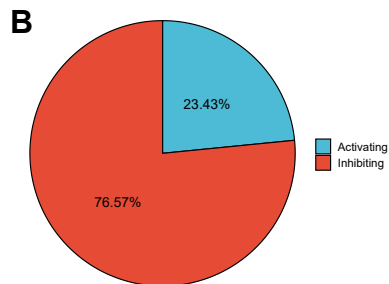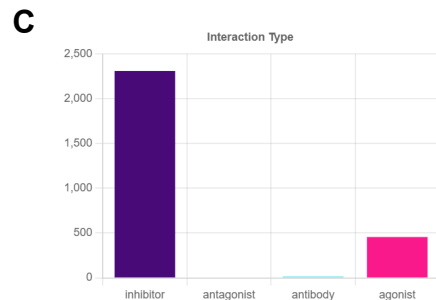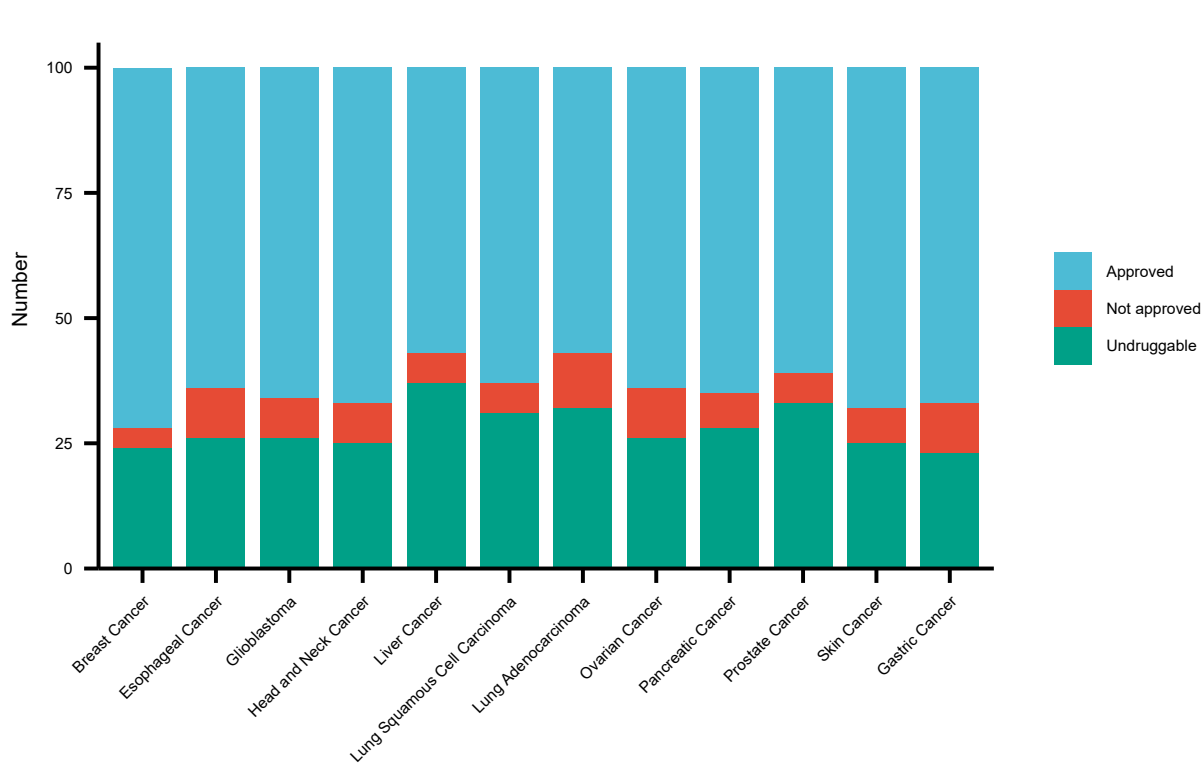

Supplement: S22 Fig — (A) Clinical actionability of the 432 candidate drivers. (B) Directionality distribution of the gene-drug interactions. (C) Interaction types of the gene-drug interactions. (D) Clinical actionability of the candidate drivers by cancer type. (PDF) [file pone.0324438.s022.pdf]

A

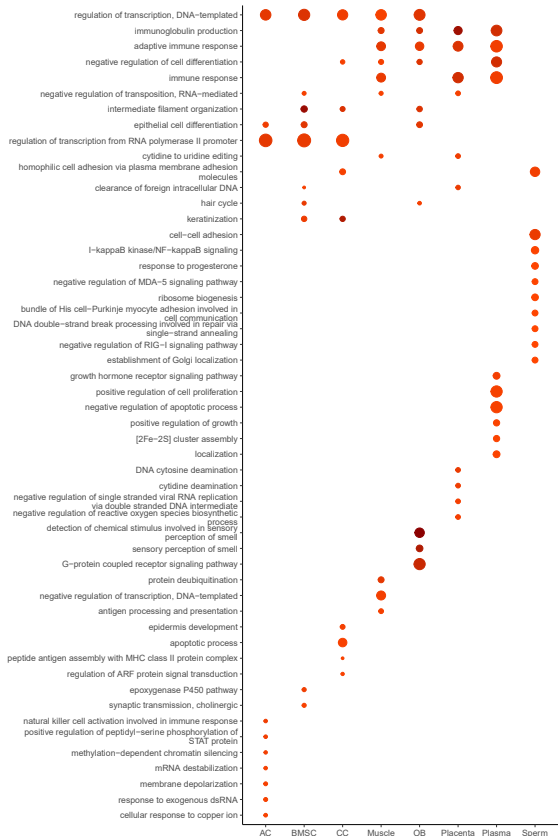

B

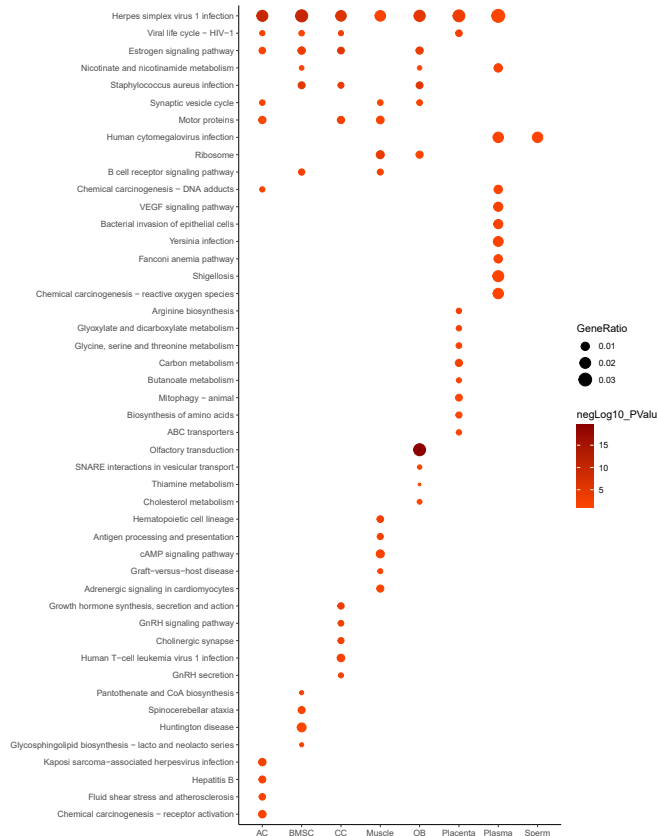

Supplement: S24 Fig — (A, B) Enriched term of GO (Biological process) (A) and KEGG pathway (B) of EEGs in normal samples. (PDF) [file pone.0324438.s024.pdf]
