# Supplementary material for: Identification and characterization of eccDNA-driven genes in humans
Source: PLoS One. 2025 Jun 6;20(6):e0324438. doi: 10.1371/journal.pone.0324438 (PMC12143510; doi:10.1371/journal.pone.0324438)
Supplement: S2 Fig — (A, B) GO (Biological process) (A) and KEGG pathway (B) analysis of EEGs to explore common pathways in cancers. (PDF) [file pone.0324438.s002.pdf]

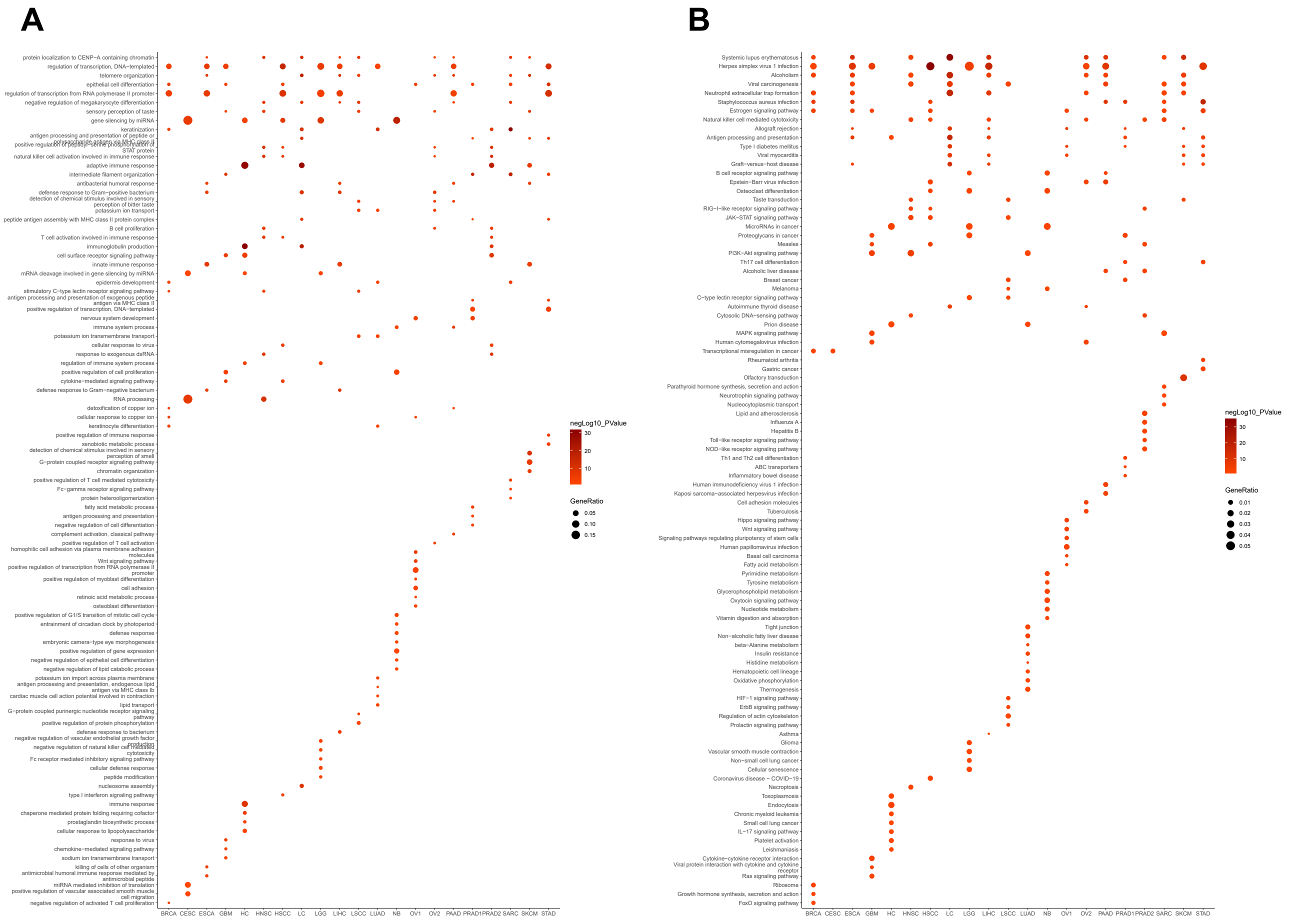

A
protein localization to CENP−A containing chromatin
regulation of transcription, DNA−templated
telomere organization
epithelial cell differentiation
regulation of transcription from RNA polymerase II promoter
negative regulation of megakaryocyte differentiation
sensory perception of taste
gene silencing by miRNA
keratinization
antigen processing and presentation of peptide or polysaccharide antigen via MHC class II
positive regulation of peptidyl−serine phosphorylation of STAT protein
natural killer cell activation involved in immune response
adaptive immune response
intermediate filament organization
antibacterial humoral response
defense response to Gram−positive bacterium
detection of chemical stimulus involved in sensory perception of bitter taste
potassium ion transport
peptide antigen assembly with MHC class II protein complex
B cell proliferation
T cell activation involved in immune response
immunoglobulin production
cell surface receptor signaling pathway
innate immune response
mRNA cleavage involved in gene silencing by miRNA
epidermis development
stimulatory C−type lectin receptor signaling pathway
antigen processing and presentation of exogenous peptide antigen via MHC class II
positive regulation of transcription, DNA−templated
nervous system development
immune system process
potassium ion transmembrane transport
cellular response to virus
response to exogenous dsRNA
regulation of immune system process
positive regulation of cell proliferation
cytokine−mediated signaling pathway
defense response to Gram−negative bacterium
RNA processing
detoxification of copper ion
cellular response to copper ion
keratinocyte differentiation
positive regulation of immune response
xenobiotic metabolic process
detection of chemical stimulus involved in sensory perception of smell
G−protein coupled receptor signaling pathway
chromatin organization
positive regulation of T cell mediated cytotoxicity
Fc−gamma receptor signaling pathway
protein heterooligomerization
fatty acid metabolic process
antigen processing and presentation
negative regulation of cell differentiation
complement activation, classical pathway
positive regulation of T cell activation
homophilic cell adhesion via plasma membrane adhesion molecules
Wnt signaling pathway
positive regulation of transcription from RNA polymerase II promoter
positive regulation of myoblast differentiation
cell adhesion
retinoic acid metabolic process
osteoblast differentiation
positive regulation of G1/S transition of mitotic cell cycle
entrainment of circadian clock by photoperiod
defense response
embryonic camera−type eye morphogenesis
positive regulation of gene expression
negative regulation of epithelial cell differentiation
negative regulation of lipid catabolic process
potassium ion import across plasma membrane
antigen processing and presentation, endogenous lipid antigen via MHC class Ib
cardiac muscle cell action potential involved in contraction
lipid transport
G−protein coupled purinergic nucleotide receptor signaling pathway
positive regulation of protein phosphorylation
defense response to bacterium
negative regulation of vascular endothelial growth factor production
negative regulation of natural killer cell mediated cytotoxicity
Fc receptor mediated inhibitory signaling pathway
cellular defense response
peptide modification
nucleosome assembly
type I interferon signaling pathway
immune response
chaperone mediated protein folding requiring cofactor
prostaglandin biosynthetic process
cellular response to lipopolysaccharide
response to virus
chemokine−mediated signaling pathway
sodium ion transmembrane transport
killing of cells of other organism
antimicrobial humoral immune response mediated by antimicrobial peptide
miRNA mediated inhibition of translation
positive regulation of vascular associated smooth muscle cell migration
negative regulation of activated T cell proliferation
BRCA CESC ESCA GBM HC HNSC HSCC LC LGG LIHC LSCC LUAD NB OV1 OV2 PAAD PRAD1 PRAD2 SARC SKCM STAD
negLog10_PValue
30
20
10
GeneRatio
0.05
0.10
0.15
B
Systemic lupus erythematosus
Herpes simplex virus 1 infection
Alcoholism
Viral carcinogenesis
Neutrophil extracellular trap formation
Staphylococcus aureus infection
Estrogen signaling pathway
Natural killer cell mediated cytotoxicity
Allograft rejection
Antigen processing and presentation
Type I diabetes mellitus
Viral myocarditis
Graft−versus−host disease
B cell receptor signaling pathway
Epstein−Barr virus infection
Osteoclast differentiation
Taste transduction
RIG−I−like receptor signaling pathway
JAK−STAT signaling pathway
MicroRNAs in cancer
Proteoglycans in cancer
Measles
PI3K−Akt signaling pathway
Th17 cell differentiation
Alcoholic liver disease
Breast cancer
Melanoma
C−type lectin receptor signaling pathway
Autoimmune thyroid disease
Cytosolic DNA−sensing pathway
Prion disease
MAPK signaling pathway
Human cytomegalovirus infection
Transcriptional misregulation in cancer
Rheumatoid arthritis
Gastric cancer
Olfactory transduction
Parathyroid hormone synthesis, secretion and action
Neurotrophin signaling pathway
Nucleocytoplasmic transport
Lipid and atherosclerosis
Influenza A
Hepatitis B
Toll−like receptor signaling pathway
NOD−like receptor signaling pathway
Th1 and Th2 cell differentiation
ABC transporters
Inflammatory bowel disease
Human immunodeficiency virus 1 infection
Kaposi sarcoma−associated herpesvirus infection
Cell adhesion molecules
Tuberculosis
Hippo signaling pathway
Wnt signaling pathway
Signaling pathways regulating pluripotency of stem cells
Human papillomavirus infection
Basal cell carcinoma
Fatty acid metabolism
Pyrimidine metabolism
Tyrosine metabolism
Glycerophospholipid metabolism
Oxytocin signaling pathway
Nucleotide metabolism
Vitamin digestion and absorption
Tight junction
Non−alcoholic fatty liver disease
beta−Alanine metabolism
Insulin resistance
Histidine metabolism
Hematopoietic cell lineage
Oxidative phosphorylation
Thermogenesis
HIF−1 signaling pathway
ErbB signaling pathway
Regulation of actin cytoskeleton
Prolactin signaling pathway
Asthma
Glioma
Vascular smooth muscle contraction
Non−small cell lung cancer
Cellular senescence
Coronavirus disease − COVID−19
Necroptosis
Toxoplasmosis
Endocytosis
Chronic myeloid leukemia
Small cell lung cancer
IL−17 signaling pathway
Platelet activation
Leishmaniasis
Cytokine−cytokine receptor interaction
Viral protein interaction with cytokine and cytokine receptor
Ras signaling pathway
Ribosome
Growth hormone synthesis, secretion and action
FoxO signaling pathway
BRCA CESC ESCA GBM HC HNSC HSCC LC LGG LIHC LSCC LUAD NB OV1 OV2 PAAD PRAD1 PRAD2 SARC SKCM STAD
negLog10_PValue
30
20
10
GeneRatio
0.01
0.02
0.03
0.04
0.05
