# Supplementary material for: Identification and characterization of eccDNA-driven genes in humans
Source: PLoS One. 2025 Jun 6;20(6):e0324438. doi: 10.1371/journal.pone.0324438 (PMC12143510; doi:10.1371/journal.pone.0324438)
Supplement: S10 Fig — (PDF) [file pone.0324438.s010.pdf]

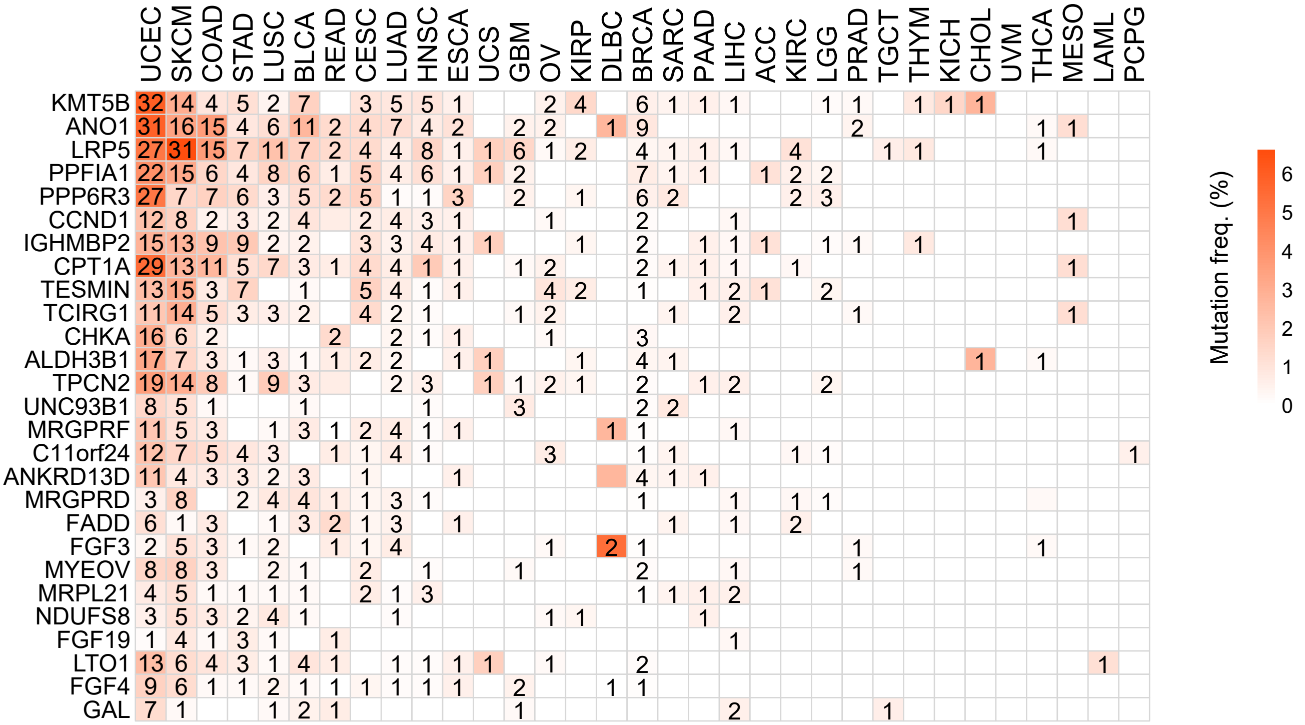
UCEC
SKCM
COAD
STAD
LUSC
BLCA
READ
CESC
LUAD
HNSC
ESCA
UCS
GBM
OV
KIRP
DLBC
BRCA
SARC
PAAD
LIHC
ACC
KIRC
LGG
PRAD
TGCT
THYM
KICH
CHOL
UVM
THCA
MESO
LAML
PCPG
KMT5B
ANO1
LRP5
PPFIA1
PPP6R3
CCND1
IGHMBP2
CPT1A
TESMIN
TCIRG1
CHKA
ALDH3B1
TPCN2
UNC93B1
MRGPRF
C11orf24
ANKRD13D
MRGPRD
FADD
FGF3
MYEOV
MRPL21
NDUFS8
FGF19
LTO1
FGF4
GAL
Mutation freq. (%)
6
5
4
3
2
1
0
