# Supplementary material for: Identification and characterization of eccDNA-driven genes in humans
Source: PLoS One. 2025 Jun 6;20(6):e0324438. doi: 10.1371/journal.pone.0324438 (PMC12143510; doi:10.1371/journal.pone.0324438)
Supplement: S14 Fig — (A) The difference of immune infiltration between gene set CNV groups. (B) The difference of immune infiltration between gene set SNV groups. (PDF) [file pone.0324438.s014.pdf]

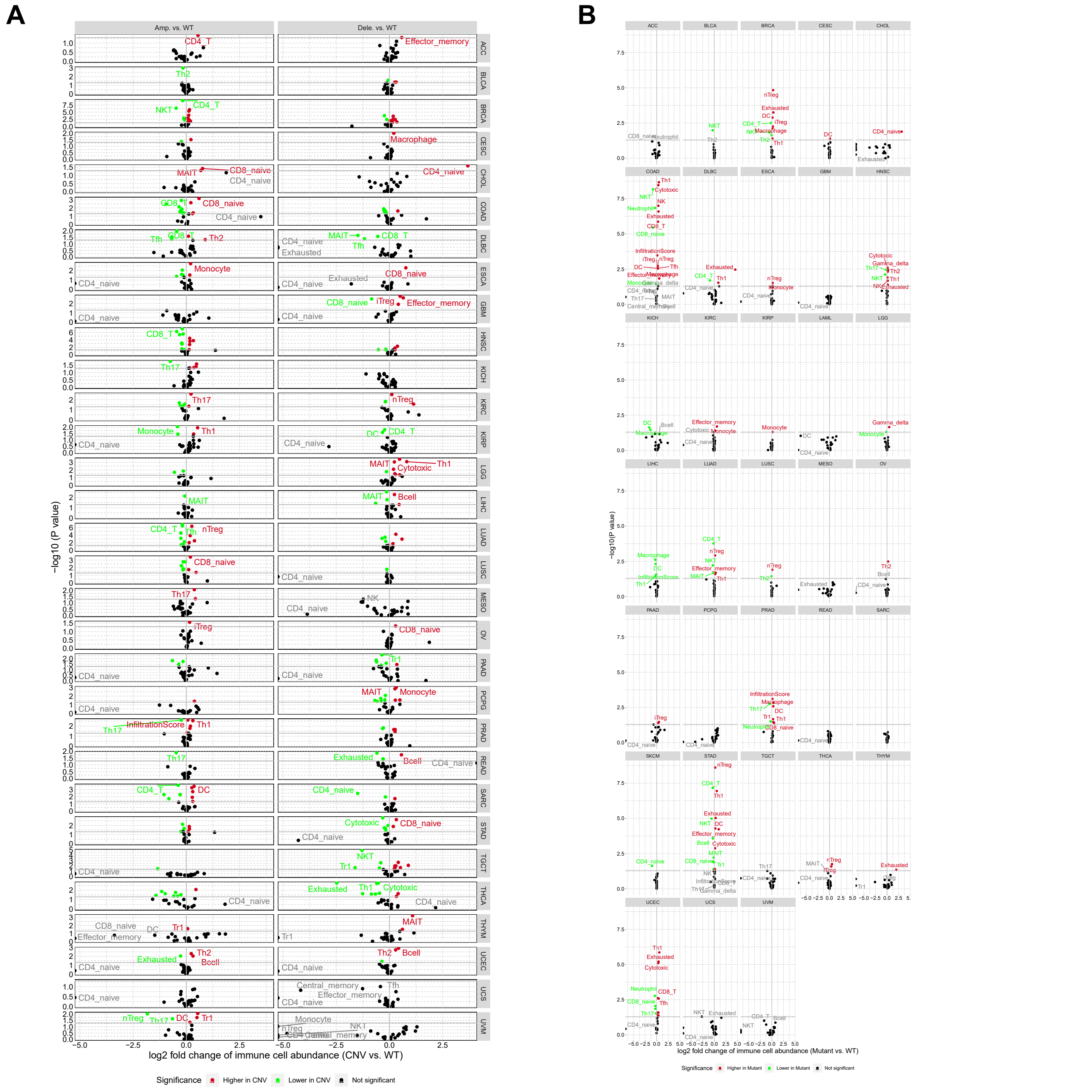

A
Amp. vs. WT
Dele. vs. WT
ACC
BLCA
BRCA
CESC
CHOL
COAD
DLBC
ESCA
GBM
HNSC
KICH
KIRC
KIRP
LGG
LIHC
LUAD
LUSC
MESO
OV
PAAD
PCPG
PRAD
READ
SARC
STAD
TGCT
THCA
THYM
UCEC
UCS
UVM
−log10 (P value)
log2 fold change of immune cell abundance (CNV vs. WT)
Significance
Higher in CNV
Lower in CNV
Not significant
B
LAML
LUSC
MESO
OV
SKCM
−log10(P value)
log2 fold change of immune cell abundance (Mutant vs. WT)
Significance
Higher in Mutant
Lower in Mutant
Not significant
