# Supplementary material for: Identification and characterization of eccDNA-driven genes in humans
Source: PLoS One. 2025 Jun 6;20(6):e0324438. doi: 10.1371/journal.pone.0324438 (PMC12143510; doi:10.1371/journal.pone.0324438)
Supplement: S9 Fig — (A) Mutation frequency of CEDGs. (B) Titv plot showing the distribution of transitions (Ti) and transversions (Tv) of CEDGs in each sample. (C) DFI, DSS, OS and PFS analysis in each cancer. (PDF) [file pone.0324438.s009.pdf]

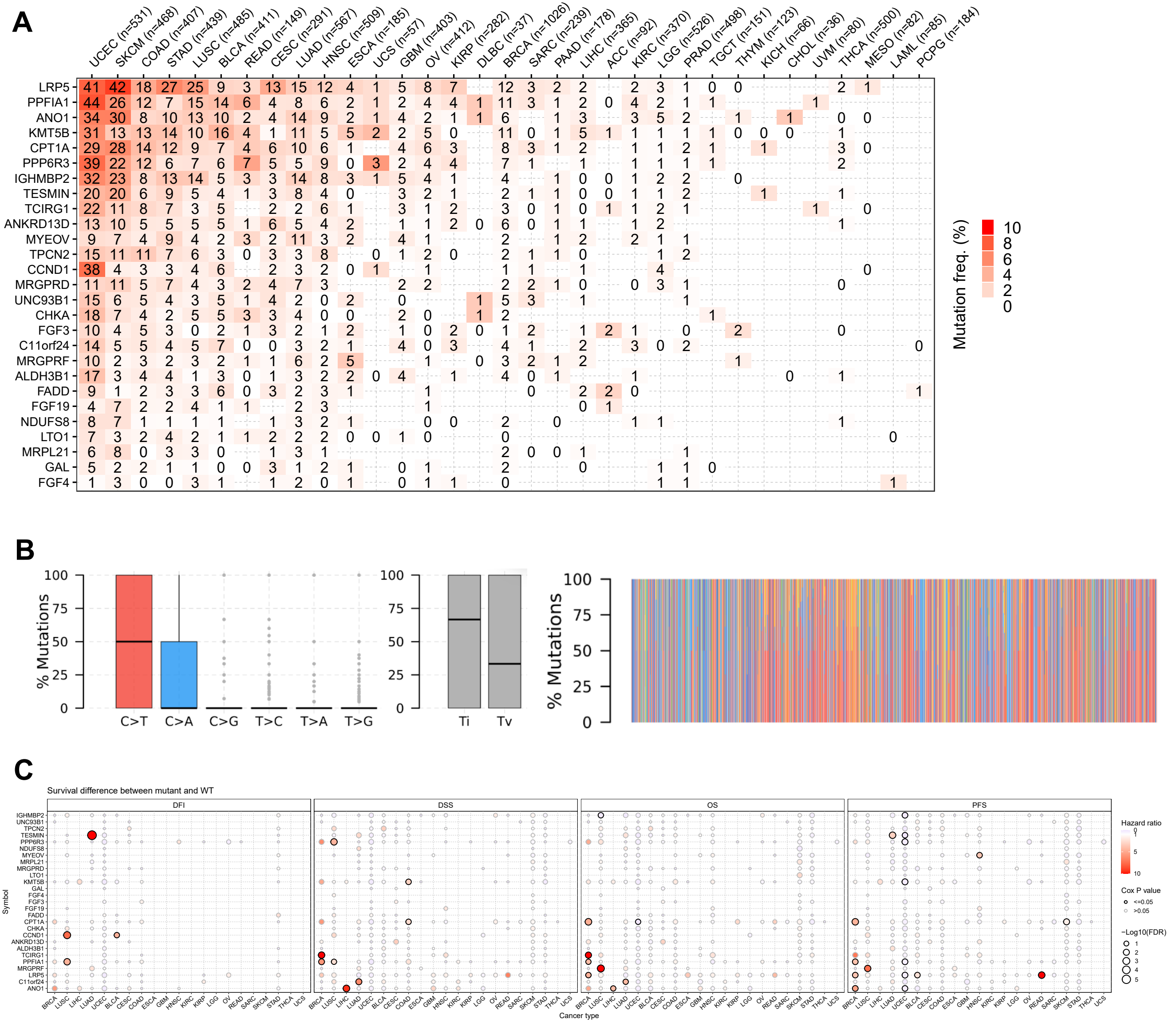
A
UCEC (n=531)
SKCM (n=468)
COAD (n=407)
STAD (n=439)
LUSC (n=485)
BLCA (n=411)
READ (n=149)
CESC (n=291)
LUAD (n=567)
HNSC (n=509)
ESCA (n=185)
UCS (n=57)
GBM (n=403)
OV (n=412)
KIRP (n=282)
DLBC (n=37)
BRCA (n=1026)
SARC (n=239)
PAAD (n=178)
LIHC (n=365)
ACC (n=92)
KIRC (n=370)
LGG (n=526)
PRAD (n=498)
TGCT (n=151)
THYM (n=123)
KICH (n=66)
CHOL (n=36)
UVM (n=80)
THCA (n=500)
MESO (n=82)
LAML (n=85)
PCPG (n=184)
LRP5
PPFIA1
ANO1
KMT5B
CPT1A
PPP6R3
IGHMBP2
TESMIN
TCIRG1
ANKRD13D
MYEOV
TPCN2
CCND1
MRGPRD
UNC93B1
CHKA
FGF3
C11orf24
MRGPRF
ALDH3B1
FADD
FGF19
NDUFS8
LTO1
MRPL21
GAL
FGF4
Mutation freq. (%)
10
8
6
4
2
0
B
% Mutations
100
75
50
25
0
C>T
C>A
C>G
T>C
T>A
T>G
Ti
Tv
C
Survival difference between mutant and WT
DFI
DSS
OS
PFS
Symbol
Cancer type
Hazard ratio
Cox P value
<=0.05
>0.05
−Log10(FDR)
