# Supplementary material for: Identification and characterization of eccDNA-driven genes in humans
Source: PLoS One. 2025 Jun 6;20(6):e0324438. doi: 10.1371/journal.pone.0324438 (PMC12143510; doi:10.1371/journal.pone.0324438)
Supplement: S23 Fig — (A, B) Enrichment analysis of ERGs (A) and EEGs (B) in healthy samples to related tissue. (C, D) GO (C) and KEGG pathway (D)analysis of ERGs to explore common pathways in health. (E, F) Disease enrichment analysis of ERGs (E) and EEGs (F) in non-cancerous diseases to related diseases. (G, H) Enriched ontology clusters across studies (G), by p value (H left) and cluster ID (H right) by ERGs in FGR. (I) Protein-protein interaction (PPI) network (left) and PPI MCODE components (right) by ERGs in FGR. (PDF) [file pone.0324438.s023.pdf]

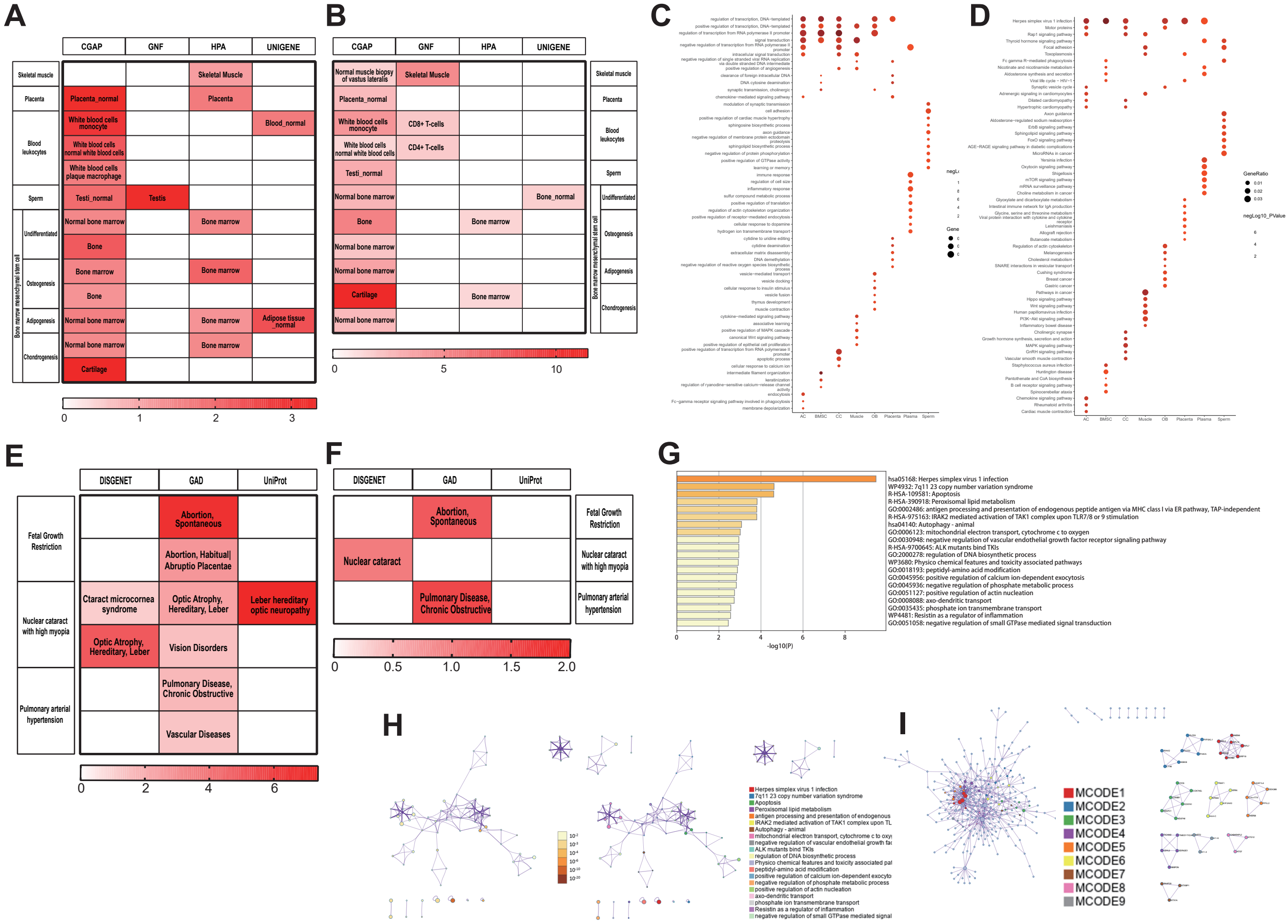

A
CGAP
GNF
HPA
UNIGENE
Skeletal muscle
Placenta
Blood leukocytes
Sperm
Bone marrow mesenchymal stem cell
Undifferentiated
Osteogenesis
Adipogenesis
Chondrogenesis
Skeletal Muscle
Placenta_normal
Placenta
White blood cells monocyte
Blood_normal
White blood cells normal white blood cells
White blood cells plaque macrophage
Testi_normal
Testis
Normal bone marrow
Bone marrow
Bone
Adipose tissue _normal
Cartilage
0
1
2
3
B
CGAP
GNF
HPA
UNIGENE
Normal muscle biopsy of vastus lateralis
Skeletal Muscle
Placenta_normal
White blood cells monocyte
CD8+ T-cells
White blood cells normal white blood cells
CD4+ T-cells
Testi_normal
Normal bone marrow
Bone_normal
Bone
Bone marrow
Cartilage
Skeletal muscle
Placenta
Blood leukocytes
Sperm
Bone marrow mesenchymal stem cell
Undifferentiated
Osteogenesis
Adipogenesis
Chondrogenesis
0
5
10
C
AC
BMSC
CC
Muscle
OB
Placenta
Plasma
Sperm
D
AC
BMSC
CC
Muscle
OB
Placenta
Plasma
Sperm
GeneRatio
0.01
0.02
0.03
negLog10_PValue
6
4
2
E
DISGENET
GAD
UniProt
Fetal Growth Restriction
Nuclear cataract with high myopia
Pulmonary arterial hypertension
Abortion, Spontaneous
Abortion, Habitual| Abruptio Placentae
Ctaract microcornea syndrome
Optic Atrophy, Hereditary, Leber
Leber hereditary optic neuropathy
Vision Disorders
Pulmonary Disease, Chronic Obstructive
Vascular Diseases
0
2
4
6
F
DISGENET
GAD
UniProt
Abortion, Spontaneous
Nuclear cataract
Pulmonary Disease, Chronic Obstructive
Fetal Growth Restriction
Nuclear cataract with high myopia
Pulmonary arterial hypertension
0
0.5
1.0
1.5
2.0
G
hsa05168: Herpes simplex virus 1 infection
WP4932: 7q11 23 copy number variation syndrome
R-HSA-109581: Apoptosis
R-HSA-390918: Peroxisomal lipid metabolism
GO:0002486: antigen processing and presentation of endogenous peptide antigen via MHC class I via ER pathway, TAP-independent
R-HSA-975163: IRAK2 mediated activation of TAK1 complex upon TLR7/8 or 9 stimulation
hsa04140: Autophagy - animal
GO:0006123: mitochondrial electron transport, cytochrome c to oxygen
GO:0030948: negative regulation of vascular endothelial growth factor receptor signaling pathway
R-HSA-9700645: ALK mutants bind TKIs
GO:2000278: regulation of DNA biosynthetic process
WP3680: Physico chemical features and toxicity associated pathways
GO:0018193: peptidyl-amino acid modification
GO:0045956: positive regulation of calcium ion-dependent exocytosis
GO:0045936: negative regulation of phosphate metabolic process
GO:0051127: positive regulation of actin nucleation
GO:0008088: axo-dendritic transport
GO:0035435: phosphate ion transmembrane transport
WP4481: Resistin as a regulator of inflammation
GO:0051058: negative regulation of small GTPase mediated signal transduction
0
2
4
6
8
-log10(P)
H
Herpes simplex virus 1 infection
7q11 23 copy number variation syndrome
Apoptosis
Peroxisomal lipid metabolism
antigen processing and presentation of endogenous
IRAK2 mediated activation of TAK1 complex upon TL
Autophagy - animal
mitochondrial electron transport, cytochrome c to oxy
negative regulation of vascular endothelial growth fa
ALK mutants bind TKIs
regulation of DNA biosynthetic process
Physico chemical features and toxicity associated pat
peptidyl-amino acid modification
positive regulation of calcium ion-dependent exocyto
negative regulation of phosphate metabolic process
positive regulation of actin nucleation
axo-dendritic transport
phosphate ion transmembrane transport
Resistin as a regulator of inflammation
negative regulation of small GTPase mediated signal
I
MCODE1
MCODE2
MCODE3
MCODE4
MCODE5
MCODE6
MCODE7
MCODE8
MCODE9
